# Supplementary material for: Autophagy-Dependent Generation of Free Fatty Acids Is Critical for Normal Neutrophil Differentiation
Source: Immunity. 2017 Sep 19;47(3):466–480.e5. doi: 10.1016/j.immuni.2017.08.005 (PMC5610174; doi:10.1016/j.immuni.2017.08.005)
Supplement: Document S1. Figures S1–S7 and Table S1 [file mmc1.pdf]

Immunity, Volume *47*

Supplemental Information

Autophagy-Dependent Generation of Free Fatty Acids Is Critical for Normal Neutrophil Differentiation

Thomas Riffelmacher, Alexander Clarke, Felix C. Richter, Amanda Stranks, Sumeet Pandey, Sara Danielli, Philip Hublitz, Zhanru Yu, Errin Johnson, Tobias Schwerd, James McCullagh, Holm Uhlig, Sten Eirik W. Jacobsen, and Anna Katharina Simon

Figure S1

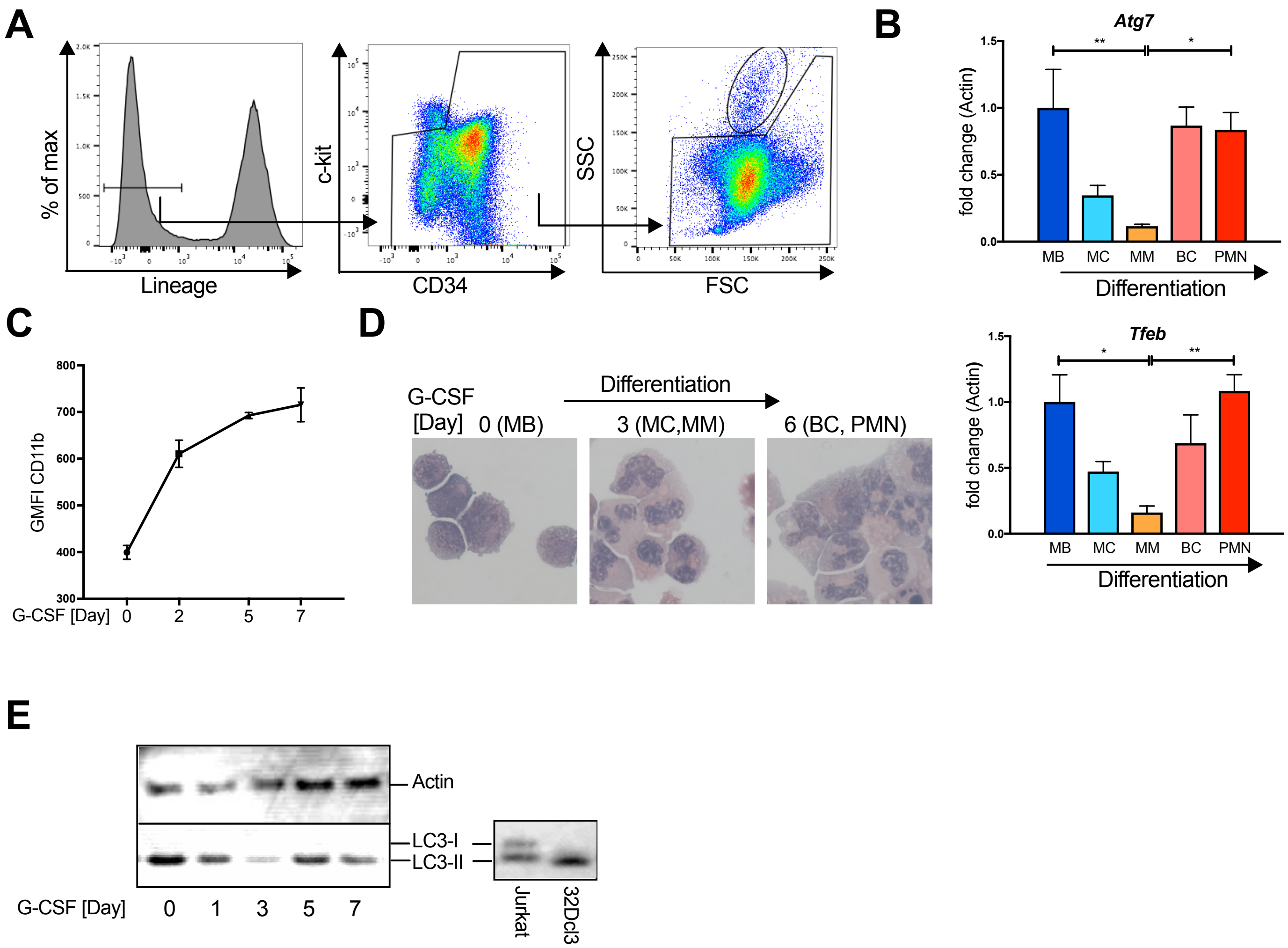
A
% of max
Lineage
c-kit
CD34
SSC
FSC
B
Atg7
fold change (Actin)
MB
MC
MM
BC
PMN
Differentiation
Tfeb
fold change (Actin)
MB
MC
MM
BC
PMN
Differentiation
C
GMFI CD11b
G-CSF [Day]
D
G-CSF
[Day]
Differentiation
0 (MB)
3 (MC,MM)
6 (BC, PMN)
E
Actin
LC3-I
LC3-II
G-CSF [Day]
0
1
3
5
7
Jurkat
32Dcl3

Figure S2

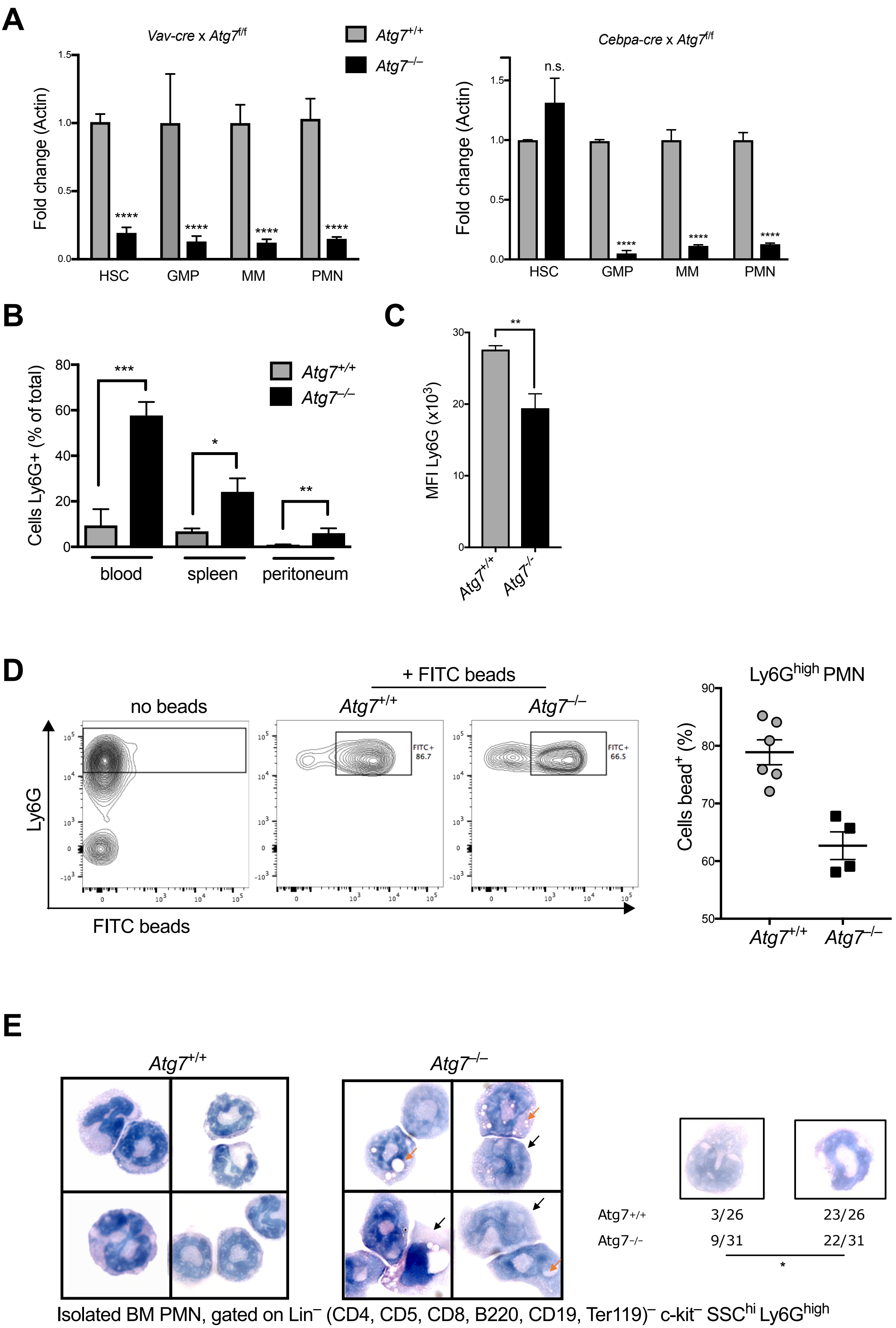
A
Vav-cre x Atg7f/f
Cebpa-cre x Atg7f/f
Atg7+/+
Atg7–/–
Fold change (Actin)
n.s.
****
HSC
GMP
MM
PMN
B
Cells Ly6G+ (% of total)
***
*
**
blood
spleen
peritoneum
C
MFI Ly6G (x10³)
**
Atg7+/+
Atg7-/-
D
no beads
+ FITC beads
Ly6G
FITC beads
FITC+ 86.7
FITC+ 66.5
Ly6Ghigh PMN
Cells bead+ (%)
E
Atg7+/+ 3/26 23/26
Atg7-/- 9/31 22/31
*
Isolated BM PMN, gated on Lin– (CD4, CD5, CD8, B220, CD19, Ter119)– c-kit– SSChi Ly6Ghigh

Figure S3

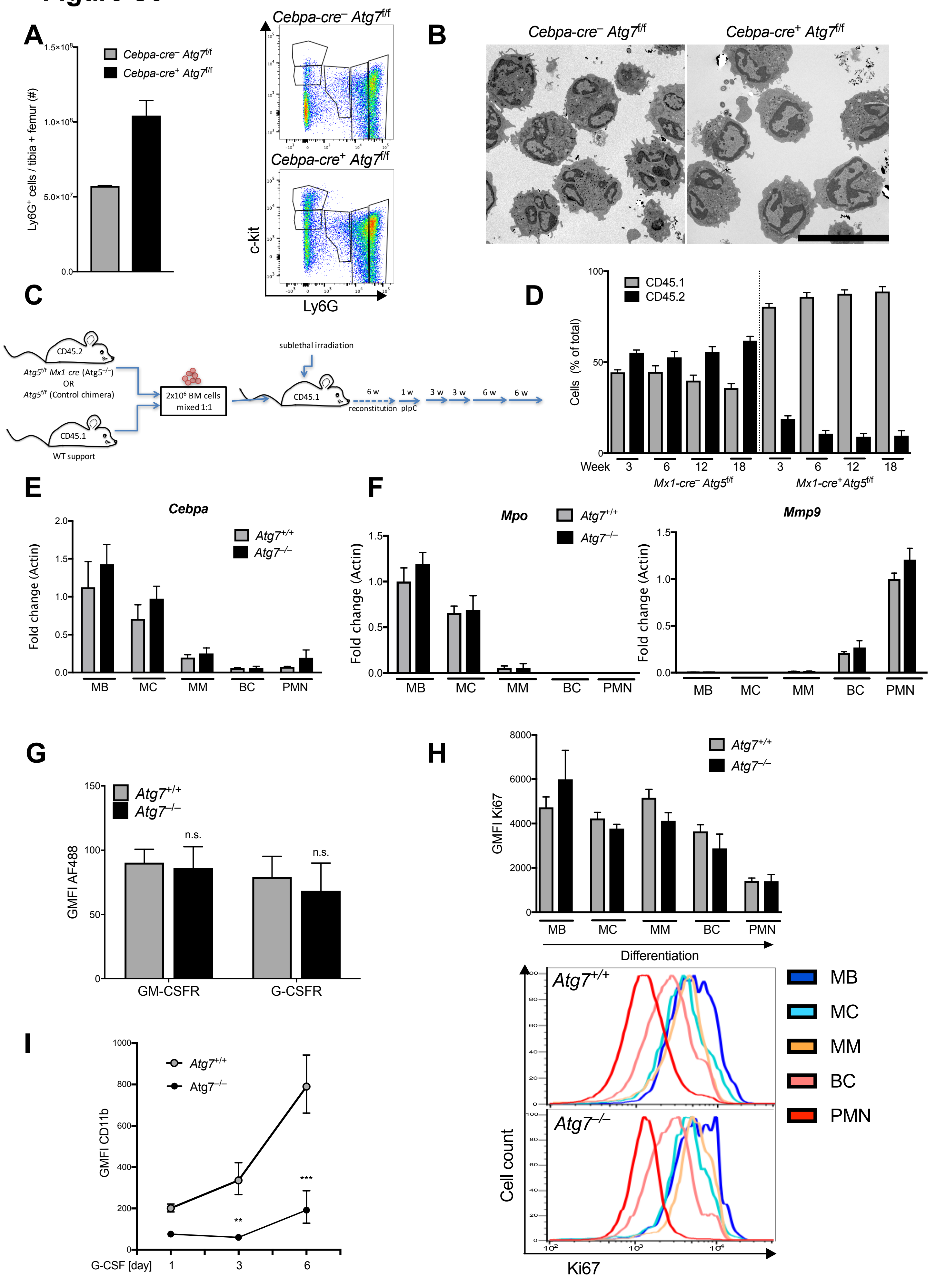

A
Cebpa-cre⁻ Atg7f/f
Cebpa-cre⁺ Atg7f/f
Ly6G⁺ cells / tibia + femur (#)
c-kit
Ly6G
B
Cebpa-cre⁻ Atg7f/f
Cebpa-cre⁺ Atg7f/f
C
CD45.2
Atg5f/f Mx1-cre (Atg5⁻/⁻)
OR
Atg5f/f (Control chimera)
CD45.1
WT support
2x10⁶ BM cells mixed 1:1
sublethal irradiation
CD45.1
6 w
reconstitution
1 w
pIpC
3 w
3 w
6 w
6 w
D
CD45.1
CD45.2
Cells (% of total)
Week
3
6
12
18
Mx1-cre⁻ Atg5f/f
Mx1-cre⁺Atg5f/f
E
Cebpa
Atg7+/+
Atg7⁻/⁻
Fold change (Actin)
MB
MC
MM
BC
PMN
F
Mpo
Mmp9
Atg7+/+
Atg7⁻/⁻
Fold change (Actin)
MB
MC
MM
BC
PMN
G
Atg7+/+
Atg7⁻/⁻
GMFI AF488
n.s.
n.s.
GM-CSFR
G-CSFR
H
Atg7+/+
Atg7⁻/⁻
GMFI Ki67
MB
MC
MM
BC
PMN
Differentiation
Atg7+/+
Atg7⁻/⁻
Cell count
Ki67
MB
MC
MM
BC
PMN
I
Atg7+/+
Atg7⁻/⁻
GMFI CD11b
**
***
G-CSF [day]
1
3
6

Figure S4

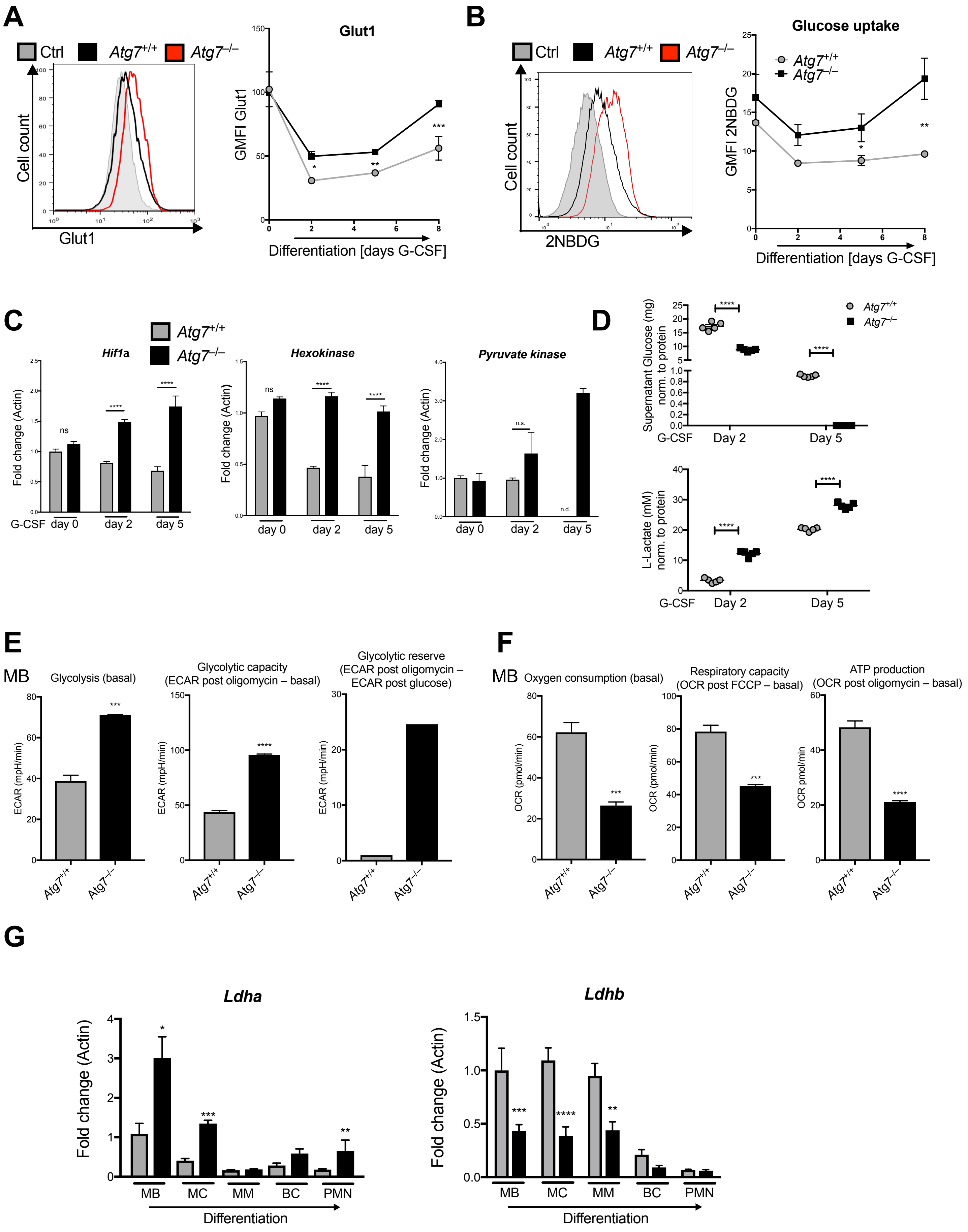

A
Ctrl
Atg7+/+
Atg7–/–
Cell count
Glut1
Glut1
GMFI Glut1
Differentiation [days G-CSF]
B
Ctrl
Atg7+/+
Atg7–/–
Cell count
2NBDG
Glucose uptake
Atg7+/+
Atg7–/–
GMFI 2NBDG
Differentiation [days G-CSF]
C
Atg7+/+
Atg7–/–
Hif1a
Hexokinase
Pyruvate kinase
Fold change (Actin)
G-CSF
day 0
day 2
day 5
ns
n.s.
n.d.
D
Atg7+/+
Atg7–/–
Supernatant Glucose (mg) norm. to protein
L-Lactate (mM) norm. to protein
G-CSF
Day 2
Day 5
E
MB
Glycolysis (basal)
Glycolytic capacity (ECAR post oligomycin – basal)
Glycolytic reserve (ECAR post oligomycin – ECAR post glucose)
ECAR (mpH/min)
Atg7+/+
Atg7–/–
F
MB
Oxygen consumption (basal)
Respiratory capacity (OCR post FCCP – basal)
ATP production (OCR post oligomycin – basal)
OCR (pmol/min)
OCR pmol/min
Atg7+/+
Atg7–/–
G
Ldha
Ldhb
Fold change (Actin)
MB
MC
MM
BC
PMN
Differentiation

Figure S5

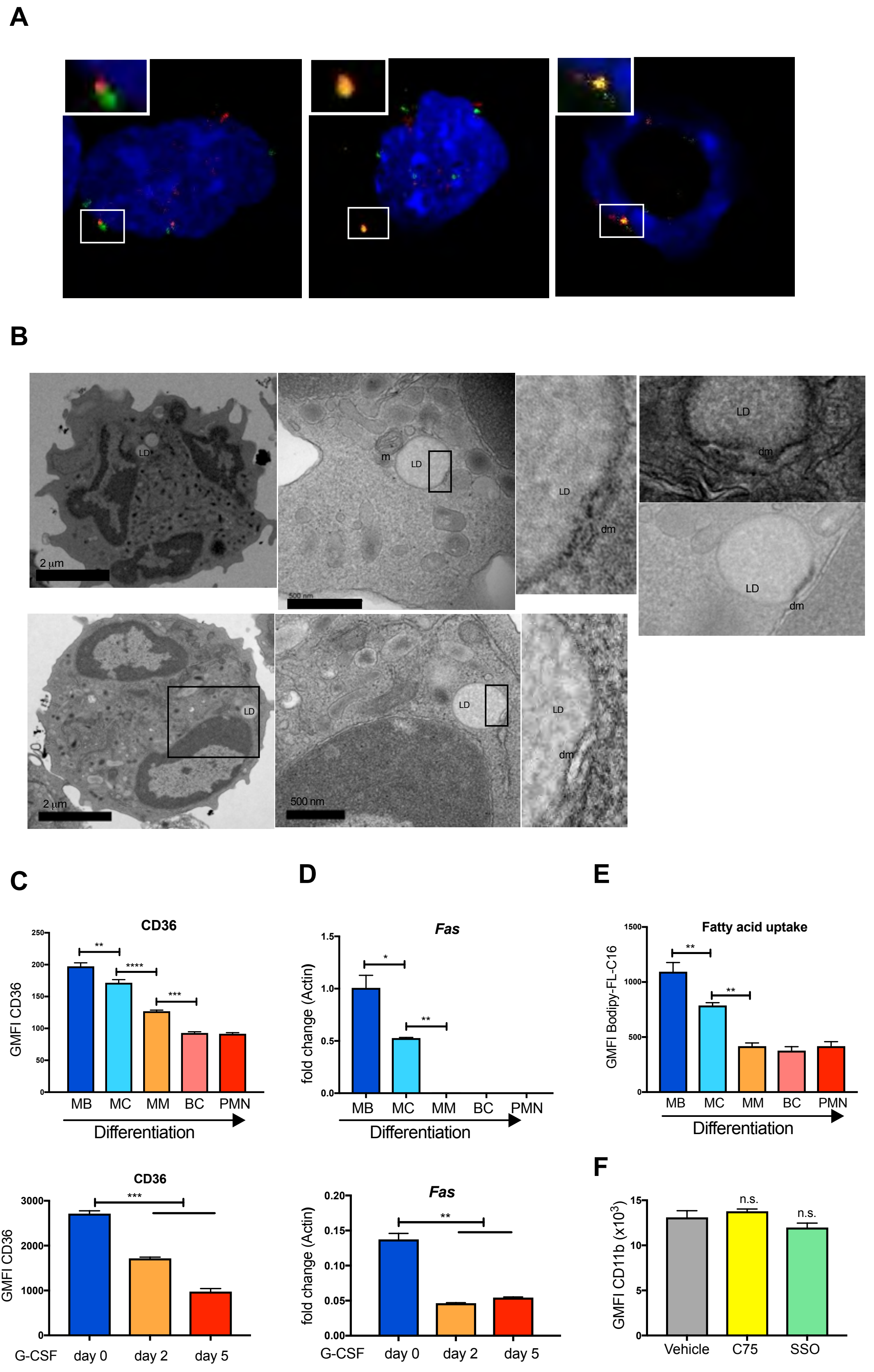
A
B
LD
m
dm
2 μm
500 nm
C
CD36
GMFI CD36
MB
MC
MM
BC
PMN
Differentiation
G-CSF
day 0
day 2
day 5
D
Fas
fold change (Actin)
E
Fatty acid uptake
GMFI Bodipy-FL-C16
F
GMFI CD11b (x10^3)
Vehicle
C75
SSO
n.s.

Figure S6

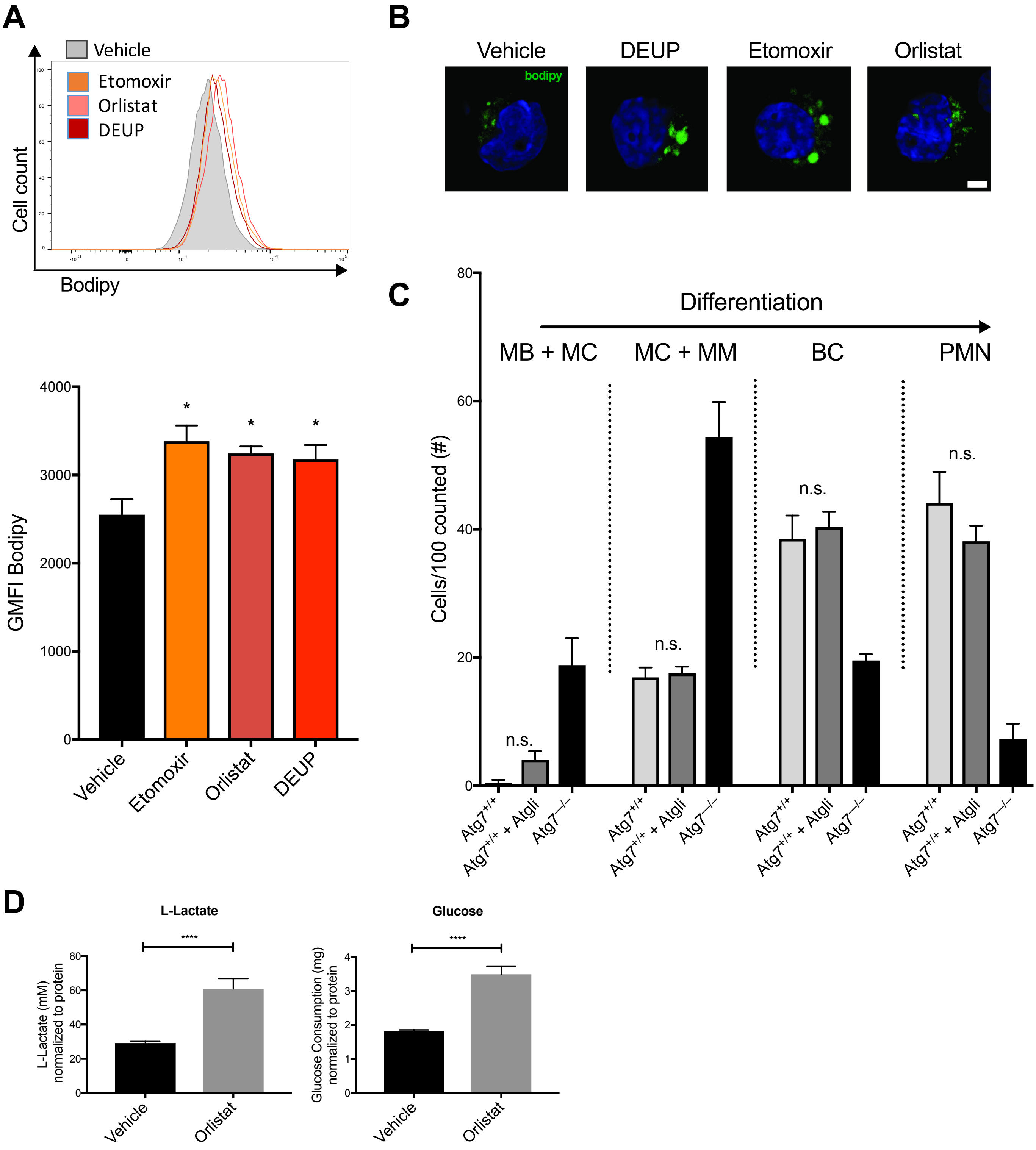
A
Vehicle
Etomoxir
Orlistat
DEUP
Cell count
Bodipy
GMFI Bodipy
B
Vehicle
DEUP
Etomoxir
Orlistat
bodipy
C
Differentiation
MB + MC
MC + MM
BC
PMN
n.s.
Cells/100 counted (#)
Atg7+/+
Atg7+/+ + Atgli
Atg7−/−
D
L-Lactate
L-Lactate (mM) normalized to protein
Glucose
Glucose Consumption (mg) normalized to protein
****
Vehicle
Orlistat

Figure S7

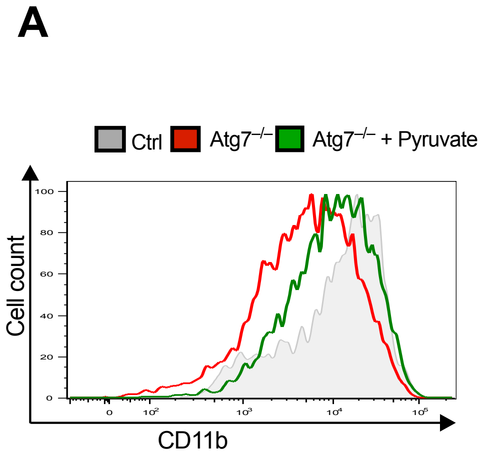
A
Ctrl
Atg7–/–
Atg7–/– + Pyruvate
Cell count
100
80
60
40
20
0
0
10²
10³
10⁴
10⁵
CD11b

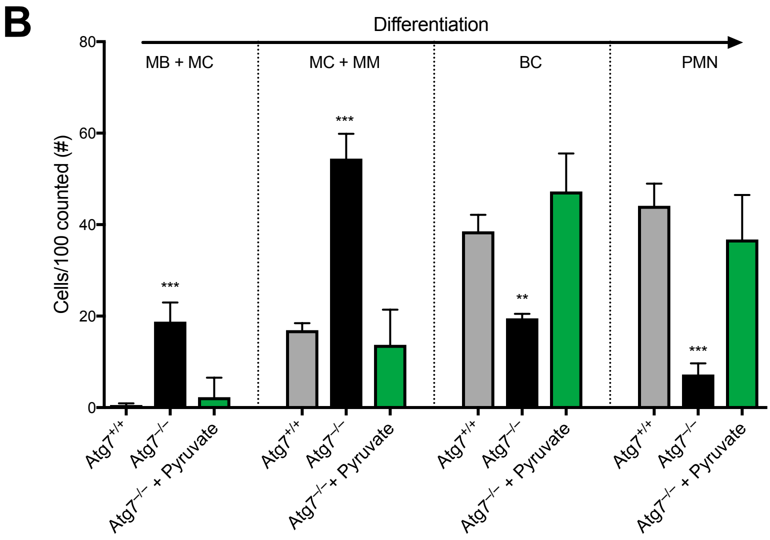
B
Differentiation
MB + MC
MC + MM
BC
PMN
Cells/100 counted (#)
80
60
40
20
0
***
***
**
***
Atg7+/+
Atg7–/–
Atg7–/– + Pyruvate
Atg7+/+
Atg7–/–
Atg7–/– + Pyruvate
Atg7+/+
Atg7–/–
Atg7–/– + Pyruvate
Atg7+/+
Atg7–/–
Atg7–/– + Pyruvate

	#1 MB, Myeloblast				#2 MC, Myelocyte				#3 MM, Metamyelocyte				#4 BC, Band cell				#5 PMN, Polymorphonuclear neutrophil			
**Gene name**	**Fold change (Actin) normalized to WT (ddCt)**	**SEM**	**p-value**	**Trend, significance**	**Fold change (Actin) normalized to WT (ddCt)**	**SEM**	**p-value**	**Trend, significance**	**Fold change (Actin) normalized to WT (ddCt)**	**SEM**	**p-value**	**Trend, significance**	**Fold change (Actin) normalized to WT (ddCt)**	**SEM**	**p-value**	**Trend, significance**	**Fold change (Actin) normalized to WT (ddCt)**	**SEM**	**p-value**	**Trend, significance**
Gpi1	2.073399278	0.283161894	0.0105	↑ *	1.816118651	0.375481673		↑	1.175608259	0.109539585		→	1.443392775	0.274038275		→	2.495438393	0.749486181		↑
Aldoa	3.609628316	0.111584904	0.0118	↑ *	4.411776932	1.391648427		↑	6.489416955	0.698907242		↑	2.065571554	0.622298718		↑	2.86803569	0.98502905		↑
Eno1	1.756484867	0.135989998		↑	2.082271675	0.164636881	0.0323	↑ *	3.810627023	1.575175279		↑	2.660757418	0.469373493		↑	1.228696223	0.169417275		→
Gapdh	1.399514604	0.072515204	0.0006	↑ ***	1.55370129	0.135766253	0.0024	↑ **	1.671204638	0.476902007		↑	1.384923963	0.140660851		→	1.252213077	0.03862611		→
Hif1a	1.902848332	0.242755913	0.0266	↑ *	1.542628921	0.162861848		↑	0.720169981	0.198562528		→	1.334738376	0.295744302		→	4.08330296	1.735873147		↑
Hk	2.188626205	0.098761607	0.0004	↑ ***	3.44599309	0.661468596	0.0075	↑ **	2.579311319	1.096219509		↑	1.333025928	0.069759527		→	1.369423513	0.09096196		→
Idi1	2.226968332	0.266257195		↑	1.95058024	0.596680115	0.036	↑ *	2.462855859	0.605709806	0.0298	↑ *	1.293596282	0.067110866		→	1.22281266	0.228020398		→
Ldh-a	1.867072149	0.191154741	0.0192	↑ *	2.163377481	0.232820498	<0.0001	↑ ****	1.722675878	0.435661039		↑	1.287491254	0.011897906		→	1.316770552	0.096778897		→
Pgm1	1.330845572	0.250064122		→	2.407006264	1.055989145		↑	0.974432715	0.196506941		→	1.251578728	0.239240382		→	1.214429471	0.200731166		→
Phgdh	2.523080414	0.292178362	0.0075	↑ **	1.952364371	0.136020071	0.0143	↑ *	1.033835216	0.197551989		→	1.239762606	0.156434494		→	1.700424016	0.370405524		↑
Pk	1.274671587	0.286521313	<0.0001	↑ ****	5.594022912	0.207903886	0.0011	↑ **	1.766923558	1.086311704		↑	5.531675067	0.078520555		↑	-	-		
Slc16a3	2.103559953	0.750917729		↑	3.308830242	1.405233901	0.0381	↑	1.49622697	0.15110416		→	4.134993992	1.486124866		↑	2.855671034	0.493491102	0.0301	↑ *
Slc2a1	1.228504701	0.392412635		→	2.936752528	1.65507189	0.023	↑	1.748850809	1.055313851	0.0076	↑ **	1.634300091	0.736335546	0.0048	↑ **	1.471360434	0.431790961		→
Pdk3	2.121277908	0.239228016	0.0027	↑ **	1.931352268	0.405773664		↑	1.320470108	0.242019293		→	1.537820472	0.289003191		↑	4.092738648	2.058286292		↑
Tpi1	2.213382799	0.193418355		↑	4.329457715	1.977087661	0.0452	↑	0.555276046	0.339254447		↓	1.941161689	0.495075627		↑	3.264354686	1.483787416		↑

Table S1 (related to Figure 4) Expression of glycolytic genes in BM derived neutrophil precursors from control and *Atg7*$^{-/-}$ mice.

Figure S1 Characterisation of neutrophil precursor stages (related to Figure 1)
A) Gating strategy for neutrophil precursor stages after gating on live single cells (Satake et al., 2012). Lineage cocktail includes CD4, CD5, CD8, CD19, B220, Ter119, antibodies. C-kit and CD34 are used to exclude CD34^{-} c-kit^{+} stem/progenitor cells and FSC/SSC gating excludes eosinophils.
B) Expression of *Atg7* and *Tfeb* in consecutive neutrophil differentiation stages. Data shown as fold change normalized to actin +/- SEM ($\Delta\Delta$Ct) (n=4 mice/group).
C) Cell surface CD11b expression as a marker to neutrophil differentiation in G-CSF stimulated 32D cells, as no monocyte/macrophage lineages develop in G-CSF condition (Valtieri et al., 1987). Surface CD11b was quantified as geometric MFI +/- SEM at the indicated days of G-CSF treatment (100 nM) from three experiments.
D) Wright-Giemsa stains showing representative morphological stages during G-CSF induced differentiation for all experiments with 32D myeloblasts. The corresponding morphological stages are indicated analogous to primary granulopoiesis.
E) Myeloblasts were cultured with 100 nM G-CSF for the indicated duration and subjected to LC3-II western blotting. The absence of LC3-I was confirmed by comparison to Jurkat lysate, representative of three experiments.

Figure S2 Mice conditionally deleted for *Atg7* accumulate abnormal neutrophils (related to Figure 2)
A) *Atg7* deletion was assessed at four distinct stages of neutrophil generation in control and *Vav-cre*$^{+}$ x *Atg7*$^{f/f}$ (left) or *Cebpa-cre*$^{+}$ x *Atg7*$^{f/f}$ (right) mouse bone marrow cells. Fold change +/- SEM was normalized to Actin and plotted relative to wildtype ($\Delta\Delta$Ct) (n=4 mice/group). HSC, hematopoietic stem cell; GMP, granulocyte-monocyte progenitor; MM, metamyelocyte; PMN, polymorphonuclear neutrophil.
B) Frequencies of Ly6G^{+} cells in indicated tissues as % of total live cells +/- SD (n=4 mice/group).
C) Cell surface expression of Ly6G maturation marker is plotted as MFI +/- SEM in total neutrophil lineage in control and *Atg7*$^{-/-}$ mice (*Vav-cre*$^{+}$ x *Atg7*$^{f/f}$) (n=4 mice/group)
D) Phagocytosis was measured within the mature PMN population with highest Ly6G expression, Ly6G^{high}, after 1 hour incubation with zymosan beads and 1 µg/ml LPS. Data was plotted as % of cells bead^{+} +/- SEM among the top 20% Ly6G^{high} population.
E) Wright-Giemsa stained cytospins (left) and morphological quantification of PMN and myelocytic precursor stages (right) among the mature Ly6G^{high} PMN population

(gating as indicated). Black arrows indicate myelocytes; orange arrows indicate droplets. Significance tested by Fisher´s exact test, *p<0.05.

Figure S3 *Cebpa-cre*$^{+}$ x *Atg7*$^{f/f}$, *Vav-cre*$^{+}$ x *Atg7*$^{f/f}$ and *Mx1-cre*$^{+}$ x *Atg5*$^{f/f}$ chimeric mice confirm a cell intrinsic neutrophil differentiation defect (related to Figure 3)

A) Absolute number of Ly6G^{+} cells in *Cebpa-cre*$^{–}$ x *Atg7*$^{f/f}$ and *Cebpa-cre*$^{+}$ x *Atg7*$^{f/f}$ mouse bone marrow (n=4 mice) and example images of precursor stage gating for all experiments involving precursor gating.

B) Representative electron micrographs of CD11b^{+} Ly6G^{+} BM cells from the indicated genotypes from two independent experiments.

C) Schematic of experimental set-up for mixed, inducible hematopoietic bone marrow chimeras. pIpC was injected i.p. (250 µg pIpC in 250 µL saline) e.o.d. for one week to induce excision.

D) Peripheral blood reconstitution with donor (CD45.2) and recipient (CD45.1) type cells shown as % of total cells +/- SD at the indicated weeks after pIpC-induced *Atg5* deletion.

E-F) Gene expression of the myeloid transcription factor *Cebpa* (E) and the early and late granule genes *Mpo* and *Mmp9* (F) in control and *Vav-cre*$^{+}$ x *Atg7*$^{f/f}$ mice (n=4 mice/group) after separation of the indicated precursor populations and normalization to *Actin* relative to control ($\Delta\Delta$Ct).

G) geometric MFI +/- SEM from control and *Vav-cre*$^{+}$ x *Atg7*$^{f/f}$ for the cell surface expression of G-CSF and GM-CSF receptors (n=4 mice/group).

H) Cell cycle exit was measured as geometric MFI of Ki67 expression (top) by intracellular flow cytometry in the indicated precursor populations of control (*Vav-cre*$^{–}$ x *Atg7*$^{f/f}$) and *Vav-cre*$^{+}$ x *Atg7*$^{f/f}$ mice (n=3 mice/group); Ki67 histograms are shown for each genotype (bottom).

I) CD11b maturation marker cell surface expression after culture of *Atg7*$^{+/+}$ and *Atg7*$^{–/–}$ myeloblasts for the indicated duration with G-CSF to induce differentiation (100 nM). Data are geometric MFI of CD11b +/- SEM, representative of four experiments. The same data is shown as histograms in Figure 3 H.

Figure S4 Excessive glycolysis and perturbed mitochondrial respiration without autophagy is conserved *in vitro* and *in vivo* (related to Figure 4)

A-E) CRISPR *Atg7*$^{+/+}$ and *Atg7*$^{–/–}$ myeloblasts (derived from 32D cells) were maintained in the presence of IL-3 (10 ng/µl) or cultured in G-CSF (100 nM) for the indicated duration.

A+B) Representative histogram (left) and quantification (right) of Glut-1 expression (A) and 2NBDG uptake (B) as indication of glycolytic activity. Tinted grey histograms are FMO= "fluorescence minus one" baseline FITC fluorescence control. Quantification on the right shows the geometric MFI +/- SEM in *Atg7*$^{+/+}$ and *Atg7*$^{–/–}$ cells at indicated timepoints. Data from three independent biological replicates from two experiments, *p < 0.05, **p < 0.01, ***p < 0.001.

C) Gene expression analysis of the indicated glycolytic genes in 200 myeloblasts during G-CSF induced differentiation. Data from four biological replicates measured in technical triplicates in one experiment, representative of two experiments. Fold change +/- SEM of biological replicates is shown normalized to actin relative to day 0 control (ΔΔCt), ****p < 0.0001.
D) Enzymatic quantification of glucose consumption (top) and lactate accumulation in the supernatant (bottom) after the indicated time of G-CSF induced neutrophil differentiation of 32D myeloblast precursors. Concentrations were determined based on a standard curve and normalized to protein content of each culture.
E) Quantification of glycolytic parameters from Figure 4H. Extracellular acidification rate is shown as mpH/min +/- SEM, data are from four biological replicates from two independent CRISPR *Atg7*$^{-/-}$ clones and *Atg7*$^{+/+}$ controls at the myeloblast stage cultured in IL-3. ***p < 0.001, ****p < 0.0001.
F) Quantification of mitochondrial metabolism assay from Figure 4I. Oxygen consumption rate (OCR) is shown as pmol/min +/- SEM, data are from four biological replicates from two independent CRISPR *Atg7*$^{-/-}$ clones and *Atg7*$^{+/+}$ control, ***p < 0.001, ****p < 0.0001.
G) Extracellular acidification rate is shown as mpH/min +/- SEM from 4-6 independent CRISPR *Atg7*$^{-/-}$ cultures and *Atg7*$^{+/+}$ controls after the indicated duration of G-CSF mediated differentiation. *p < 0.05, **p < 0.01.
H) Gene expression analysis of lactate dehydrogenase isoforms (*Ldha,* lactate generation*; Ldhb,* pyruvate generation). Five neutrophil precursor populations were sorted by flow-cytometry from *Atg7*$^{+/+}$ (*Vav-cre*$^{-}$ x *Atg7*$^{f/f}$) or *Atg7*$^{-/-}$ (*Vav-cre*$^{+}$ x *Atg7*$^{f/f}$) mice (100 cells/population, n=4-5 mice/group). Fold change +/- SEM of biological replicates is plotted normalized to actin relative to MB control (ΔΔCt). *p < 0.05, **p < 0.01, ***p < 0.001, ****p < 0.0001.

Figure S5 Lipophagy, lipid synthesis and -uptake during neutrophil differentiation (related to Figure 5)
A) Neutrophil precursors were purified from bone marrow of LC3-GFP transgenic mice by flow cytometry and stained with LD540 lipid droplet dye. Different representative stages of lipophagy were observed; left, direct contact with partial overlap of lipid droplet and LC3^{+} vesicle; middle, complete colocalizaiton of lipid and LC3 signal; right, dispersed lipid signal around double positive vesicle.
B) Neutrophil precursors were purified from bone marrow of wildtype mice by flow cytometry and prepared for electron microscopy to visualize the formation of membraneous structures around lipid droplets (See also (Singh et al., 2009).
C) Cell surface expression of the lipid uptake receptor CD36 in five consecutive neutrophil differentiation stages from wildtype mouse BM (n=3 mice/group) (top) and during 5 days of G-CSF induced neutrophil differentiation of 32D myeloblast precursors (bottom). Data shown as geometric MFI +/- SEM, significance tested by one-way Anova. **p < 0.01, ***p < 0.001, ****p < 0.0001.

D) Expression of fatty acid synthase (*Fasn*) in five consecutive neutrophil differentiation stages sorted by flow-cytometry from wildtype mouse BM (n=2 mice/group) (top) and during 5 days of G-CSF induced neutrophil differentiation of 32D myeloblast precursors (bottom). Data shown as fold change +/- SEM normalized to *Actin* relative to first stage ($\Delta\Delta$Ct), significance tested by one-way Anova. *p < 0.05, **p < 0.01.
E) Uptake of the labelled fatty acid Bodipy-FL-C16 in neutrophil differentiation stages from wildtype mouse BM (n=2 mice/group) 2 hours after i.v. injection of Bodipy-FL-C16 (25 mg/kg). Data shown as geometric MFI +/- SEM, significance tested by one-way Anova. **p < 0.01.
F) Cell surface expression of CD11b maturation marker after 5 days of G-CSF induced neutrophil differentiation of 32D myeloblast precursors in the presence of the specific *Fas*-inhibitor C75 or the CD36 inhibitor SSO. n.s., not significant. Data shown as geometric MFI +/- SEM, significance tested by student´s T-test.

Figure S6 Lysosomal, but not cytosolic lipases are essential for granulopoiesis (related to Figure 6)
A) Bodipy histograms (top) and quantification as geometric MFI +/- SEM (bottom) after the indicated drug treatments for five days during G-CSF induced differentiation confirms the accumulation of lipid droplets (See also Figure 6C). Representative of three independent experiments.
B) Confocal micrographs of bodipy$^+$ lipid droplet accumulation in response to the indicated drug treatments (See also supplemental methods). Scale bar, 5 µm.
C) Wild type myeloblasts were differentiated in the presence or absence of the cytosolic lipase inhibitor atglistatin (Atgli) with G-CSF for 7 days. *Atg7*$^{-/-}$ for comparison. Data shown as number of cells in each morphological group per 100 cells counted +/- SEM. n.s., non-significant.
D) Enzymatic quantification of lactate accumulation in the supernatant (left) and glucose consumption (right) after 5 days of G-CSF induced neutrophil differentiation of 32D myeloblast precursors in the presence of the lipase inhibitor orlistat. Concentrations were determined based on a standard curve and normalized to protein content of each culture.

Figure S7 Pyruvate rescues differentiation in *Atg7*$^{-/-}$ granulopoiesis (related to Figure 7)
A) Representative histograms of CD11b expression for three experiments after 5 days G-CSF (100 nM) shows rescue of CD11b expression in *Atg7*$^{-/-}$ cells in the presence of pyruvate (green).
B) Histological quantification of indicated differentiation stages shows a near complete rescue of neutrophil differentiation block in *Atg7*$^{-/-}$ cells in the presence of pyruvate (2 mM, green). Data shows number of cells/100 cells counted per group, from four biological replicates representative of two independent clones from one experiment, representative of three experiments.
