# Supplementary material for: Autophagy-Dependent Generation of Free Fatty Acids Is Critical for Normal Neutrophil Differentiation
Source: Immunity. 2017 Sep 19;47(3):466–480.e5. doi: 10.1016/j.immuni.2017.08.005 (PMC5610174; doi:10.1016/j.immuni.2017.08.005)
Supplement: Document S2. Article plus Supplemental Information [file mmc2.pdf]

Immunity

Article

Autophagy-Dependent Generation of Free Fatty Acids Is Critical for Normal Neutrophil Differentiation

Graphical Abstract

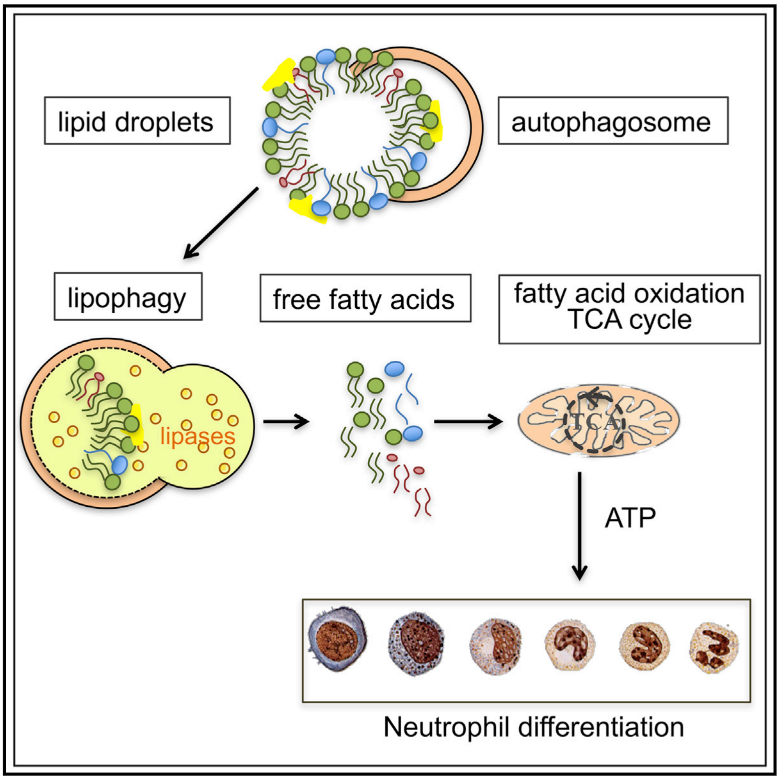

Highlights

- Autophagy is critical for neutrophil differentiation *in vivo*
- Differentiating neutrophils shift from glycolysis to fatty acid oxidation
- By degrading lipid droplets, autophagy provides fatty acids, enabling this shift
- Fatty acids restore energy metabolism and differentiation in *Atg7*$^{-/-}$ granulopoiesis

Authors

Thomas Riffelmacher,
Alexander Clarke, Felix C. Richter, ...,
Holm Uhlig, Sten Eirik W. Jacobsen,
Anna Katharina Simon

Correspondence

katja.simon@ndm.ox.ac.uk

In Brief

Rapid neutrophil differentiation is key to the immune response against invading bacteria. Here, Riffelmacher et al. show that autophagy, the main recycling process in the cell, provides energy for neutrophil differentiation by degrading lipid droplets. This opens possible novel therapeutic avenues for the differentiation treatment of granulocytic leukemia.

Riffelmacher et al., 2017, Immunity *47*, 466–480
September 19, 2017 © 2017 The Authors. Published by Elsevier Inc.
http://dx.doi.org/10.1016/j.immuni.2017.08.005

CellPress

Immunity
Article

Autophagy-Dependent Generation of Free Fatty Acids Is Critical for Normal Neutrophil Differentiation

Thomas Riffelmacher,[1,2] Alexander Clarke,[1] Felix C. Richter,[1] Amanda Stranks,[2] Sumeet Pandey,[6] Sara Danielli,[2] Philip Hublitz,[3] Zhanru Yu,[4] Errin Johnson,[5] Tobias Schwerd,[6] James McCullagh,[7] Holm Uhlig,[6] Sten Eirik W. Jacobsen,[3,8,9,10,11] and Anna Katharina Simon[1,2,11,12,*]

[1]Kennedy Institute of Rheumatology, University of Oxford, Roosevelt Drive, Oxford OX3 7FY, UK
[2]MRC Human Immunology Unit, Weatherall Institute of Molecular Medicine, University of Oxford, John Radcliffe Hospital, Headington, Oxford OX3 9DS, UK
[3]MRC Molecular Hematology Unit, Weatherall Institute of Molecular Medicine, University of Oxford, John Radcliffe Hospital, Headington, Oxford OX3 9DS, UK
[4]Target Discovery Institute, Nuffield Department of Medicine, University of Oxford, Roosevelt Drive, Oxford OX3 7FZ, UK
[5]The Dunn School of Pathology, South Parks Road, Oxford OX1 3RE, UK
[6]Translational Gastroenterology Unit, Experimental Medicine, University of Oxford, John Radcliffe Hospital, Oxford OX3 9DU, UK
[7]Chemistry Research Laboratory, Department of Chemistry, University of Oxford, Mansfield Road, Oxford OX1 3TA, UK
[8]Department of Medicine Huddinge, Center for Hematology and Regenerative Medicine, Karolinska Institutet, Stockholm, Sweden
[9]Department of Cell and Molecular Biology, Wallenberg Institute for Regenerative Medicine, Karolinska Institutet, Stockholm, Sweden
[10]Karolinska University Hospital, Stockholm, Sweden
[11]Senior author
[12]Lead Contact
*Correspondence: katja.simon@ndm.ox.ac.uk
http://dx.doi.org/10.1016/j.immuni.2017.08.005

SUMMARY

Neutrophils are critical and short-lived mediators of innate immunity that require constant replenishment. Their differentiation in the bone marrow requires extensive cytoplasmic and nuclear remodeling, but the processes governing these energy-consuming changes are unknown. While previous studies show that autophagy is required for differentiation of other blood cell lineages, its function during granulopoiesis has remained elusive. Here, we have shown that metabolism and autophagy are developmentally programmed and essential for neutrophil differentiation *in vivo*. Atg7-deficient neutrophil precursors had increased glycolytic activity but impaired mitochondrial respiration, decreased ATP production, and accumulated lipid droplets. Inhibiting autophagy-mediated lipid degradation or fatty acid oxidation alone was sufficient to cause defective differentiation, while administration of fatty acids or pyruvate for mitochondrial respiration rescued differentiation in autophagy-deficient neutrophil precursors. Together, we show that autophagy-mediated lipolysis provides free fatty acids to support a mitochondrial respiration pathway essential to neutrophil differentiation.

INTRODUCTION

Neutrophils are the most abundant and short-lived human immune cells and are rapidly recruited to sites of infection as a first line of defense. Recently, they have attracted new attention with unexpected findings on their heterogeneity and plasticity (Nauseef and Borregaard, 2014) and their ability to survive long term and shape adaptive immune responses (Nicolás-Ávila et al., 2017). With about 1 × 10^6 neutrophils produced per second in the bone marrow (BM) of humans, granulopoiesis requires exquisite regulation. Multipotent progenitors (MPPs) give rise to granulocyte-monocyte progenitors (GMPs) and further to myeloblast cells (MBs), which are the first committed granulocyte precursors (Borregaard, 2010). MBs proceed through promyelocyte (MC), metamyelocyte (MM), and band cell (BC) stages to become a mature polymorphonuclear neutrophil (PMN) (Bardoel et al., 2014). This process is characterized by striking remodeling of the nucleus and temporally regulated granule generation by timed synthesis, which clearly demarcates stages of differentiation.

Recent advances have described the transcriptional networks that govern neutrophil differentiation in response to the granulocyte-colony stimulating factor (G-CSF) cytokine, but the mechanisms that enact these signals to enable differentiation remain poorly understood (Bugl et al., 2012).

Autophagy is a cellular recycling pathway that delivers diverse cytoplasmic material into double membraned autophagosome vesicles for lysosomal fusion and cargo degradation. This can free up essential metabolites for anabolic processes such as metabolic remodeling during differentiation (Mochida et al., 2015). Canonical macroautophagy (hereafter referred to as autophagy) has been demonstrated to be essential for terminal differentiation of several blood cell lineages (Riffelmacher and Simon, 2017). For example, the metabolic adaptation and maintenance of terminal CD8$^+$ memory and regulatory T cells both require autophagy (Kabat et al., 2016; Puleston et al., 2014; Wei et al., 2016; Xu et al., 2014). While activated T cells preferentially engage glycolytic metabolism, memory and regulatory T cells switch to mitochondrial respiration for the bulk of ATP production (Buck et al., 2016; Zeng et al., 2013) and support this metabolic reprogramming by lipid breakdown and fatty acid oxidation (FAO) to supply acetyl-CoA metabolites for oxidative phosphorylation

 © 2017 The Authors. Published by Elsevier Inc.
This is an open access article under the CC BY license (http://creativecommons.org/licenses/by/4.0/).

(OXPHOS) (O'Sullivan et al., 2014; Pearce et al., 2009). However, whether autophagy affects metabolic fuel choice either during hematopoietic progenitor or T cell differentiation remains unknown. The mitochondrial quality control through selective degradation of mitochondria (mitophagy) also contributes to metabolic adaptation during differentiation as was shown in innate NK lymphocytes (O'Sullivan et al., 2015; Salio et al., 2014).

Autophagy-deficient neutrophils fail to mediate adequate inflammatory responses due to reduced degranulation, bacterial killing, and neutrophil extracellular trap (NET) formation (Bhattacharya et al., 2015; Remijsen et al., 2011), while another study has found no functional neutrophil defects (Rožman et al., 2015). However, the role of autophagy in neutrophil differentiation is not understood.

Many acute myeloblastic leukemias (AMLs) are characterized by an accumulation of large numbers of granulocyte precursors, which engage aerobic glycolysis (Warburg metabolism), due to impaired differentiation (Jiang and Nakada, 2016; Latif and Holyoake, 2016). Defective autophagy has been associated with myelodysplastic syndrome (MDS), its progression to AML, and excessive glycolysis (Watson et al., 2015). Moreover, several drugs used to treat AML-affected patients also induce autophagy (Auberger and Puissant, 2017). Therefore, understanding how autophagy affects normal neutrophil differentiation may have direct relevance to myeloid malignancies and potential therapeutic interventions.

Here, we show that autophagy is essential for neutrophil differentiation in a cell-intrinsic manner, *in vitro* as well as *in vivo*. We observed extensive metabolic reprogramming during normal differentiation, limiting glycolytic activity while engaging mitochondrial respiration and mobilizing intracellular lipid stores. Autophagy-deficient neutrophil precursors were unable to shift toward mitochondrial respiration and displayed excessive glycolysis, lipid droplet accumulation, and ATP depletion. Notably, inhibition of lysosomal lipolysis or fatty acid oxidation within mitochondria alone was sufficient to cause defective neutrophil differentiation. Importantly, administration of free fatty acids (FFAs) or pyruvate for mitochondrial respiration rescued differentiation in autophagy-deficient neutrophil precursors and restored normal glucose metabolism. Taken together, we show that autophagy-mediated lipid breakdown provides FFAs to support the FAO-OXPHOS pathway for ATP production, which is essential for neutrophil differentiation.

RESULTS

Dynamic Regulation of Autophagy Occurs during Early Neutrophil Differentiation

To understand the regulation of autophagy in normal neutrophil differentiation, we defined the five differentiation stages of myeloblasts (MBs), myelocytes (MCs), metamyelocytes (MMs), band cells (BCs), and neutrophils (PMNs) (Satake et al., 2012) and confirmed those stages by morphology on Wright-Giemsa-stained cytospins (Figures 1A, 1B, and S1A), as well as by expression of relevant and stage-specific granule genes (Figure 1C). The expression of *Mpo*, a primary granule gene, is predominant in the early stages, whereas *Mmp9*, a tertiary granule gene, is expressed only at the band cell stage and later.

To measure stage-specific autophagy activity *in vivo*, we took advantage of *Map1lc3b*-GFP transgenic mice (Mizushima et al., 2004). When autophagosomal formation is induced, the small cytoplasmic protein Map1lc3b (hereafter referred to as LC3) is lipidated to LC3-II, which is incorporated into the autophagosomal membrane and routinely used to measure autophagy. Upon delivery of the autophagosomal content to the lysosome, LC3-II is degraded together with the cargo. If lysosomal degradation of LC3-II is inhibited by bafilomycin A1 (Baf), the build-up of LC3-II quantifies autophagic flux (Klionsky et al., 2016). We found robust autophagic flux at the MB and MC stages, which decreased at the MM and BC stages and slightly increased again in mature PMNs (Figure 1D). Consistent with this, we found the expression of *Tfeb* (the master regulator of lysosomal and autophagy gene expression [Settembre et al., 2011]) as well as the essential autophagy gene *Atg7* to follow the same dynamic expression pattern (Figure S1B). Furthermore, we found the highest numbers of LC3-II puncta by confocal microscopy in MB and MC stages as well as in mature PMNs (Figures 1E and 1F). Lastly, we measured LC3-II by immunoblot in the well-established IL-3-dependent culture system 32D that mimics myeloblast to neutrophil differentiation *in vitro* in response to G-CSF (Valtieri et al., 1987). In this model, uniform neutrophil differentiation could be followed over 5–7 days by expression of the maturation marker CD11b and nuclear morphology (Figures S1C and S1D). As expected, we found the same pattern of dynamic regulation of autophagy—particularly at early differentiation stages—as we observed in primary cells (Figure S1E). Whereas the higher autophagy activity in the mature PMN stage is likely important in neutrophil function (Bhattacharya et al., 2015), we hypothesized that autophagy in the early precursors may be relevant to the remodeling that occurs during differentiation.

Defective Autophagy Causes Accumulation of Immature Neutrophils and Neutrophil Dysfunction

To study the role of autophagy in early granulopoiesis *in vivo*, we used mice with a conditional deletion of the essential autophagy machinery component *Atg7*, under control of the pan-hematopoietic *Vav*-gene promotor (*Vav-cre* × *Atg7*$^{f/f}$), resulting in deletion of *Atg7* at the hematopoietic stem and progenitor level (Mortensen et al., 2011a). We observed a profound ablation of *Atg7* expression (>80%) by qPCR in mature neutrophils, their myeloblast precursors, and at the level of hematopoietic stem cells (HSCs) (Figure S2A) as well as absence of Atg7 protein in Ly6G^{+} cells (Figure 3F). While neutrophils appeared expanded in *Vav-cre* × *Atg7*$^{f/f}$ mice (Figure 2A), they expressed lower amounts of Ly6G (Figure 2B and quantified in Figure S2C). Total Ly6G-expressing cells in the BM were expanded 2-fold and also significantly increased in spleen, blood, and peritoneum (Figures 2C and S2B). We also observed a significant reduction in the phagocytic capacity of *Atg7*$^{-/-}$ total Ly6G^{+} cells in response to LPS (Figure 2D). Moreover, and despite reduced phagocytosis, intracellular survival of *S. aureus* was significantly increased, indicating that autophagy-deficient neutrophils are also functionally defective (Figure 2E).

To address to what degree *Atg7*$^{-/-}$ neutrophils were compromised in their differentiation, we further assessed their differentiation status. *Atg7*$^{-/-}$ total Ly6G^{+} BM cells had increased *Mpo* and reduced *Mmp9* expression, a granule gene signature

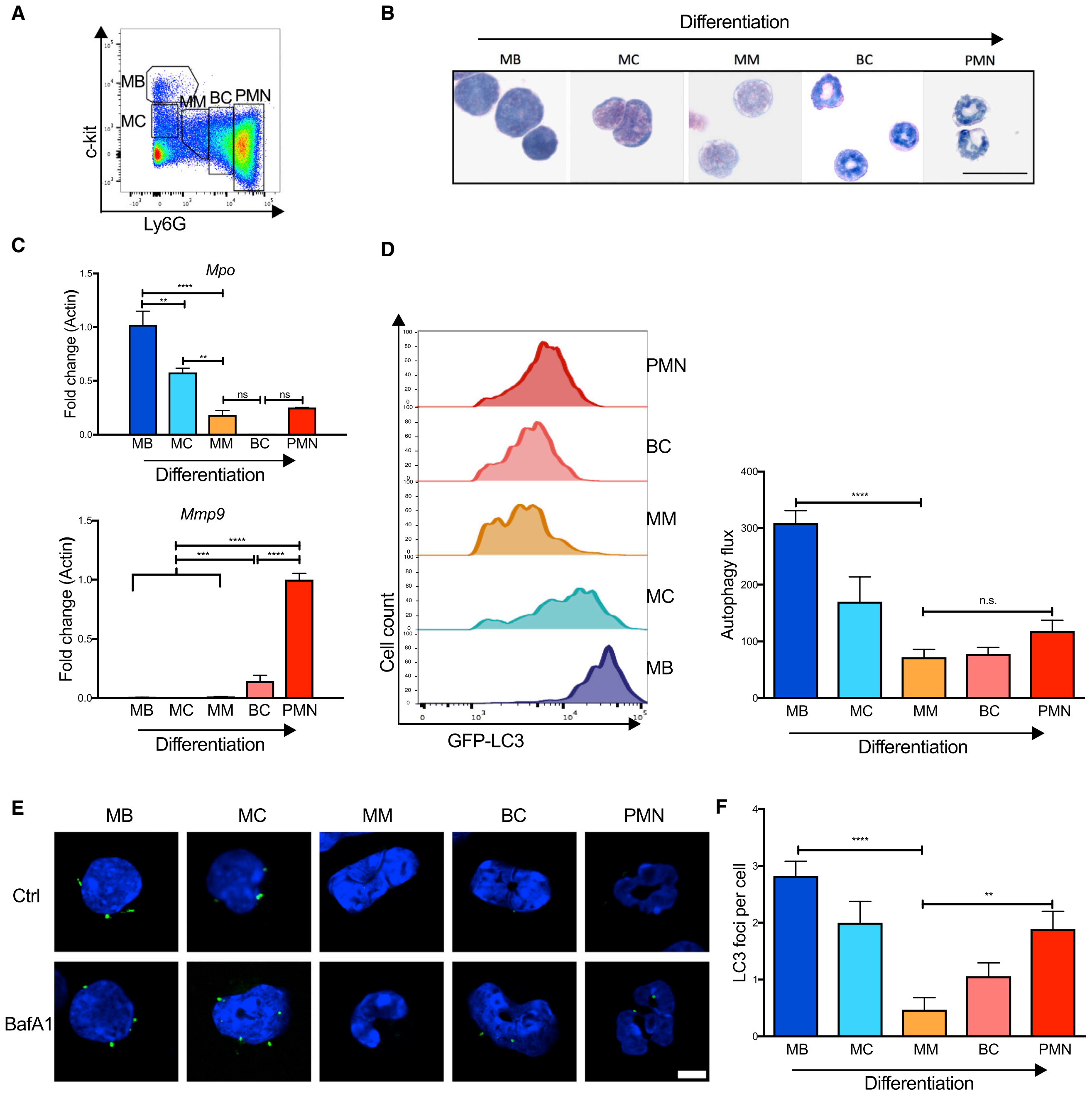

Figure 1. Autophagy during Normal Granulopoiesis

BM neutrophil precursor populations from wild-type or *Map1lc3b*-GFP mice were separated by flow cytometry and analyzed for gene expression, morphology, and autophagy activity.

(A) Gating strategy used to enrich populations of MBs, MCs, MMs, BCs, and PMNs as used in Figures 1, 3, and 4. MB, myeloblast; MC, myelocyte; MM, metamyelocyte; BC, band cell; PMN, polymorphonuclear neutrophil.

(B) Representative Wright-Giemsa-stained cytospins from two experiments of sorted precursor stages for morphological validation. Scale bar, 25 μm.

(C) Transcriptional expression of relevant primary (*Mpo*) and tertiary (*Mmp9*) granule genes relative to *Actin*. Data are shown as mean ± SEM (ΔΔCt) (n = 5–6 mice/group), representative of three experiments.

(D) Representative histograms (left) of Map1lc3b-GFP fluorescence in flow cytometry-sorted neutrophil precursor populations from *Map1lc3b*-GFP transgenic mice. Autophagy flux (right) was measured by flow cytometry after treatment with vehicle or bafilomycin A1 (10 nM, 2 hr) calculated as (MFI (Bafilomycin A1–Basal) /MFI Basal) in two independent experiments.

(E and F) Confocal microscopy of Map1lc3b-GFP fluorescence in flow cytometry-sorted precursors after treatment with vehicle or bafilomycin A1 (10 nM, 2 hr). Representative images (E), scale bar 5 μm, and quantification of LC3-II foci per cell (F).

Data are shown as mean ± SEM (n = 3 mice) from three independent experiments. For all panels *p < 0.05, **p < 0.01, ***p < 0.001, ****p < 0.0001. See also Figure S1.

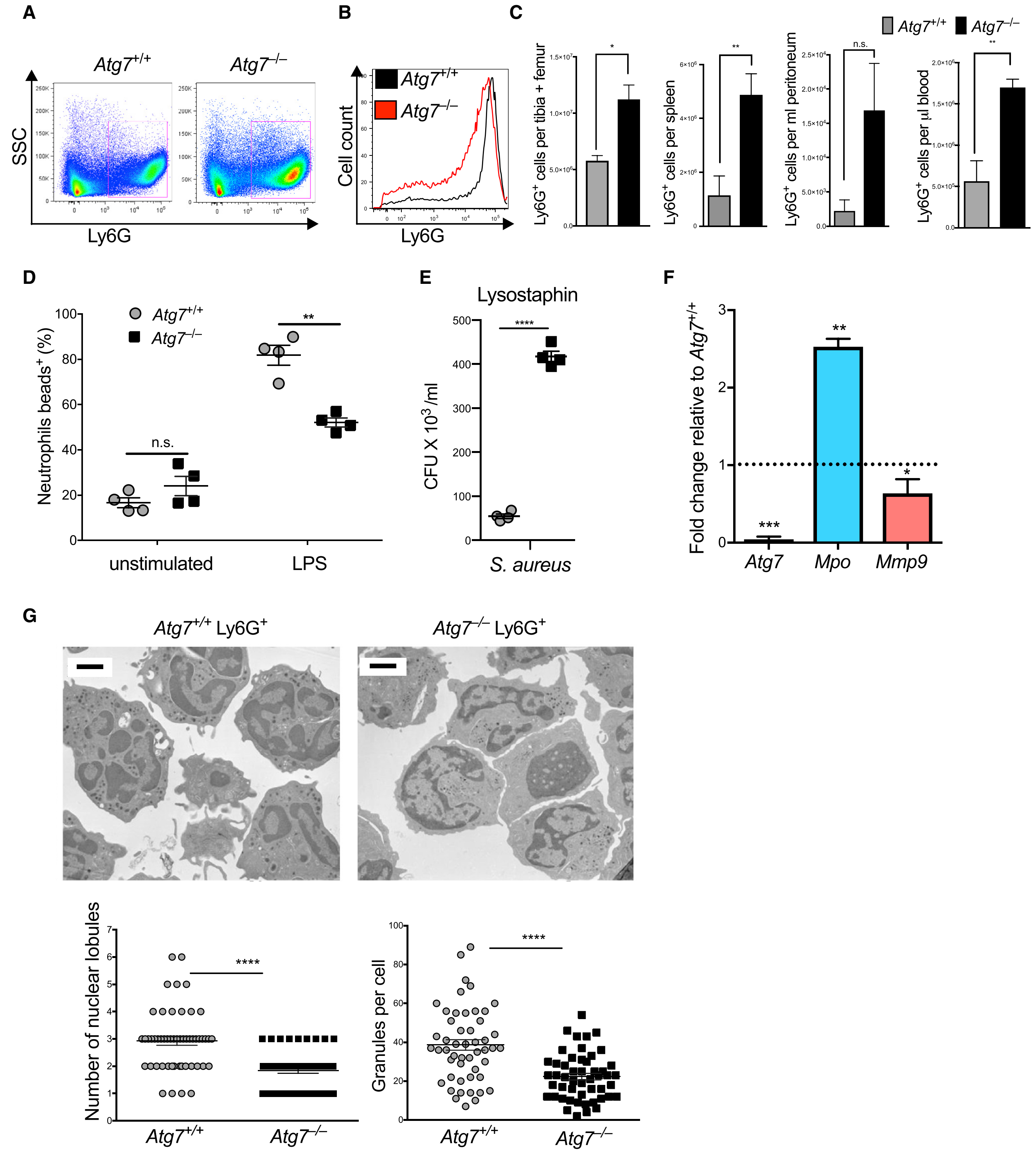

Figure 2. Acquisition of Terminally Differentiated Neutrophil Morphology and Function Requires Autophagy

Phenotype of control and *Vav-cre*+ × *Atg7*f/f (*Atg7*−/−) BM neutrophil populations.

(A) Representative flow cytometry plots of Ly6G+SSChi BM neutrophil population from four independent experiments.

(B) Representative histogram of Ly6G expression in BM from four independent experiments.

(C) Absolute numbers of Ly6G+ cells in different tissues. Data are shown as mean ± SEM (n = 4–5 mice/group), representative of three experiments.

(D) Phagocytosis was quantified as number of Ly6G+ cells that took up fluorescent latex beads during a 30 min culture period in the presence or absence of LPS. Data are shown as mean ± SEM (n = 4 mice/group), representative of three experiments.

(legend continued on next page)

consistent with incomplete neutrophil differentiation (Figure 2F). In agreement, electron microscopy revealed a distinct reduction in electron-dense granules as well as reduced nuclear lobularization (Figure 2G). Notably, this morphological and functional dysregulation was also observed when only the Ly6G^{hi} fraction was specifically isolated by flow cytometry (Figures S2D and S2E). Thus, early *in vivo* deletion of the autophagy gene *Atg7* leads to accumulation of a phenotypically and functionally immature population of neutrophils.

Autophagy Is Essential for *In Vivo* Neutrophil Differentiation in a Cell-Intrinsic Manner

In order to conditionally delete *Atg7* specifically at the earliest neutrophil precursor stage but not in multipotent HSCs, we next generated mice with *Cebpa-cre*-driven excision of *Atg7*. *Cebpa* is reported to be expressed predominantly at the GMP stage, whereas there is negligible expression in HSCs or lymphoid lineages (Wölfler et al., 2010). We confirmed by qPCR that while Atg7 mRNA was present in HSCs, it was profoundly reduced (>90%) in GMP, MM, and PMN populations in *Cebpa-cre* × *Atg7*$^{f/f}$ mice (Figure S2A). Complete absence of Atg7 protein was also confirmed by immunoblotting in total Ly6G^{+} BM cells (Figure 3A). As was seen in *Vav-cre* × *Atg7*$^{f/f}$ mice, the Ly6G-expressing population was expanded (Figure S3A) and retained a "left-shift" in morphology (Figures 3B and S3B) and granule gene expression (Figure 3C), resembling increased immature myeloblasts and myelocyte precursors.

While this suggests that the observed differentiation defect occurs in the earliest committed neutrophil precursors (MBs-MCs) as a result of autophagy deficiency, it remained possible that the Atg7-specific phenotype might be unrelated to autophagy. Second, defective differentiation may induce feedback granulopoiesis, through either intrinsic or extrinsic mechanisms, leading to secondary accumulation of neutrophil precursors. To address both issues, we generated mixed bone marrow chimeric mice with inducible deletion of *Atg5*, another essential autophagy gene (Hara et al., 2006), under control of the *Mx1-cre* recombinase (*Mx1-cre* × *Atg5*$^{f/f}$; *Atg5*$^{-/-}$). Inducible deletion in the adult mouse after BM chimera generation also excludes potential effects of autophagy deletion during development or homing to the BM. Granulopoiesis was assessed after pIpC-induced deletion of *Atg5* by serial bleeding over 18 weeks in both *Atg5*$^{-/-}$ and control BM chimera (experimental design shown in Figure S3C). In mixed *Atg5*$^{-/-}$ chimeras, the wild-type competitor cells gradually out-competed Atg5-deficient cells over time, as expected from the fundamental role of autophagy in self renewal of HSCs (Figure S3D; Mortensen et al., 2011a). However, while the abundance of total Ly6G^{+} cells remained constant within CD45.2^{+} cells in control chimera, a significant increase in CD45.2^{+} (and not CD45.1^{+}) Ly6G^{+} cells was observed over time in *Atg5*$^{-/-}$ chimeras (Figure 3D). To assess their differentiation status, we isolated CD45.1^{+} and CD45.2^{+} CD11b^{+}Ly6G^{+} cells by flow cytometry. Immature neutrophil precursors were dominant among the CD45.2^{+} but not among the CD45.1^{+} cells of the *Atg5*$^{-/-}$ chimera, nor in any of the sorted populations of the control chimeric mice (Figure 3E). Together, our findings demonstrate that the differentiation block in autophagy-deficient neutrophils occurs at or after the GMP stage, is cell intrinsic, is not Atg7 specific, and occurs independent of the role of autophagy during development.

We next tested whether autophagy deficiency has an impact on the regulation of cytokine receptors, transcription factors, or granule genes that are key for neutrophil differentiation. Cebpa is the main transcription factor that acts at the GMP and MB stages to induce granulopoiesis and granule gene expression via G-CSF-receptor-mediated signaling (Iida et al., 2008; Zhang et al., 1997). While *Cebpa* expression was found highest at the MB and MC stages and then decreased over differentiation, it was not affected in *Atg7*$^{-/-}$ animals (Figure S3E). Similarly, *Atg7* deletion did not affect granule gene transcription within isolated stages of neutrophil differentiation, and the cell surface expression of G-CSF and GM-CSF receptors was also unchanged in *Atg7*$^{-/-}$ neutrophils (Figures S3F and S3G). This confirms that the described differentiation defect is independent of Cebpa-dependent transcriptional events and that our finding of an immature gene signature among the total Ly6G^{+} population reflects a left shift toward immature stages. Lastly, we tested whether cell cycle exit was affected by the loss of autophagy as previously reported in other cell types (Lee et al., 2012). Whereas the gradual cell cycle exit upon terminal neutrophil differentiation was confirmed by Ki67 flow cytometry, we found no impact on this in autophagy-deficient neutrophils (Figure S3H).

To further pursue mechanistic studies and as myeloblasts and myelocytes are rare cell populations *in vivo*, we generated *Atg7*$^{-/-}$ clones via CRIPSR/Cas9-mediated gene editing of the *in vitro* myeloblast line 32D (as in Figure S1D). We targeted independent regions within the early exons 3 and 4 by specific sgRNAs. All independent clones showed complete absence of Atg7 protein as well as loss of autophagic flux, shown by lack of LC3-I to LC3-II conversion (Figure 3F). While LC3 is typically detectable only as lipidated LC3-II in 32D cells during homeostasis even when autophagy is low (as in Figure S1E), absence of Atg7-mediated lipidation led to artificial LC3-I accumulation. Notably, in the presence of G-CSF, *Atg7*$^{+/+}$ control myeloblasts upregulated the maturation marker CD11b normally and uniformly formed mature neutrophils within 5–7 days in the presence of G-CSF. In contrast, *Atg7*$^{-/-}$ clones exclusively retained immature morphology with a lack of lobulated nuclei based on Wright-Giemsa-stained cytospins (Figure 3G) and had severely reduced CD11b expression (Figures 3H and S3I). This *in vitro*

(E) Bacterial killing with Ly6G^{+} BM cells. Extracellular bacteria were removed by lysostaphin treatment (100 μg/mL) and live intracellular bacteria which escaped killing were measured as CFU × 10^{3}/mL on agar plates. Data are shown as mean ± SEM (n = 3 mice/group). *S. aureus*, *Staphylococcus aureus*.

(F) Gene expression was measured in isolated *Atg7*$^{-/-}$ and *Atg7*$^{+/+}$ Ly6G^{+} cells, *Mpo*, myeloperoxidase; *Mmp9*, matrix-metalloprotease 9. Data are shown as mean ± SEM (ΔΔCt) (n = 3 mice/group), representative of two experiments.

(G) Electron micrographs were prepared to visualize *Atg7*$^{+/+}$ and *Atg7*$^{-/-}$ CD11b^{+} Ly6G^{+} BM cells (left), and number of nuclear lobes/section and granules were quantified (right). Scale bar, 2 μm.

*p < 0.05, **p < 0.01, ***p < 0.001, ****p < 0.0001. See also Figure S2.

system therefore confirms that the effect is a cell-intrinsic neutrophil differentiation defect as a consequence of autophagy deficiency at the committed neutrophil precursor stage.

Autophagy Is Essential for the Energy-Metabolic Reprogramming during Neutrophil Differentiation

Autophagy has been suggested to be important for metabolic homeostasis in T cells (Puleston et al., 2014; Xu et al., 2014), but the metabolic requirements during granulopoiesis are not known. We therefore first characterized the metabolic changes occurring during normal neutrophil differentiation by metabolomic analysis. We observed profound metabolic reprogramming during G-CSF-induced differentiation *in vitro*, as illustrated in the principal component analysis (PCA) of 4,215 metabolites (Figure 4A). In addition, *in vivo* transcriptional analysis of major metabolic enzymes within consecutive differentiation stages using the Fluidigm Biomark platform showed unsupervised clustering of individual granulopoietic stages, indicating a programmed metabolic remodeling (Figure 4B and Table S1). While all 15 glycolytic pathway genes analyzed were downregulated during *in vivo* granulopoiesis (Figure 4B), the mitochondrial content simultaneously underwent a distinct 2-fold increase (Figure 4C). These results are consistent with a metabolic shift during granulopoiesis that limits glycolysis but engages mitochondrial respiration.

Autophagy has been shown to regulate the balance of energy-generating metabolic pathways (Liu and Czaja, 2013; Galluzzi et al., 2014; Guo et al., 2016; Watson et al., 2015). We therefore investigated the impact of Atg7 deficiency on these metabolic pathways. During G-CSF-induced differentiation, PCA showed a clear overall difference in the metabolome of *Atg7*$^{+/+}$ and *Atg7*$^{-/-}$ cells (Figure 4D). Of note, seven of the ten glycolysis intermediates were detectable by mass spectrometry, all of which were increased in *Atg7*$^{-/-}$ myeloblasts compared to *Atg7*$^{+/+}$ controls (Figure 4E).

We then extended this *ex vivo* finding to gene expression analysis of the glycolysis pathway in the five consecutive stages of neutrophil differentiation in *Atg7*$^{-/-}$ BM neutrophil precursor populations. All differentiation stages showed elevated expression of a panel of 15 glycolysis genes in Atg7-deficient mice (Figure 4F and Table S1). We next quantified the uptake of the fluorescent glucose analog 2NBDG and found *Atg7*$^{-/-}$ precursors to have significantly increased uptake, particularly in the early MB and MC stages (Figure 4G).

Similarly, cultured control myeloblast clones showed decreasing expression of the glucose transporter Glut-1 and uptake of 2NBDG over the course of G-CSF-induced differentiation, while *Atg7*$^{-/-}$ myeloblasts progressively increased both of these parameters during differentiation (Figures S4A and S4B). The expression of key glycolytic genes also followed this trend (Figure S4C) in *Atg7*$^{-/-}$ cells. Glucose consumption and lactate production measured in the medium behaved similarly, with lactate production increased in *Atg7*$^{-/-}$ myeloblasts both at early (day 2) and late (day 5) differentiation stages (Figure S4D). We also quantified the extracellular acidification rate (ECAR) and found basal glycolysis, maximal glycolytic capacity (after oligomycin treatment), and spare glycolytic capacity (Δafter oligomycin treatment) to be increased in *Atg7*$^{-/-}$ myeloblasts (Figures 4H and S4E). In contrast, quantification of oxygen consumption rate (OCR) showed a decrease in basal respiration, spare respiratory capacity (Δafter FCCP treatment), and mitochondrial ATP production (Δafter oligomycin treatment) in *Atg7*$^{-/-}$ myeloblasts (Figures 4I and S4F). Notably, this was confirmed in *ex vivo* Ly6G^{+} *Atg7*$^{-/-}$ neutrophils showing increased basal ECAR and decreased OCR (Figure 4J) with significantly reduced ATP (Figure 4K). The compensatory glycolysis during *Atg7*$^{-/-}$ neutrophil differentiation was further confirmed by the increased expression of *Ldha* and decreased expression of *Ldhb* in early precursors (Figure S4G), switching from pyruvate use in the TCA cycle to generating lactate and NAD^{+} to keep glycolysis going. Thus, the metabolic switch that occurs during granulopoiesis is cell intrinsic, conserved *in vivo* and *in vitro*, and depends on autophagy to limit glycolysis but rather engage mitochondrial respiration.

Autophagy Degrades Lipid Droplets during Neutrophil Differentiation

We hypothesized that, as glycolysis decreases during neutrophil differentiation, breakdown of lipids for FAO might take over to support mitochondrial respiration and ATP production. Autophagy was recently identified as a major pathway for lipid breakdown in hepatocytes by targeting lipids into autophagosomes for hydrolyzation via lysosomal lipases (lipophagy), yielding FFAs (Singh et al., 2009). Indeed, we found a depletion of free fatty acids, in particular palmitic, oleic, and eicosanaic acids by lipdomics in *Atg7*$^{-/-}$ myeloblasts (Figure 5A). In addition, we found accumulation of vesicles in the Wright-Giemsa cytospins from *in vitro* cultured *Atg7*$^{-/-}$ neutrophils after G-CSF-induced differentiation (Figure 5B, top), which were confirmed to be of lipid origin upon staining with the neutral lipid-specific dye Bodipy (Figure 5B, bottom). In parallel we used Bodipy to label lipid droplets for quantitative flow cytometry on different days after addition of G-CSF and confirmed this with Nile Red, another well-established marker for lipid droplets. Lipid droplet amount decreased during *Atg7*$^{+/+}$ myeloblast differentiation *in vitro*, while *Atg7*$^{-/-}$ neutrophils progressively accumulated lipids (Figures 5C and 5D). Increased number of lipid droplets per cell was also confirmed in *ex vivo* sorted Ly6G^{+} *Atg7*$^{-/-}$ neutrophils from BM by fluorescent microscopy (Figure 5E). Similarly, *Atg5*$^{-/-}$ neutrophils from *Cebpa-cre* × *Atg5*$^{f/f}$ mice showed increased lipid droplets by fluorescent microscopy (Figure 5F). Bodipy staining by flow cytometry confirmed that lipid droplets decreased slightly during *Atg7*$^{+/+}$ neutrophil differentiation *in vivo* while they accumulated progressively in *Atg7*$^{-/-}$ neutrophils over their differentiation (Figure 5G). Finally, we observed colocalization of autophagosomes with lipid droplets in sorted BM progenitors by confocal microscopy (Figure S5A) and observed the formation of membrane structures with more than one layer around lipid droplets on electron micrographs (Figure S5B) as previously described (Singh et al., 2009). We conclude that degradation of lipid droplets during normal neutrophil differentiation is mediated by autophagy to maintain the pool of cytosolic FFAs while preventing lipid accumulation.

Notably, all stages of neutrophil differentiation, in particular the earliest stages with highest autophagy, expressed the cell surface FFA transporter CD36, demonstrated active uptake of FFAs, and also expressed the gene encoding fatty acid

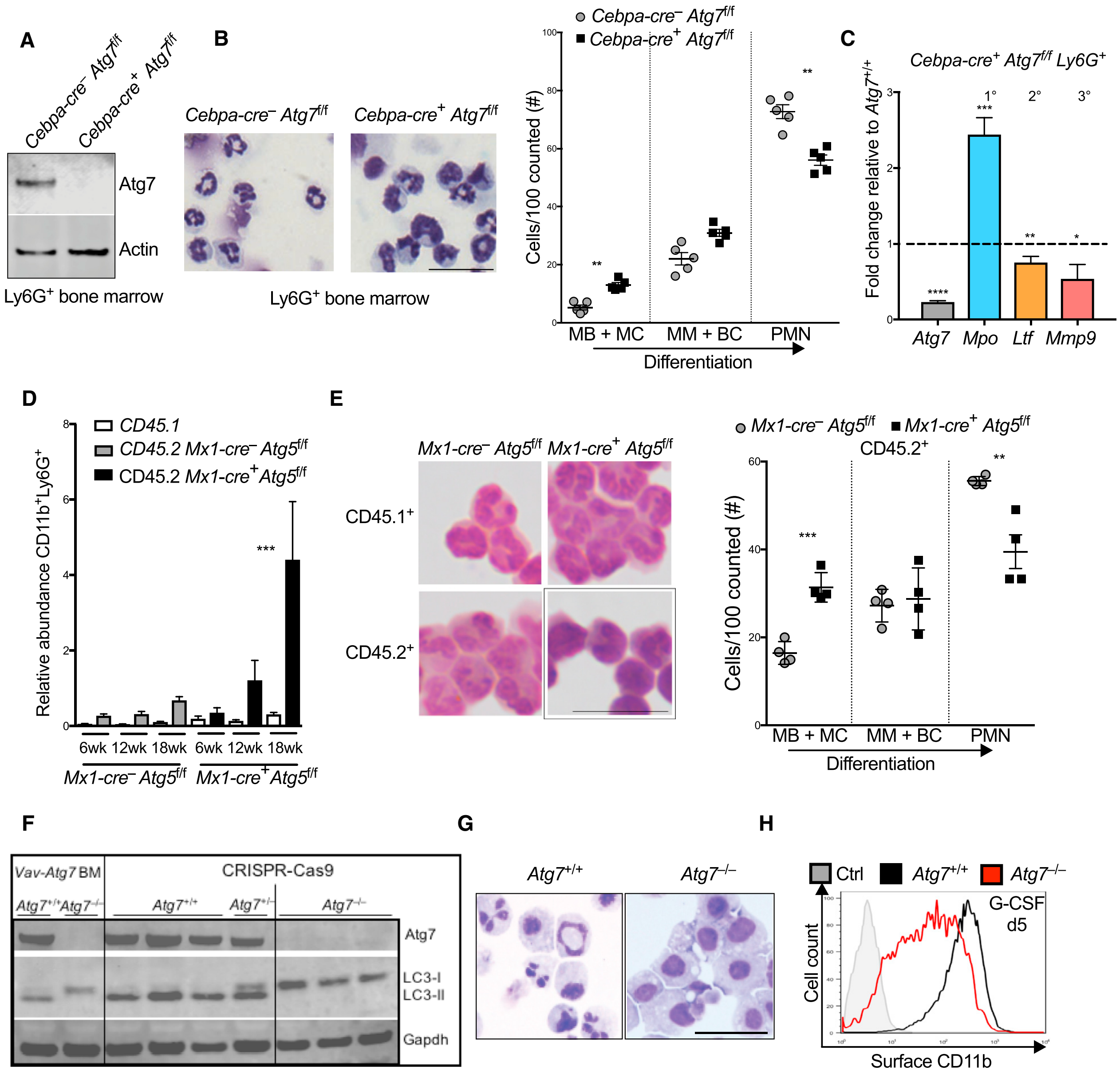

Figure 3. Cell-Intrinsic, Atg5- and Atg7-Dependent Autophagy Is Essential for Neutrophil Differentiation

(A) Immunoblot from Ly6G^{+} BM cells isolated from control (*Cebpa-cre*$^{-}$) or *Cebpa-cre*$^{+}$ × *Atg7*$^{f/f}$ mice. Actin is used as loading control.

(B) Representative images of Wright-Giemsa-stained cytospins of Ly6G^{+} cells (left) and histological quantification (right) of indicated maturation stages by morphology from n = 4–5 mice/group. Representative of two independent experiments. Scale bar, 25 μm.

(C) mRNA expression of indicated genes in *Cebpa-cre*$^{+}$ × *Atg7*$^{f/f}$ Ly6G^{+} BM cells relative to control (*Cebpa-cre*$^{-}$) Ly6G^{+} cells. *Mpo*, myeloperoxidase, primary granule; *Ltf*, lactoferrin, secondary granule; *Mmp9*, matrix-metalloprotease 9, tertiary granule. Data are shown as mean ± SEM (ΔΔCt) (n = 3–5 mice/group), representative of two experiments.

(D) Abundance of cells expressing CD11b^{+}Ly6G^{+} at surface relative to total lymphocytes (T + B cells, gated as CD3^{+} and CD19^{+}, respectively) in PB of control (*Mx1-cre*$^{-}$ × *Atg5*$^{f/f}$) and *Mx1-cre*$^{+}$ × *Atg5*$^{f/f}$ chimeric mice at indicated time points. Data are shown as mean ± SEM (n = 6 mice/group), representative of two experiments. ***$p < 0.001$, tested by two-way ANOVA.

(E) Wright-Giemsa-stained cytospins of Ly6G^{+} cells (left; scale bar, 25 μm) and histological quantification (right; per 100 total Ly6G^{+} cells) of indicated maturation stages by morphology after flow cytometric separation of CD45.2^{+} Ly6G^{+} cells, representative of two experiments.

(F) Immunnoblot for Atg7 and LC3-I+II protein expression. Ly6G^{+} BM cells from *Vav-cre*$^{+}$ × *Atg7*$^{f/f}$ mice were used as control. Shown are three independent clones of wild-type (*Atg7*$^{+/+}$) and CRSIPR-Cas9-generated *Atg7*$^{-/-}$ genotypes grown clonally from sorted single cells.

(G) Wright-Giemsa-stained cytospins of *Atg7*$^{+/+}$ and *Atg7*$^{-/-}$ cells after 5 days culture in G-CSF (100 nM) representative of three independent experiments. Scale bar, 50 μm.

(H) Surface CD11b expression on *Atg7*$^{+/+}$ and *Atg7*$^{-/-}$ cells after 5 days in G-CSF-supplemented cultures. Unstained control cells from day 0 shown in gray. Representative of four independent experiments from three clones.

*$p < 0.05$, **$p < 0.01$, ***$p < 0.001$, ****$p < 0.0001$. See also Figure S3.

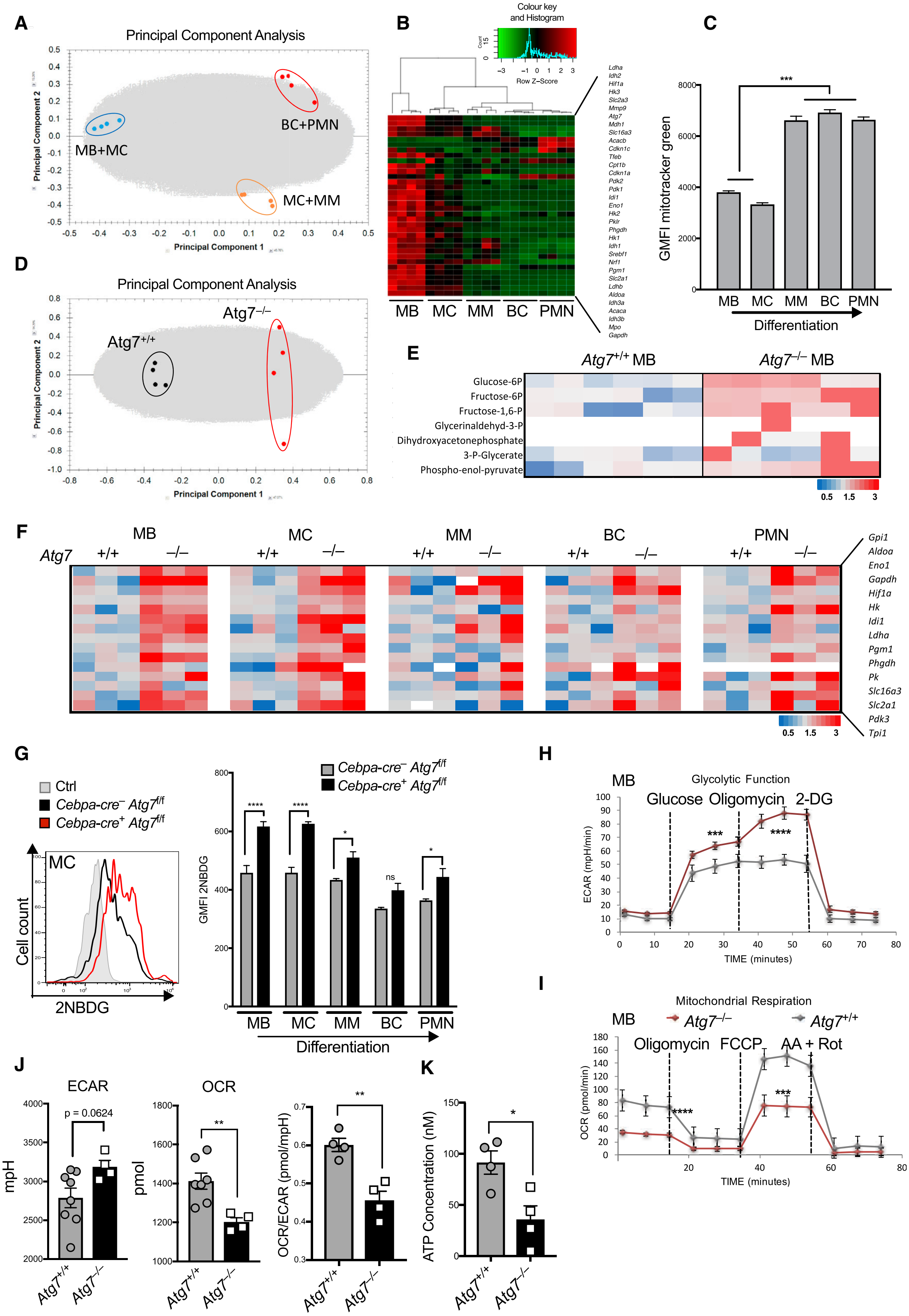

(legend on next page)

synthase (*Fasn*) (Figures S5C–S5E). Yet, total lipid content decreased during granulopoiesis, confirming active lipolysis. Pharmacological inhibition of FFA synthesis by C75 or the CD36 inhibitor SSO (Coort et al., 2002; Kuhajda et al., 2000), however, did not inhibit neutrophil maturation (Figure S5F), suggesting that neutrophils may have redundant pathways to maintain their lipid reserves.

Lipophagy Is Required for Neutrophil Metabolic Adaptation and Differentiation

We next addressed whether the defect in supply of FFAs for FAO and OXPHOS could be causative for the metabolic rewiring and the neutrophil differentiation defect in Atg7-deficient mice. We first tested whether defective lipolysis is sufficient to block neutrophil differentiation, by differentiating myeloblasts with G-CSF in the presence of the pan-lipase inhibitor DEUP, the lysosomal lipase (LAL) inhibitor orlistat, or the FAO inhibitor etomoxir. As expected, all three drugs led to an increase in lipid droplets (Figures S6A and S6B). Both lipase inhibitors DEUP and orlistat also increased glycolysis as measured by 2NBDG uptake (Figure 6A), lactate production, and glucose consumption (Figure S6D), supporting our finding that defective lipolysis signals for a glycolytic shift in metabolism when autophagy is disrupted. In further support of a causative role for this metabolic shift in disrupting differentiation of autophagy-deficient neutrophils, all three drugs led to a partial but significant inhibition of differentiation as seen by a decrease in the maturation marker CD11b (Figure 6B) and an increase in myeloblast-like morphology (Figure 6C). Lastly, wild-type 32D myeloblast differentiation was not affected by the cytosolic Atgl-lipase inhibitor atglistatin (Figure S6C). Taken together, both active lysosomal lipolysis and their subsequent oxidation in mitochondria are required for normal neutrophil differentiation and to prevent excessive glycolysis.

Free Fatty Acids and Pyruvate Are Sufficient to Restore Differentiation in Autophagy-Deficient Neutrophil Precursors

To confirm whether FFAs provided by lipophagy are necessary for normal neutrophil differentiation and metabolic homeostasis, we attempted to rescue differentiation of *Atg7*$^{-/-}$ neutrophil precursors by providing exogenous FFAs, known to be broken down for FAO. While linoleic acid (LA) has been shown to improve mature neutrophil function (Rodrigues et al., 2010), there have been no data implicating it in neutrophil differentiation. We treated *Atg7*$^{+/+}$ and CRISPR-*Atg7*$^{-/-}$ myeloblasts with LA or a mixture of saturated and unsaturated FFAs during G-CSF-induced differentiation. Both conditions led to an almost complete resolution of the increased glucose uptake (Figure 7A) and of the defective neutrophil differentiation of *Atg7*$^{-/-}$ myeloblasts (Figures 7B–7D). To determine whether fatty acids are used during neutrophil differentiation as energy substrates for FAO and OXPHOS, we also tested whether pyruvate, an energy metabolite that can substitute fatty acid-derived acetyl-CoA to directly fuel OXPHOS, also can rescue *Atg7*$^{-/-}$ differentiation. Pyruvate treatment alone was sufficient to restore differentiation of *Atg7*$^{-/-}$ myeloblasts as measured by CD11b expression (Figure S7A) and nuclear morphology (Figures 7C and S7B).

Taken together, administration of free fatty acids is sufficient to restore normal glucose metabolism and rescue differentiation in autophagy-deficient neutrophil precursors. The OXPHOS-fueling substrate pyruvate similarly restores differentiation, supporting that lipophagy provides FFAs for mitochondrial ATP generation in order to drive normal neutrophil differentiation.

Figure 4. Autophagy Is Required to Maintain Metabolic Balance during Granulopoiesis

(A, D, and E) Metabolites were extracted from 5 × 10^6 cells of the indicated genotypes and metabolomic data were acquired by ICxMS/MS. Data from one experiment with six biological replicates.

(A) Principal component analysis of myeloblasts after 2 and 5 days of G-CSF-induced differentiation (100 nM), scored based on the 4,215 detected metabolites (with Hotellings T2 ellipse at 0.95).

(B) Microfluidic gene expression analysis with the Fluidigm-Biomark array for the indicated metabolic genes (100 cells/population) in five neutrophil differentiation stages sorted by flow cytometry from wild-type mouse BM. Unsupervised clustering of replicates based on z-score (n = 4 mice).

(C) Mitochondrial content was quantified by flow cytometry from wild-type mouse BM as mitotracker green fluorescence intensity in indicated neutrophil precursor populations. Data are shown as mean ± SEM (n = 3 mice). ***$p < 0.001$, tested by one-way ANOVA.

(D) Principal component analysis of *Atg7*$^{+/+}$ myeloblasts and independent CRISPR *Atg7*$^{-/-}$ clones, scored based on the 4,215 detected metabolites with %CV < 30 on day 2 of G-CSF-induced differentiation.

(E) Glycolysis metabolite abundance for control and CRISPR *Atg7*$^{-/-}$ clones at the myeloblast stage cultured in IL-3. Heatmap shown as fold change normalized to wild-type abundance. Data represent six biological replicates per group from two independent clones. Seven of the ten glycolytic intermediates were detectable by mass spectrometry and are presented here.

(F) Gene expression profiling of the glycolytic pathway was done using the Fluidigm Biomark 48 × 48 dynamic array on 100 purified precursor cells of each of the indicated differentiation stages purified by flow cytometry from *Atg7*$^{-/-}$ and control *Atg7*$^{+/+}$ BM. Data from three separate mice per genotype. Heatmaps are shown as fold change relative to *Actin* and normalized to *Atg7*$^{+/+}$. All 15 glycolytic genes, except isoforms, were selected from the same gene panel as used in Figure 4B. Representative of three experiments.

(G) Uptake of fluorescent glucose (2NBDG) during control and *Cebpa-cre*$^{+}$ × *Atg7*$^{f/f}$ neutrophil differentiation. Representative histogram (left) shows myelocyte (MC) population. Quantification (right) as geometric MFI ± SEM (n = 3 mice/group). Data from three individual experiments. *$p < 0.05$, ****$p < 0.0001$, tested by two-way ANOVA.

(H and I) Oxygen consumption rate (OCR) (H) and extracellular acidification rate (ECAR) (I) was measured under basal conditions and in response to indicated drugs. Data represent mean of four biological replicates from two independent CRISPR *Atg7*$^{-/-}$ clones and *Atg7*$^{+/+}$ controls at myeloblast stage cultured in IL-3.

(J) Glycolysis and OXPHOS rates measured over 80 min as extracellular acidification (ECAR) and oxygen consumption (OCR), respectively, from *Atg7*$^{-/-}$ and control *Atg7*$^{+/+}$ freshly isolated Ly6G^{+} BM neutrophils. The ratio of OCR/ECAR was measured every 20 min ± SEM (n = 2 mice/group). Data representative of two experiments.

(K) Cellular ATP was measured with a luciferase-assay from 1 × 10^6 freshly isolated Ly6G^{+} BM neutrophils from control and *Atg7*$^{-/-}$ mice (*Cebpa-cre*$^{+}$ × *Atg7*$^{f/f}$, n = 4 mice/group) and concentrations determined based on a standard curve.

See also Figure S4.

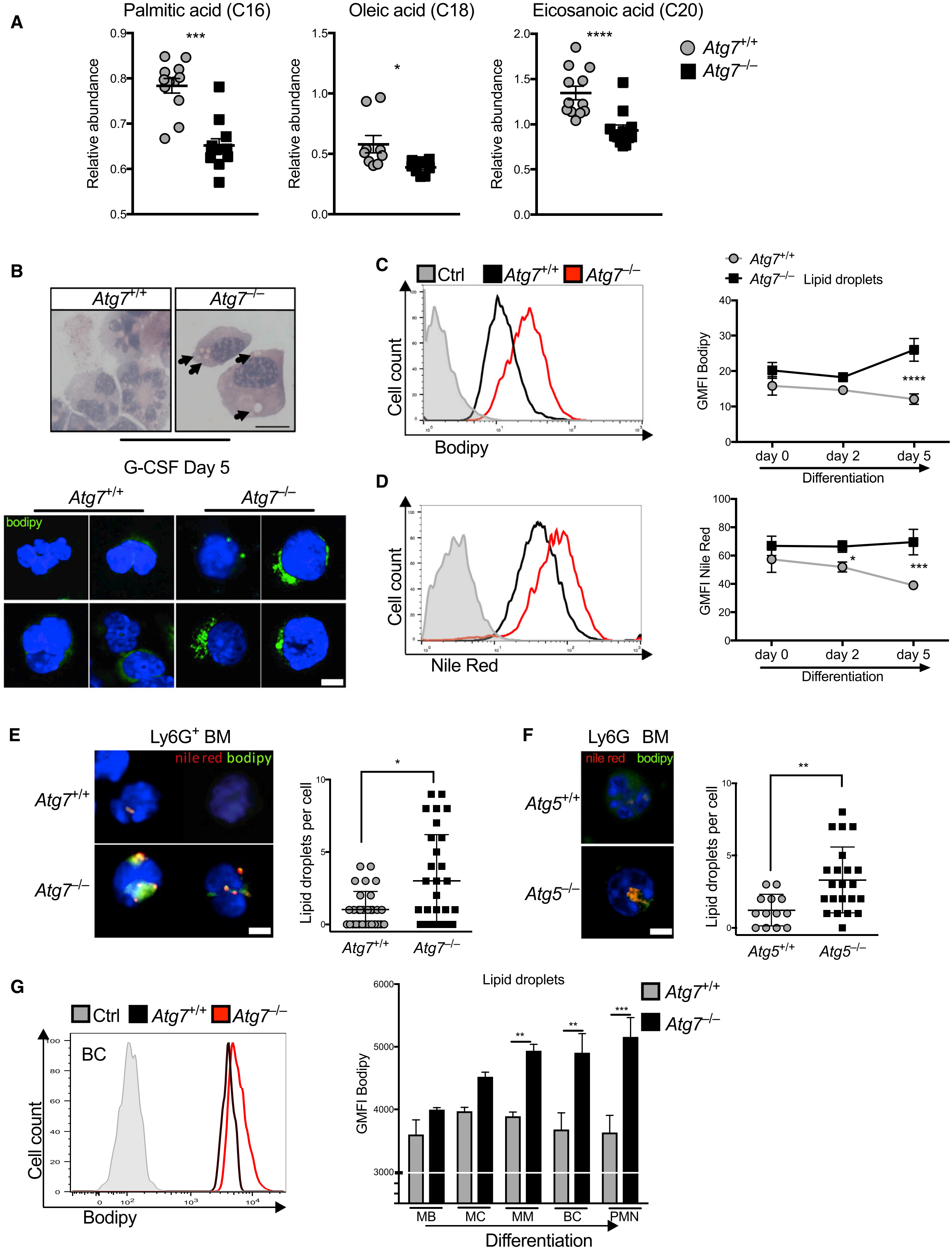

(legend on next page)

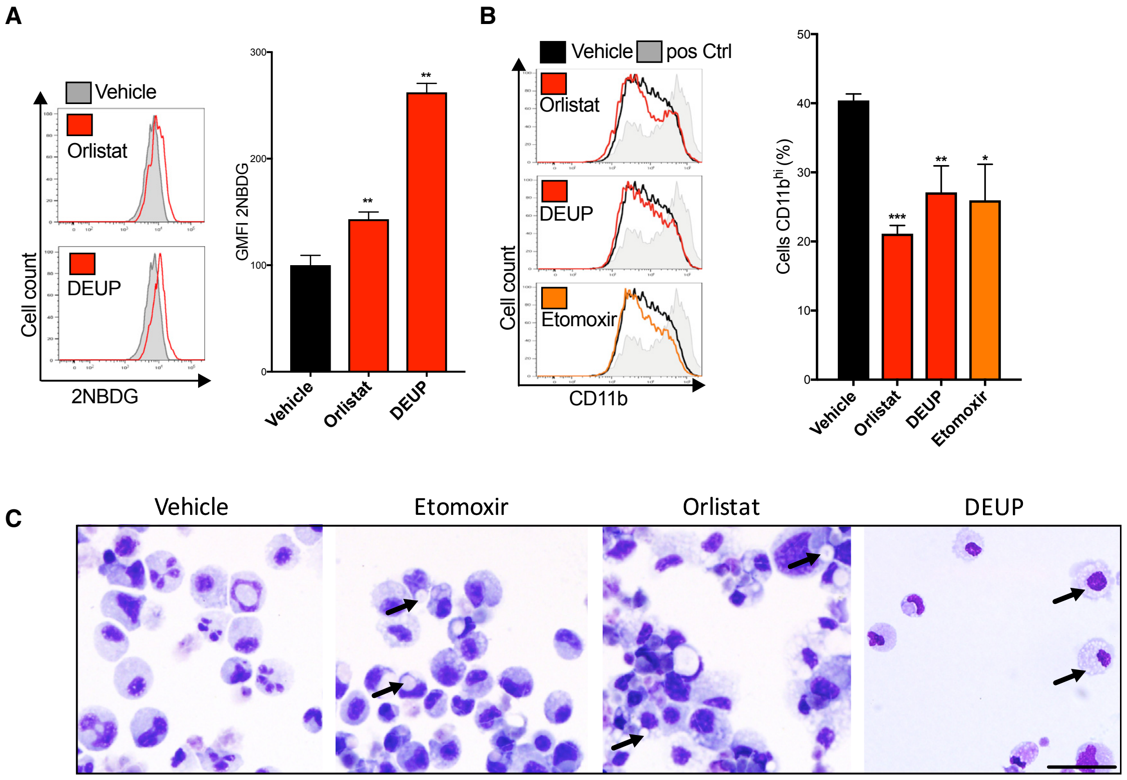

Figure 6. Lysosomal Lipolysis and FAO Are Required for Neutrophil Metabolic Switch and Differentiation

Wild-type myeloblasts were treated with LAL-inhibitor orlistat (100 μM), lipase inhibitor DEUP (5 μM), FAO-inhibitor etomoxir (60 μM), or vehicle control during 5 days of G-induced differentiation. Data representative of three experiments.

(A) Histograms (left) and quantification (right) of fluorescent glucose (2NBDG) uptake as geometric MFI ± SEM from four replicates on day 5 of G-CSF treatment.*p < 0.05, ***p < 0.01.

(B) Histograms (left) and quantification (right) of CD11b maturation marker expression on day 5 of G-CSF-induced differentiation. Ctrl in gray are mature control cells (G-CSF day 7). Quantification shown as mean (±SD) percent of cells CD11b^{hi} from four biological replicates, representative of three independent experiments.

(C) Wright-Giemsa-stained cytospins after inhibitor treatment over 5 days of G-CSF-induced differentiation. Arrows indicate lipid droplets; scale bar, 50 μm.

See also Figure S6.

DISCUSSION

Here we show that autophagy is essential for normal neutrophil differentiation *in vivo*. Our results further show that autophagy provides FFAs via lipid droplet degradation in order to maintain energy-metabolic balance. We conclude that the autophagy-controlled FAO-OXPHOS pathway may be critical to supply sufficient ATP for the energy-demanding process of differentiation.

A couple of pioneering studies describe an essential role for autophagy in the myeloid lineage during monocyte to macrophage differentiation by preventing caspase-mediated cell death (Jacquel et al., 2012; Zhang et al., 2012). We propose that

Figure 5. Autophagy Mediates Lipolysis during Neutrophil Differentiation

(A) Free fatty acid abundance in *Atg7*$^{+/+}$ and CRISPR-*Atg7*$^{-/-}$ clones quantified by GCxGC/MS. Data represent 5–6 biological replicates per genotype from two independent clones. *p < 0.05, ***p < 0.001, ****p < 0.0001.

(B) Wright-Giemsa-stained cytospins (top) and confocal micropgraphs (bottom) from two independent experiments showing lipid droplets stained with Bodipy 493/503 (1 ng/mL) in *Atg7*$^{+/+}$ and CRISPR-*Atg7*$^{-/-}$ clones after culture in G-CSF (100 nM) for 5 days; arrows indicate lipid droplets. Scale bar, 5 μm.

(C and D) Representative histograms (left) and geometric MFI (right) from two experiments showing (C) Bodipy and (D) Nile red lipid-droplet dye in *Atg7*$^{+/+}$ and *Atg7*$^{-/-}$ myeloblasts after the indicated duration of G-CSF (100 nM)-induced differentiation. Histograms are from day 5, gray is unstained control from day 0. *p < 0.05, ***p < 0.001, ****p < 0.0001, tested by two-way ANOVA.

(E and F) Nile red^{+} Bodipy^{+} lipid droplets were quantified by confocal microscopy in isolated total Ly6G^{+} BM cells from (E) *Atg7*$^{+/+}$ and *Atg7*$^{-/-}$ mice or (F) *Atg5*$^{+/+}$ and *Atg5*$^{-/-}$ mice (*Cebpa-cre*$^{+}$ × *Atg5*$^{f/f}$). Confocal micrographs (left; scale bar, 5 μm) and quantification as lipid droplets/cell ± SEM (right). Data in (E) are combined from two independent experiments (n = 3 mice). *p < 0.05, **p < 0.01.

(G) Representative histograms of Bodipy fluorescence in *Atg7*$^{+/+}$ and *Cebpa-cre*$^{+}$ × *Atg7*$^{f/f}$ (*Atg7*$^{-/-}$) precursors (left) and quantification of lipid droplets as the geometric MFI ± SEM from the indicated *Atg7*$^{+/+}$ and *Atg7*$^{-/-}$ BM precursor populations (n = 3 mice), representative of three independent experiments. **p < 0.01, ***p < 0.001, tested by two-way ANOVA.

See also Figure S5.

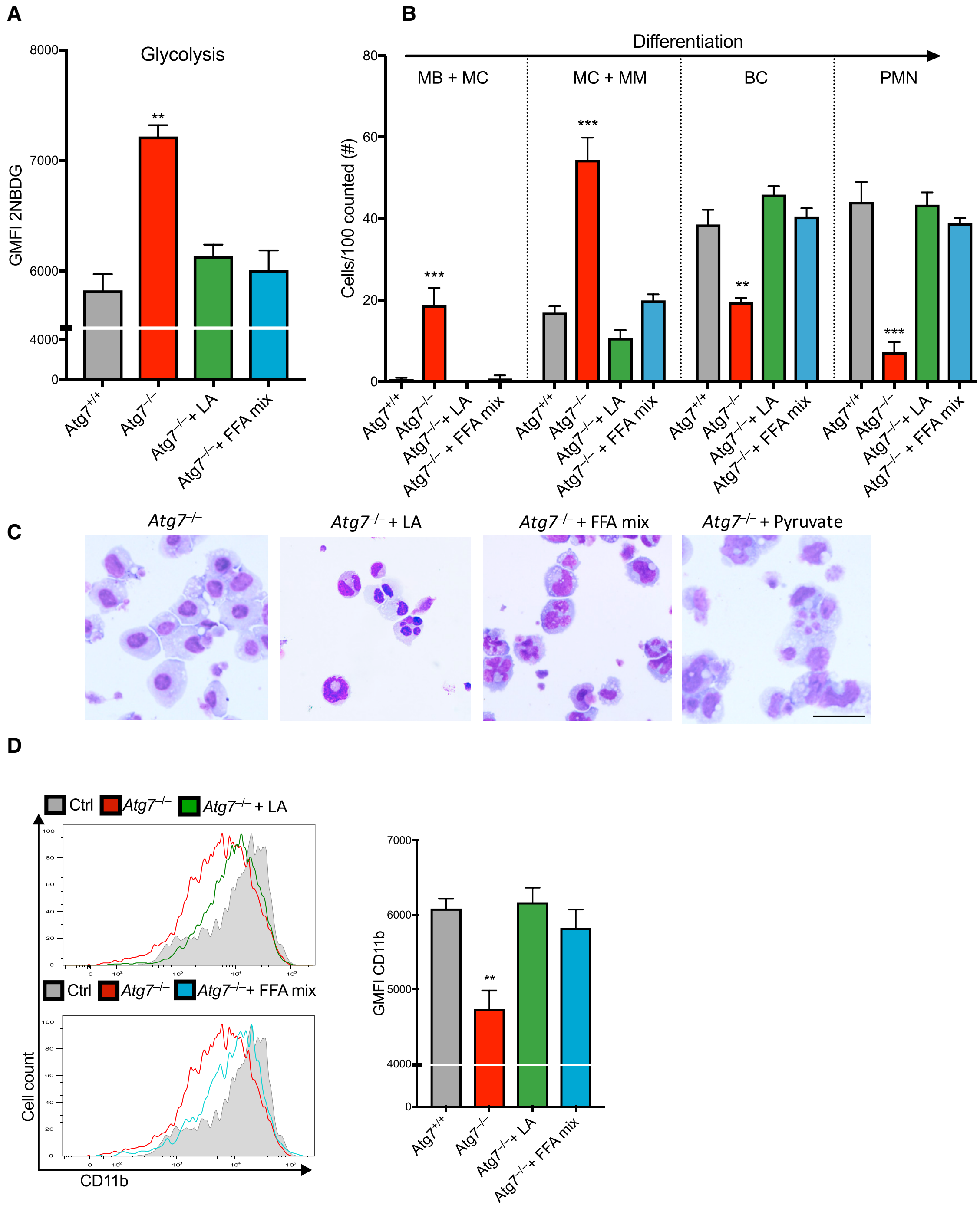

Figure 7. Free Fatty Acids Restore Glucose Metabolism and Differentiation in Autophagy-Deficient Neutrophil Precursors

CRISPR-Atg7$^{+/+}$ and *Atg7*$^{-/-}$ myeloblasts were cultured for 5 days in G-CSF (100 nM) to induce differentiation in the presence of linoleic acid (LA) (10 μg/mL), a mix of saturated and non-saturated fatty acids (FFA mix) (10 μg/mL fatty acids), or vehicle control. Data representative of two experiments.

(A) Quantification of fluorescent glucose (2NBDG) uptake as geometric MFI ± SEM from three independent cultures.

(B) Quantification of indicated maturation stages by morphology. Data are shown as number of cells per 100 cells counted combined from two individual experiments, $^{**}p < 0.01$, $^{***}p < 0.001$.

(C) Representative Wright-Giemsa-stained cytospins from two independent experiments from (B). Scale bar, 50 μm.

(D) Histograms (left) and quantification (right) of CD11b maturation marker expression after day 5 of the indicated treatment in the presence of G-CSF, quantified as mean ± SEM from three biological replicates, $^{**}p < 0.01$.

See also Figure S7.

autophagy may also play a role in sustaining energy metabolism in the monocyte-macrophage lineage differentiation. Supporting this view, work by Ed Pearce et al. demonstrates that the differentiation from monocytes into M2 macrophages requires a shift to OXPHOS which depends on the lysosomal lipase (LAL) (Huang et al., 2014). In addition, in line with our findings, LAL-deficient mice show a similar increase in immature granulocytes as seen in our mouse models, compatible with a defect in granulopoiesis (Qu et al., 2010).

Studies of neutrophil differentiation have so far been limited by the availability of a suitable *cre* system to excise with specificity at the earliest precursor stage. Deleting *Atg7* within HSCs caused expansive myelodysplastic disease and bone marrow failure in mice (Mortensen et al., 2011b), while *Lyz2-cre*-mediated deletion is inefficient in granulocyte precursors. Accordingly, *Lyz2-cre*-mediated *Atg5* deletion had no measurable impact on neutrophil function but caused a mild expansion of precursors (Rožman et al., 2015). Herein we used the *Cebpa* promotor to drive robust *cre*-mediated excision of *Atg7* in >90% of GMP-MB-stage neutrophil precursors, enabling us to study autophagy during early granulopoiesis independent of a defect at the HSC level.

Neutrophil differentiation is driven by G-CSF that signals though the G-CSF receptor for a distinct transcriptional program. Here we have shown that autophagy adds another, metabolic, layer to this purely genetic control of differentiation. Notably, autophagy does not appear to interfere with the rewiring of the transcriptional program, as expression of gene regulators (*Cebpa*) and granule genes is the same in neutrophil precursors at distinct stages regardless of their autophagy status. Because we find a population that expresses Ly6G but has remained morphologically (i.e., fail to segment their nuclei) and functionally (unable to phagocytose) immature, the requirement for autophagy in cellular differentiation may occur in parallel and independent of transcriptional regulators.

A causal link has been implicated between autophagy, metabolism, and differentiation outside of the hematopoietic system, in hepatic stellate cells. Autophagy is critical in the transdifferentiation of hepatic stellate cells into proliferative myofibroblasts, and the addition of FFA rescued the differentiation of autophagy-deficient hepatocytes (Hernández-Gea et al., 2012). Together with our data this suggests that the mechanism described here operates broadly and perhaps universally in cellular differentiation. Notably, the CMA-dependent degradation of lipid droplet-associated proteins such as perilipin 2 (PLIN2) and PLIN3 precedes and facilitates lipolysis in fibroblasts (Kaushik and Cuervo, 2016), demonstrating the existence of an intimate crosstalk between different forms of autophagy in the control of intracellular homeostasis.

While the excessive glycolysis in autophagy-deficient precursors is likely a secondary compensation effect to alleviate the energy crisis, it does not restore ATP balance back to wild-type amounts nor does it restore differentiation. However, exogenous pyruvate at supraphysiological amounts or the addition of free fatty acids (either linoleic acid or a mixture of free fatty acids) restore differentiation. Together, this indicates that in the absence of autophagy, either of these metabolites can correct the energy-metabolic requirements for neutrophil differentiation.

In addition to fatty acid delivery, autophagy is central to FAO and OXPHOS by maintaining mitochondrial health via mitophagy. For example, virus-specific NK cell differentiation into long-lived memory cells requires Bnip3- and Bnip3L-driven mitophagy for mitochondrial maintenance and prevention of ROS (O'Sullivan et al., 2015). While defective mitochondrial turnover and health may also contribute to the observed neutrophil differentiation defect in $Atg7^{-/-}$ and $Atg5^{-/-}$ models, we were unable to improve differentiation with the antioxidant NAC and Bnip3-null mice develop morphologically normal neutrophils (Glick et al., 2012). Whereas Bnip3 deletion allowed targeted disruption of the mitophagy pathway, the receptor that delivers lipid droplets into autophagosomes has not yet been identified. Its identification would enable further insight into the relevance and mechansims of autophagy-mediated lipolysis.

Many myeloid leukemias are characterized by defective granulocyte differentiation with myeloblast accumulation, dysregulated autophagy-gene expression, and Warburg-like aerobic glycolysis, although a causative link between these findings remains to be established. It would therefore be of interest to investigate whether a similar metabolic rewiring dependent on autophagy to provide free fatty acids for FAO and OXPHOS is active during leukemogenesis, potentially caused by the extreme energy demands associated with ongoing proliferation and anabolism in leukemia (Evangelisti et al., 2015). The establishment of autophagy and energy-metabolic adaptation as unique critical regulators of normal granulopoiesis and their mechanistic interaction for an autophagy-FAO-OXPHOS pathway may also be relevant in the context of congenital or radiation-therapy-induced neutropenia.

STAR★METHODS

Detailed methods are provided in the online version of this paper and include the following:

- KEY RESOURCES TABLE
- CONTACT FOR REAGENT AND RESOURCE SHARING
- EXPERIMENTAL MODEL AND SUBJECT DETAILS
  - Mice
  - Cell lines
- METHOD DETAILS
  - 32D CRISPR $Atg7^{-/-}$ myeloblast clone generation
  - Generation of bone marrow chimeric mice
  - *In vitro* neutrophil differentiation assays
  - Flow cytometry
  - Metabolomic analysis by LC/MS/MS and GC/GC/MS
  - Metabolic flux analysis
  - Bacterial killing assay
  - Phagocytosis assay
  - q-PCR
  - Fluidigm gene expression analysis
  - ATP levels
  - L-lactate and glucose quantification
  - Western blot
  - Microscopy
- QUANTIFICATION AND STATISTICAL ANALYSIS

SUPPLEMENTAL INFORMATION

Supplemental Information includes seven figures and one table and can be found with this article online at http://dx.doi.org/10.1016/j.immuni.2017.08.005.

AUTHORS CONTRIBUTIONS

Conceptualization, T.R., A.C., S.E.W.J., and A.K.S.; Methodology, T.R., A.S., P.H., J.McC., H.U., F.C.R., and S.P.; Formal Analysis, T.R. and A.C.; Investigation, T.R., A.S., S.D., P.H., E.J., Z.Y., T.S., and J.McC.; Writing original draft, T.R. and A.K.S.; Writing - Review and Editing, A.C., A.S., H.U., S.E.W.J., and A.K.S.; Supervision, A.K.S. and S.E.J.; and Funding Acquisition, A.K.S. and S.E.W.J. A.K.S. and S.E.W.J. jointly directed this work.

ACKNOWLEDGMENTS

We thank Prof. Ivo Touw, Dr. Mizushima, and Dr. Komatsu for providing mice, Dr. Christoph Thiele and Dr. Erdinc Sezgin for help with microscopy, Hanlin Zhang, Sandrine Obba, and Mariolina Salio for help with experiments, and Petter Woll for help with establishing and analyzing the Fluidigm experiments. Flow cytometry work was performed with the help of Craigh Waugh and Jonathan Webber from the WIMM and Kennedy Institute Flow Facilities, respectively. We would like to thank Marcia Curtinha and the Histology Facility at the Kennedy Institute for Wright-Giemsa stainings. David Ferguson helped with electron microscopy. This work was supported by grants from the Medical Research Council (PhD prize scholarship for T.R.), the Wellcome Trust (Investigator award grant number 103830/Z/14/Z to A.K.S.), and the MRC Molecular Haematology Unit core award (MC_UU_12009/5 to S.E.W.J.). Li-cor Odyssey imager funded by ERC AdG 670930.

Received: February 7, 2017
Revised: May 15, 2017
Accepted: August 14, 2017
Published: September 12, 2017

REFERENCES

Auberger, P., and Puissant, A. (2017). Autophagy, a key mechanism of oncogenesis and resistance in leukemia. Blood *129*, 547–552.

Bardoel, B.W., Kenny, E.F., Sollberger, G., and Zychlinsky, A. (2014). The balancing act of neutrophils. Cell Host Microbe *15*, 526–536.

Bhattacharya, A., Wei, Q., Shin, J.N., Abdel Fattah, E., Bonilla, D.L., Xiang, Q., and Eissa, N.T. (2015). Autophagy is required for neutrophil-mediated inflammation. Cell Rep. *12*, 1731–1739.

Borregaard, N. (2010). Neutrophils, from marrow to microbes. Immunity *33*, 657–670.

Buck, M.D., O’Sullivan, D., Klein Geltink, R.I., Curtis, J.D., Chang, C.H., Sanin, D.E., Qiu, J., Kretz, O., Braas, D., van der Windt, G.J., et al. (2016). Mitochondrial dynamics controls T cell fate through metabolic programming. Cell *166*, 63–76.

Bugl, S., Wirths, S., Müller, M.R., Radsak, M.P., and Kopp, H.-G. (2012). Current insights into neutrophil homeostasis. Ann. N Y Acad. Sci. *1266*, 171–178.

Coort, S.L., Willems, J., Coumans, W.A., van der Vusse, G.J., Bonen, A., Glatz, J.F., and Luiken, J.J. (2002). Sulfo-N-succinimidyl esters of long chain fatty acids specifically inhibit fatty acid translocase (FAT/CD36)-mediated cellular fatty acid uptake. Mol. Cell. Biochem. *239*, 213–219.

Evangelisti, C., Evangelisti, C., Chiarini, F., Lonetti, A., Buontempo, F., Neri, L.M., McCubrey, J.A., and Martelli, A.M. (2015). Autophagy in acute leukemias: A double-edged sword with important therapeutic implications. Biochim. Biophys. Acta *1853*, 14–26.

Galluzzi, L., Pietrocola, F., Levine, B., and Kroemer, G. (2014). Metabolic control of autophagy. Cell *159*, 1263–1276.

Glick, D., Zhang, W., Beaton, M., Marsboom, G., Gruber, M., Simon, M.C., Hart, J., Dorn, G.W., 2nd, Brady, M.J., and Macleod, K.F. (2012). BNip3 regulates mitochondrial function and lipid metabolism in the liver. Mol. Cell. Biol. *32*, 2570–2584.

Guo, J.Y., Teng, X., Laddha, S.V., Ma, S., Van Nostrand, S.C., Yang, Y., Khor, S., Chan, C.S., Rabinowitz, J.D., and White, E. (2016). Autophagy provides metabolic substrates to maintain energy charge and nucleotide pools in Ras-driven lung cancer cells. Genes Dev. *30*, 1704–1717.

Hadváry, P., Sidler, W., Meister, W., Vetter, W., and Wolfer, H. (1991). The lipase inhibitor tetrahydrolipstatin binds covalently to the putative active site serine of pancreatic lipase. J. Biol. Chem. *266*, 2021–2027.

Hara, T., Nakamura, K., Matsui, M., Yamamoto, A., Nakahara, Y., Suzuki-Migishima, R., Yokoyama, M., Mishima, K., Saito, I., Okano, H., and Mizushima, N. (2006). Suppression of basal autophagy in neural cells causes neurodegenerative disease in mice. Nature *441*, 885–889.

Harrison, E.H., Bernard, D.W., Scholm, P., Quinn, D.M., Rothblat, G.H., and Glick, J.M. (1990). Inhibitors of neutral cholesteryl ester hydrolase. J. Lipid Res. *31*, 2187–2193.

Hernández-Gea, V., Ghiassi-Nejad, Z., Rozenfeld, R., Gordon, R., Fiel, M.I., Yue, Z., Czaja, M.J., and Friedman, S.L. (2012). Autophagy releases lipid that promotes fibrogenesis by activated hepatic stellate cells in mice and in human tissues. Gastroenterology *142*, 938–946.

Huang, S.C.-C., Everts, B., Ivanova, Y., O’Sullivan, D., Nascimento, M., Smith, A.M., Beatty, W., Love-Gregory, L., Lam, W.Y., O’Neill, C.M., et al. (2014). Cell-intrinsic lysosomal lipolysis is essential for alternative activation of macrophages. Nat. Immunol. *15*, 846–855.

Iida, S., Watanabe-Fukunaga, R., Nagata, S., and Fukunaga, R. (2008). Essential role of C/EBPalpha in G-CSF-induced transcriptional activation and chromatin modification of myeloid-specific genes. Genes Cells *13*, 313–327.

Jacquel, A., Obba, S., Boyer, L., Dufies, M., Robert, G., Gounon, P., Lemichez, E., Luciano, F., Solary, E., and Auberger, P. (2012). Autophagy is required for CSF-1-induced macrophagic differentiation and acquisition of phagocytic functions. Blood *119*, 4527–4531.

Jiang, Y., and Nakada, D. (2016). Cell intrinsic and extrinsic regulation of leukemia cell metabolism. Int. J. Hematol. *103*, 607–616.

Kabat, A.M., Harrison, O.J., Riffelmacher, T., Moghaddam, A.E., Pearson, C.F., Laing, A., Abeler-Dörner, L., Forman, S.P., Grencis, R.K., Sattentau, Q., et al. (2016). The autophagy gene Atg16l1 differentially regulates Treg and TH2 cells to control intestinal inflammation. eLife *5*, e12444.

Kaushik, S., and Cuervo, A.M. (2016). AMPK-dependent phosphorylation of lipid droplet protein PLIN2 triggers its degradation by CMA. Autophagy *12*, 432–438.

Klionsky, D.J., Abdelmohsen, K., Abe, A., Abedin, M.J., Abeliovich, H., Acevedo Arozena, A., Adachi, H., Adams, C.M., Adams, P.D., Adeli, K., et al. (2016). Guidelines for the use and interpretation of assays for monitoring autophagy (3rd edition). Autophagy *12*, 1–222.

Komatsu, M., Waguri, S., Ueno, T., Iwata, J., Murata, S., Tanida, I., Ezaki, J., Mizushima, N., Ohsumi, Y., Uchiyama, Y., et al. (2005). Impairment of starvation-induced and constitutive autophagy in Atg7-deficient mice. J. Cell Biol. *169*, 425–434.

Kruszynska, Y.T., and Sherratt, H.S. (1987). Glucose kinetics during acute and chronic treatment of rats with 2[6(4-chloro-phenoxy)hexyl]oxirane-2-carboxylate, etomoxir. Biochem. Pharmacol. *36*, 3917–3921.

Kuhajda, F.P., Pizer, E.S., Li, J.N., Mani, N.S., Frehywot, G.L., and Townsend, C.A. (2000). Synthesis and antitumor activity of an inhibitor of fatty acid synthase. Proc. Natl. Acad. Sci. USA *97*, 3450–3454.

Latif, A.-L., and Holyoake, T.L. (2016). Lifting the differentiation embargo. Cell *167*, 45–46.

Lee, I.H., Kawai, Y., Fergusson, M.M., Rovira, I.I., Bishop, A.J.R., Motoyama, N., Cao, L., and Finkel, T. (2012). Atg7 modulates p53 activity to regulate cell cycle and survival during metabolic stress. Science *336*, 225–228.

Liu, K., and Czaja, M.J. (2013). Regulation of lipid stores and metabolism by lipophagy. Cell Death Differ. *20*, 3–11.

Mayer, N., Schweiger, M., Romauch, M., Grabner, G.F., Eichmann, T.O., Fuchs, E., Ivkovic, J., Heier, C., Mrak, I., Lass, A., et al. (2013). Development

of small-molecule inhibitors targeting adipose triglyceride lipase. Nat. Chem. Biol. *9*, 785–787.

Mizushima, N., Yamamoto, A., Matsui, M., Yoshimori, T., and Ohsumi, Y. (2004). In vivo analysis of autophagy in response to nutrient starvation using transgenic mice expressing a fluorescent autophagosome marker. Mol. Biol. Cell *15*, 1101–1111.

Mochida, K., Oikawa, Y., Kimura, Y., Kirisako, H., Hirano, H., Ohsumi, Y., and Nakatogawa, H. (2015). Receptor-mediated selective autophagy degrades the endoplasmic reticulum and the nucleus. Nature *522*, 359–362.

Mortensen, M., Soilleux, E.J., Djordjevic, G., Tripp, R., Lutteropp, M., Sadighi-Akha, E., Stranks, A.J., Glanville, J., Knight, S., Jacobsen, S.-E.W., et al. (2011a). The autophagy protein Atg7 is essential for hematopoietic stem cell maintenance. J. Exp. Med. *208*, 455–467.

Mortensen, M., Watson, A.S., and Simon, A.K. (2011b). Lack of autophagy in the hematopoietic system leads to loss of hematopoietic stem cell function and dysregulated myeloid proliferation. Autophagy *7*, 1069–1070.

Nauseef, W.M., and Borregaard, N. (2014). Neutrophils at work. Nat. Immunol. *15*, 602–611.

Nicolás-Ávila, J.Á., Adrover, J.M., and Hidalgo, A. (2017). Neutrophils in homeostasis, immunity, and cancer. Immunity *46*, 15–28.

O'Sullivan, D., van der Windt, G.J.W., Huang, S.C.-C., Curtis, J.D., Chang, C.-H., Buck, M.D., Qiu, J., Smith, A.M., Lam, W.Y., DiPlato, L.M., et al. (2014). Memory CD8(+) T cells use cell-intrinsic lipolysis to support the metabolic programming necessary for development. Immunity *41*, 75–88.

O'Sullivan, T.E., Johnson, L.R., Kang, H.H., and Sun, J.C. (2015). BNIP3- and BNIP3L-mediated mitophagy promotes the generation of natural killer cell memory. Immunity *43*, 331–342.

Pearce, E.L., Walsh, M.C., Cejas, P.J., Harms, G.M., Shen, H., Wang, L.-S., Jones, R.G., and Choi, Y. (2009). Enhancing CD8 T-cell memory by modulating fatty acid metabolism. Nature *460*, 103–107.

Puleston, D.J., Zhang, H., Powell, T.J., Lipina, E., Sims, S., Panse, I., Watson, A.S., Cerundolo, V., Townsend, A.R., Klenerman, P., and Simon, A.K. (2014). Autophagy is a critical regulator of memory CD8(+) T cell formation. eLife *3*, e03706.

Qu, P., Shelley, W.C., Yoder, M.C., Wu, L., Du, H., and Yan, C. (2010). Critical roles of lysosomal acid lipase in myelopoiesis. Am. J. Pathol. *176*, 2394–2404.

Remijsen, Q., Vanden Berghe, T., Wirawan, E., Asselbergh, B., Parthoens, E., De Rycke, R., Noppen, S., Delforge, M., Willems, J., and Vandenabeele, P. (2011). Neutrophil extracellular trap cell death requires both autophagy and superoxide generation. Cell Res. *21*, 290–304.

Riffelmacher, T., and Simon, A.-K. (2017). Mechanistic roles of autophagy in hematopoietic differentiation. FEBS J. *284*, 1008–1020.

Rodrigues, H.G., Vinolo, M.A., Magdalon, J., Fujiwara, H., Cavalcanti, D.M., Farsky, S.H., Calder, P.C., Hatanaka, E., and Curi, R. (2010). Dietary free oleic and linoleic acid enhances neutrophil function and modulates the inflammatory response in rats. Lipids *45*, 809–819.

Rožman, S., Yousefi, S., Oberson, K., Kaufmann, T., Benarafa, C., and Simon, H.U. (2015). The generation of neutrophils in the bone marrow is controlled by autophagy. Cell Death Differ. *22*, 445–456.

Salio, M., Puleston, D.J., Mathan, T.S.M., Shepherd, D., Stranks, A.J., Adamopoulou, E., Veerapen, N., Besra, G.S., Hollander, G.A., Simon, A.K., and Cerundolo, V. (2014). Essential role for autophagy during invariant NKT cell development. Proc. Natl. Acad. Sci. USA *111*, E5678–E5687.

Satake, S., Hirai, H., Hayashi, Y., Shime, N., Tamura, A., Yao, H., Yoshioka, S., Miura, Y., Inaba, T., Fujita, N., et al. (2012). C/EBPβ is involved in the amplification of early granulocyte precursors during candidemia-induced "emergency" granulopoiesis. J. Immunol. *189*, 4546–4555.

Settembre, C., Di Malta, C., Polito, V.A., Garcia Arencibia, M., Vetrini, F., Erdin, S., Erdin, S.U., Huynh, T., Medina, D., Colella, P., et al. (2011). TFEB links autophagy to lysosomal biogenesis. Science *332*, 1429–1433.

Singh, R., Kaushik, S., Wang, Y., Xiang, Y., Novak, I., Komatsu, M., Tanaka, K., Cuervo, A.M., and Czaja, M.J. (2009). Autophagy regulates lipid metabolism. Nature *458*, 1131–1135.

Tehranchi, R., Woll, P.S., Anderson, K., Buza-Vidas, N., Mizukami, T., Mead, A.J., Astrand-Grundström, I., Strömbeck, B., Horvat, A., Ferry, H., et al. (2010). Persistent malignant stem cells in del(5q) myelodysplasia in remission. N. Engl. J. Med. *363*, 1025–1037.

Valtieri, M., Tweardy, D.J., Caracciolo, D., Johnson, K., Mavilio, F., Altmann, S., Santoli, D., and Rovera, G. (1987). Cytokine-dependent granulocytic differentiation. Regulation of proliferative and differentiative responses in a murine progenitor cell line. J. Immunol. *138*, 3829–3835.

Watson, A.S., Riffelmacher, T., Stranks, A., Williams, O., De Boer, J., Cain, K., MacFarlane, M., McGouran, J., Kessler, B., Khandwala, S., et al. (2015). Autophagy limits proliferation and glycolytic metabolism in acute myeloid leukemia. Cell Death Dis. *1*, 15008.

Wei, J., Long, L., Yang, K., Guy, C., Shrestha, S., Chen, Z., Wu, C., Vogel, P., Neale, G., Green, D.R., and Chi, H. (2016). Autophagy enforces functional integrity of regulatory T cells by coupling environmental cues and metabolic homeostasis. Nat. Immunol. *17*, 277–285.

Wölfler, A., Danen-van Oorschot, A.A., Haanstra, J.R., Valkhof, M., Bodner, C., Vroegindeweij, E., van Strien, P., Novak, A., Cupedo, T., and Touw, I.P. (2010). Lineage-instructive function of C/EBPα in multipotent hematopoietic cells and early thymic progenitors. Blood *116*, 4116–4125.

Xu, X., Araki, K., Li, S., Han, J.-H., Ye, L., Tan, W.G., Konieczny, B.T., Bruinsma, M.W., Martinez, J., Pearce, E.L., et al. (2014). Autophagy is essential for effector CD8(+) T cell survival and memory formation. Nat. Immunol. *15*, 1152–1161.

Zeng, H., Yang, K., Cloer, C., Neale, G., Vogel, P., and Chi, H. (2013). mTORC1 couples immune signals and metabolic programming to establish T(reg)-cell function. Nature *499*, 485–490.

Zhang, D.-E., Zhang, P., Wang, N.D., Hetherington, C.J., Darlington, G.J., and Tenen, D.G. (1997). Absence of granulocyte colony-stimulating factor signaling and neutrophil development in CCAAT enhancer binding protein α-deficient mice. Proc. Natl. Acad. Sci. USA *94*, 569–574.

Zhang, Y., Morgan, M.J., Chen, K., Choksi, S., and Liu, Z.G. (2012). Induction of autophagy is essential for monocyte-macrophage differentiation. Blood *119*, 2895–2905.

STAR★METHODS

KEY RESOURCES TABLE

REAGENT or RESOURCE	SOURCE	IDENTIFIER
Antibodies		
PE anti-mouse CD34 Antibody, Clone HM34	BioLegend	Cat#152204; RRID: AB_313659
PE/Cy7 anti-mouse Ly-6G Antibody, Clone 1A8	BioLegend	Cat#127618; RRID: AB_1877261
APC/Cy7 anti-mouse CD117 (c-kit) Antibody, Clone 2B8	BioLegend	Cat#105826; RRID: AB_2632809
APC anti-mouse TER-119/Erythroid Cells Antibody, Clone Ter119	BioLegend	Cat#116212; RRID: AB_313713
PE anti-mouse CD11b Antibody, Clone M1/70	BioLegend	Cat#101208; RRID: AB_312790
APC anti-mouse/human CD45R/B220 Antibody	BioLegend	Cat#103212; RRID: AB_312996
APC anti-mouse CD8a Antibody, Clone 53-6.7	BioLegend	Cat#100712; RRID: AB_312751
APC anti-mouse CD19 Antibody, Clone 6D5	BioLegend	Cat#115511; RRID: AB_313646
Anti-mouse monoclonal G-CSF R/CD114 Antibody (723806); Alexa Fluor-405	R&D Systems	Cat#MAB6039; RRID: AB_10890232
APC anti-mouse CD4 Antibody, Clone GK1.5	BioLegend	Cat#100412; RRID: AB_312696
Alexa Fluor 488 anti-mouse CD36 Antibody, Clone G8.8	BioLegend	Cat#102607; RRID: AB_1134107
Bacterial and Virus Strains		
Staphylococcus aureus	ATCC	ATCC 29213
Chemicals, Peptides, and Recombinant Proteins		
May Grünwald Wright Giemsa stain	Thermo Scientific	Cat#69027
Glut1.RBD reagent GFP labeled	Metaflora	Cat#Glut1-G25
Recombinant murine IL-3	Peprotech	Cat#213-13-100
Recombinant murine G-CSF	Peprotech	Cat#250-05-100
Lysostaphin from Staphylococcus	Sigma Aldrich	Cat#L7386
Etomoxir sodium salt hydrate	Sigma Aldrich	Cat#E1905
Diethyl-umbelliferyl phosphate	Sigma Aldrich	Cat#D7692
Orlistat	Sigma Aldrich	Cat#O4139
TaqMan Gene Expression Master Mix	Life Technologies	Cat#4369016
Critical Commercial Assays		
ATP BIOLUMINESCENCE ASSAY KIT HS II	Sigma Aldrich	Cat# 11699709001
Anti-Ly-6G MicroBead Kit, mouse	Miltenyi Biotec	Cat#130-092-332
SuperScript III Platinum One-Step qRT-PCR Kit	Thermo Fisher	Cat#11732020
Glycolysis Cell-Based Assay Kit	Cambridge Bioscience	Cat#600450
Seahorse CF Cell Mito and Glycolysis Stress Test Kits	Agilent	Cat#103015
GLUCOSE (GO) Assay kit	Sigma Aldrich	Cat#GAGO20
Experimental Models: Cell Lines		
32Dcl3 Cell line	ATCC	ATCC; CRL-11346<br>RRID; CVCL_0119
Experimental Models: Organisms/Strains		
Mouse; *Atg7* flox, $Atg7^{tm1Tchi}$; B6.Cg-Atg7 < tm1Tchi >	Komatsu et al., 2005	MGI: 3587769
Mouse; *Atg5* flox, B6.129S-Atg5 < tm1Myok >	Riken	RBRC: 02975
Mouse; *Mx1-cre*; C.Cg-Tg(Mx1-cre)1Cgn/J	Jackson Laboratories	JAX: #005673
Mouse; *Vav-cre*; B6.Cg-Tg(Vav1-icre)A2Kio/J	Jackson Laboratories	JAX: #008610
Mouse: *Cebpa-cre*; $Cebpa^{tm1.1(cre)Touw}$	Wölfler et al., 2010	MGI: 4867437
Oligonucleotides		
Mx1Cre 1 5′ -CAT GTG TCT TGG TGG GCT GAG-3′	Sigma Oligo	N/A
Mx1Cre 2 5′ -CGC ATA ACC AGT GAA ACA GCA T-3′	Sigma Oligo	N/A
CebpaCre FW: 5′ -CGA TGC AAC GAG TGA TGA GGT TC-3′	Sigma Oligo	N/A

(*Continued on next page*)

Continued		
REAGENT or RESOURCE	SOURCE	IDENTIFIER
CebpaCre RV: 5′ -GCA CGT TCA CCG GCA TCA AC-3′	Sigma Oligo	N/A
Atg7 WT PCR 5′ -CCA TGC TGA TGG CTA ATG TCT C- 3′	Sigma Oligo	N/A
Atg7 flox FW 5′ -CTG CAG GAA TTC GAT ATC ATA ACT TCG- 3′	Sigma Oligo	N/A
Atg7 flox RV 5′ -GTC CAG AGT CCG GTC TCT GGT TG- 3′	Sigma Oligo	N/A
Atg5 flox Primer A 5′-GAA TAT GAA GGC ACA CCC CTG AAA TG-3′	Sigma Oligo	N/A
Atg5 flox Primer B 5′-GTA CTG CAT AAT GGT TTA ACT CTT GC-3′	Sigma Oligo	N/A
Atg5 flox Primer C 5′-ACA ACG TCG AGC ACA GCT GCG CAA GG-3′	Sigma Oligo	N/A
Software and Algorithms		
Graphpad Prism v. 7	www.graphpad.com	N/A
FlowJo v. 10	www.flowjo.com	N/A
R version 3.3.3	https://www.r-project.org/	N/A
Fiji	https://fiji.sc	N/A

CONTACT FOR REAGENT AND RESOURCE SHARING

Further information and requests for resources and reagents should be directed to and will be fulfilled by the Lead Contact, Katja Simon (katja.simon@kennedy.ox.ac.uk).

EXPERIMENTAL MODEL AND SUBJECT DETAILS

Mice

Cebpa-cre mice (Wölfler et al., 2010) were from Prof. Ivo Touw and crossed with *Atg7*$^{f/f}$ (Komatsu et al., 2005) or *Atg5*$^{f/f}$ (Hara et al., 2006) mice to obtain *Cebpa-cre*$^{+}$ x *Atg7*$^{f/f}$ and *Cebpa-cre*$^{+}$ x *Atg5*$^{f/f}$ mice. *Vav-cre* and *Mx1-cre* mice were crossed with *Atg7*$^{f/f}$ and *Atg5*$^{f/f}$ mice, respectively, to generate *Vav-cre* x *Atg7*$^{f/f}$ and *Mx1-cre*$^{+}$ x *Atg5*$^{f/f}$ mice. *Cre*$^{-}$ x *Atg7*$^{f/f}$ mice were used in all experiments as controls and were littermates. Male and female mice were used at equal numbers at the age of 6-10 weeks and did not undergo previous procedures except were indicated. C57BL/6 SJL CD45.1 mice for bone marrow chimeras were purchased from Biomedical Services, Oxford. All mice were fully backcrossed to C57BL/6 and housed under specific pathogen-free conditions, up to 7 mice per cage, at Biomedical Services Oxford, and fed standard pellet chow and reverse osmosis water *ad libitum*. Animal experiments were approved by the local ethical review committee and performed under UK project license PPL 30/3388.

Cell lines

32D-cl3 (32D) myeloblast cells, RRID CVCL_0119, are derived from *Mus musculus*, were purchased from ATCC and cultured in RPMI 1640 with 10% FCS, 2 mM L-glutamine,100 U/ml Pen-Strep, and 10 ng/ml IL-3 at 37°C, 5% CO_2. The sex of this cell line is unknown.

METHOD DETAILS

32D CRISPR *Atg7*$^{-/-}$ myeloblast clone generation

Using 32D-cl3 cells, three independent CRISPR *Atg7*$^{-/-}$ clones were generated with sgRNAs from dsODN into pX458 (Feng Zhang, Addgene 48138) plasmids and individually tested in Surveyor assays. Different regions in exons 3-4 of the *Atg7* gene were targeted by plasmid transfection via electroporation and single cells were flow-sorted based on GFP-expression 3 days later. Single cell-derived clones were grown in IL-3 supplemented medium and frameshift deletion confirmed by target site sequencing and western blotting.

Generation of bone marrow chimeric mice

For *Atg5*$^{f/f}$ x *Mx1-cre* (pIpC inducible) chimeras, BM was extracted from tibia and femur of a single 8-week old *Atg5*$^{f/f}$ x *Mx1-cre*$^{-}$, *Atg5*$^{f/f}$ x *Mx1-cre*$^{+}$ (both CD45.2^{+}), and C57BL/6 SJL mouse (CD45.1^{+}). 2x10^{6} CD45.2^{+} BM cells were added to 2x10^{6} CD45.1^{+} BM cells (1:1 CD45.2^{+}:CD45.1^{+}) in a total volume of 200 μL PBS. The 1:1 BM mix was injected i.v 2h after lethal irradiation (450cGy twice, 4 hours apart) into lethally irradiated C57BL/6 SJL CD45.1^{+} recipient mice. 6 weeks post-transplantation, mice were injected i.p. with 250 μg pIpC in 250 μL saline or 250 μL PBS to induce expression of *Mx1-cre* and monitored over a time of 18 weeks. After 18 weeks, BM was extracted and analyzed by flow cytometry. Chimeric mice that received *Atg5*$^{f/f}$ *Mx1-cre*$^{-}$ donor

type cells (CD45.2) were termed control chimera, mice with Atg5$^{f/f}$ *Mx1-cre*$^{+}$ CD45.2 cells were termed Atg5-deficient chimera (*Atg5*$^{-/-}$). This experiment was repeated twice.

In vitro neutrophil differentiation assays

For neutrophil differentiation, 32D-cl3 (32D) myeloblast cells were washed twice with 50 mL PBS to remove IL-3 and resuspended at 2×10^5 cells/ml in medium containing 100 ng/ml recombinant murine G-CSF (peprotech). In experiments involving drug treatments, orlistat (100 μM), methyl-pyruvate (2 mM), di-ethyl-umbelliferyl-phosphate (DEUP, 20 μM), etomoxir (60 μM), C75 (10 μM), SSO (100 μM), linoleic acid (10 μg/ml), FFA mix (10 μg/ml each linoleic, linolenic, myristic, oleic, palmitic and stearic acids + 0.22 mg/ml cholesterol) (all from Sigma) were added to the cells in G-CSF containing differentiation medium for the time indicated. Controls were treated with appropriate vehicle (H_2O, PBS or DMSO). DEUP has been shown to inhibit the neutral cholesteryl ester hydrolase activity responsible for hydrolysis of cellular lipid droplet cholesteryl esters (Harrison et al., 1990). Orlistat is an irreversible, selective inhibitor of serine-containing active site triglyceride lipases (Hadváry et al., 1991). Etomoxir (2[6(4-chlorophenoxy)hexyl]oxirane-2-carboxylate) is an irreversible inhibitor of carnitine palmitoyltransferase-1 on the outer face of the inner mitochondrial membrane (Kruszynska and Sherratt, 1987). Atglistatin is a selective small molecule inhibitor for the rate limiting cytosolic triglyceride lipase *Atgl* (Mayer et al., 2013). Sulfo-N-succinimidyl oleate (SSO) allosterically inhibits the uptake of fatty acids via FAT/CD36 (Coort et al., 2002). C75 is a chemically stable synthetic inhibitor of fatty acid synthase (*fasn*) (Kuhajda et al., 2000). All cell culture experiments included at least three biological replicates per experiment and were repeated twice, unless stated otherwise in figure legends.

Flow cytometry

Fluorochrome-conjugate monoclonal antibodies were purchased from eBioscience, BD Bioscience, or Biolegend. Total Ly6G^{+} cells (which include MM, BC, PMN) were purified using MACS columns were indicated. Staining was performed as previously described (Puleston et al., 2014). GLUT-1 was measured through binding to its ligand, the receptor binding domain (RBD) of a recombinant glycoprotein from the human T lymphotrophic virus (HTLV) fused to eGFP (RBD-eGFP, Metaflora). For mitochondrial mass analysis, cells were stained with MitoTracker Green (Life Technologies) at 150 nM in PBS, 2% FCS for 30 min at 37°C after surface antibody staining. For glucose uptake measurements, cells were incubated in media containing 50 μg/ml 2-NBDG for 20 min at 37°C after surface antibody staining. For lipid droplet quantification, cells were incubated in media containing 1000 ng/ml Bodipy at 37°C for 30 min. Cells were analyzed using Canto III or LSR II flow cytometers (BD Biosciences). All flow cytometry experiments included at least three biological replicates per experiment and were repeated at least twice, unless stated otherwise in figure legends.

Metabolomic analysis by LC/MS/MS and GC/GC/MS

Metabolites were extracted from 5x10^{6} cells of the indicated genotype and differentiation stages (grown in cell culture flasks) by addition of 500 μL of ice cold 80% aqueous methanol. The supernatants were combined and filtered using a 3KD ultrafilter (Millipore), dried in a SpeedVac and subsequently stored at −80°C. On the day of analysis, the dried extracts were re-constituted in 60 μL of ice cold 80% aqueous methanol. A quality control (QC) sample was made by combing 5 μL of each sample. This was injected at the start of the sequence and subsequently every 10 samples throughout the LC/MS/MS analysis.

Metabolites were analyzed using a Thermo Scientific ICS-5000+ ion chromatography system coupled directly to a Q-Exactive HF Hybrid Quadrupole-Orbitrap mass spectrometer with a HESI II electrospray ionisation source (Thermo Scientific). The ICS-5000+ HPLC system incorporated an electrolytic anion generator (KOH) which was programmed to produce an OH^{-} gradient over 37 min for anion exchange chromatography. An inline electrolytic suppressor removed the OH^{-} ions and cations from the post-column eluent prior to MS analysis (Thermo Scientific Dionex AERS 500). A 10 μL partial loop injection was used for all analyses and the chromatographic separation was performed using a Thermo Scientific Dionex IonPac AS11-HC 2 × 250 mm, 4 μm particle size column with a Dionex Ionpac AG11-HC 4 μm 2x50 guard column inline. The IC flow rate was 0.250 mL/min. The total run time was 37 min and the hydroxide ion gradient comprised as follows: 0mins, 0mM; 1min, 0mM; 15mins, 60mM; 25mins, 100mM; 30mins, 100mM; 30.1mins, 0mM; 37mins, 0mM. Analysis was performed in negative ion mode using a scan-range from 80-900 and resolution set to 70,000. The tune file source parameters were set as follows: Sheath gas flow 60 mL/min; Aux gas flow 20 mL/min; Spray voltage 3.6v; Capillary temperature 320°C; S-lens RF value 70; Heater temperature 450°C. AGC target was set to 1e6v ions and the Max IT value was 250ms. The column temperature was kept at 30°C throughout the experiment. Full scan data were acquired in continuum mode.

Raw data files were processed using ProgenesisQI (Waters, Elstree). This process included alignment of retention times, identified the presence of natural abundance isotope peaks, identified adducts forms and was then used to identify compounds present. The retention times and other measurable characteristics for each metabolite were confirmed by comparing values from the experimental data with the same values from the analysis of authentic standards for each metabolite. The measured criteria compared where a combination of accurate mass analysis (< 5ppm), retention time (+/− 15 s), fragmentation pattern and isotope pattern matching (> 90%). Principal Component Analysis was also performed using ProgenesisQI from peas representing all 4215 compounds measured with a %CV < 30 in the quality control samples (normalized to all compounds).

For lipidomics, 5x10^{6} cells were homogenized in 400 μl of 50% MeOH/H_2O in a beads beater tube. Myristic-acid-14,14,14-d_3 (1mg/ml) standard was spiked at ratio 1 μg/5x10^{6} cells and samples were vortexed for 5 min after adding 1 mL of tert-butyl methyl ether (MTBE). After centrifugation at 13,000 RPM supernatants were collected into a 5:1 MeOH/H_2O solution. The aqueous phase was dried in a speedvac and combined with the dried MeOH pellet. For rederivatization, dried metabolite extraction was re-suspended in 20 μg/ul MOX (volume 50 μl) and reacted at 30°C for 90 min on vortex. 30 μl of pyridine and 70 μl of MSTFA were added

to react at 60°C for 60 min before direct injection into GCxGC-MS system comprising of a gas chromatograph coupled to a quadrupole mass spectrometer (Shimadzu GCMS QP2010 Ultra) and a Shimadzu AOC-20i/s auto sampler as described. The first dimension separation was carried out on a SHM5MS capillary column (30 mÅ~0.25 mm i.d.Å~0.25 μm film thickness, Shimadzu) while the second dimension separation was on a BPX-50 capillary column (5 mÅ~0.15 mm i.d.Å~0.15 μm film thickness, SGE). Helium gas was used as a carrier gas at a 73 psi constant inlet head pressure. The modulation period was set as 6 s. The samples were injected at 280°C in different split ratios (between 1:1 to 1:200). The oven temperature was programed from 60°C to 320°C at 10°C/min and held at 320°C for 8 min. The interface temperature to the mass spectrometer was set at 330°C and ion source was heated at 230°C. The MS was operated at scan speeds between 5000 and 20,000 amu covering a range of m/z 45–600. Electron Ionization spectra were recorded at 70 eV.

Metabolic flux analysis

The real-time extracellular acidification rate (ECAR) and oxygen consumption rate (OCR) were measured using a XF 96 extracellular flux analyzer (Seahorse Bioscience). 5×10^4 32D cells (CRISPR-*Atg7*$^{-/-}$ and *Atg7*$^{+/+}$ clones) or 2×10^5 freshly isolated Ly6G$^+$ BM neutrophils were washed twice in RPMI 1640 without sodium bicarbonate, 20 mM glucose, 1% FCS, 2mM pyruvate and seeded in corresponding assay medium in a XF plate coated with poly-L-lysine (Sigma). Cells were rested for 1 hour at 37°C before analysis. Two independent experiments were performed with at least four independent replicates per group.

Bacterial killing assay

Neutrophil intracellular anti-bacterial activity was assessed using the lysostaphin protection assay. *S. aureus* (NTCC 6571) were freshly cultured and opsonized with mouse serum (final concentration 12.5% in RPMI1640) at 37°C for 30 min. Mouse BM-derived Ly6G$^+$ granulocytes (2.5x10^5 cells/well) were infected in a 96-well round-bottom plate in 25 μL RPMI1640 + 10% FCS at MOI of 10 bacteria / neutrophil. Following 45 min of infection, cells were washed with warm PBS and cultured in RPMI + 10% FCS containing 100 μg/mL lysostaphin at 37°C. After 45 min, neutrophils were washed twice with PBS and lysed in 1%-Triton X-100 in dH_2O. Lysate was serially diluted, plated on agar plates by track method and CFU were counted next day. Results were scored as absolute numbers of CFU per ml, with a minimum of 50 colonies counted per group in four independent replicates.

Phagocytosis assay

Neutrophil phagocytosis was assessed by flow-cytometric quantification of fluorescent bead uptake during 1 hour incubation in the presence of 1 μg/ml LPS. pH-sensitive phRodo zymosan-beads (Invitrogen) were used except where indicated otherwise. Surface marker staining was performed after phagocytosis and data quantified as % of cells bead+ from three independent experiments with four mice per group.

q-PCR

RNA was extracted using RNeasy Kit (QIAGEN) and quantified using a Nanodrop spectrophotometer (Thermo Scientific). RNA was reverse transcribed (RT) using a High Capacity RNA to cDNA kit (Applied Biosystems (AB)). Resulting cDNA was stored at −20°C. Real-time quantitative PCR using comparative Ct method (ΔΔCt) was utilized to evaluate gene expression using validated TaqMan probes (AB) on a 7500 Fast Real-time PCR machine (AB). Conditions: (1) 50°C, 2 min; (2) 95°C, 10 min; (3) 95°C 15 s; (4) 60°C 1 min; 40 cycles of 3–4. All results were normalized to actin, gapdh and hprt expression after which the best housekeeping gene was selected for further analysis. The assay IDs for the primers of the analyzed genes are as follows: Mm00504340_m1 (*Atg5*), Mm00512209_m1 (*Atg7*), Mm01545399_m1 (*Hprt*), Mm99999915_g1 (*Gapdh*), Mm00607939_s1 (*Actin*), Mm01298424_m1 (*Mpo*), Mm00442991_m (*Mmp9*), Mm00434787_m1 (*Ltf*), Mm00514283_s1 (*Cebpa*),

Fluidigm gene expression analysis

32D CRISPR-*Atg7*$^{-/-}$ and *Atg7*$^{+/+}$ clones were flow-sorted (two hundred cells/population) from independent clones into OneStep lysis buffer (Invitrogen). For *in vivo* experiments, 100 bone marrow cells were sorted per population. RNA was reverse transcribed and cDNA was pre-amplified using the CellsDirect OneStep q-RT kit (Invitrogen). The selected autophagy, apoptotic and metabolic genes were amplified and analyzed for expression using a dynamic 48x48 array (Biomark Fluidigm) as previously described (Tehranchi et al., 2010). Data were analyzed using the $2^{-\Delta\Delta Ct}$ method, and all results were normalized to *Actin*, *B2m* and *Hprt* expression after which the best housekeeping gene was selected for further analysis. Biological replicates represent individual mice in each experiment.

ATP levels

Total bone marrow cells were isolated from *Cebpa-cre*$^+$ x *Atg7*$^{f/f}$ and littermate controls. Ly6G$^+$ cells (which include MM, BC, PMN) were purified using MACS columns (Miltenyi Biotec). Cell count was normalized to 1x10^6 cells per sample and ATP levels from cell pellets were determined by using the luciferase based ATP Bioluminescence Assay Kit HS II (Roche Applied Science). ATP concentration was calculated from intersection of sample luminescence values with ATP standard. Independent biological replicates represent individual mice in each experiment.

L-lactate and glucose quantification

32D CRISPR-*Atg7*$^{-/-}$ and *Atg7*$^{+/+}$ clones were cultured in RPMI 1640 (containing 2mg/mL Glucose) with 1% FCS, 2 mM L-glutamine, 100 U/ml Pen-Strep. In order to induce neutrophil differentiation, 1x10^6 cells/ml were seeded in 96-well plates with medium supplemented with 100 ng/ml recombinant murine G-CSF (peprotech). At day 3, medium was supplemented with fresh 100 ng/mL G-CSF. Cells and supernatant were harvested at indicated time points and supernatants analyzed for glucose levels and L-lactate levels following the Glucose (GO) Assay Kit (GAGO-20, Sigma) and the Glycolysis Cell-Based Assay Kit (#600450, Cayman Chemicals). Cells were lysed in NP40 buffer and protein was quantified using Pierce BCA Protein Assay Kit (Sigma). Glucose consumption and L-lactate levels were normalized to protein levels for each well individually. Each assay representative of at least two independent experimental repeats.

Western blot

1 × 10^6 32D CRISPR-*Atg7*$^{-/-}$ and *Atg7*$^{+/+}$ myeloblasts or after indicated time of neutrophil differentiation, or 2 × 10^6 purified primary Ly6G$^+$ neutrophils were lysed on ice using 100 μL 1 × RIPA lysis buffer. Protein concentration in supernatant was measured using BCA Protein Assay Kit (Thermo Scientific), and reducing Laemmli Sample Buffer was added to make protein samples for SDS-PAGE. 30–50 μg protein per lane were separated on 4%–12% SDS-PAGE and transferred to PVDF membrane (Millipore). After blocking with 5% skim milk, the membrane was blotted using the following primary antibodies: LC3 (L8918, Sigma) (1:1000), beta-Actin (Cell-Signaling) (1:20,000) and IRDye secondary antibodies (LI-COR, Lincoln) (1:15,000).

Microscopy

For transmission electron microscopy (TEM), 1x10^6 cells were sorted by flow cytometry based on CD11b and Ly6G expression and fixed in 2.5% glutaraldehyde, 4% PFA in 0.1M PIPES buffer, pH 7.2 for approximately 1 hr at room temperature then stored at 4°C in the fixative for 36 hr. Pellets were washed in 0.1M PIPES buffer pH 7.2 for 3x15 min, then in 100 mM glycine in the same buffer for 20 min followed by 15 min in 0.1M PIPES buffer. Cells were resuspended in warm 2.5% low melting point agarose in 0.1M PIPES and spun at 10,000 rpm for 1 min. Once set, cut agarose was incubated in 1% osmium tetroxide in 0.1M PIPES for 1 hr at 4°C, then washed with water for 20 min. Samples were incubated in 0.5% uranyl acetate in water overnight at 4°C and then taken through a graded ethanol series (30%, 50%, 70%, 80%, 90% and 95% ethanol for 10 min each on ice, then 100% ethanol for 90 min on ice, with 3 solution changes during this time). Samples were gradually infiltrated with Agar100 epoxy resin, starting with 25% resin for 1 hr, 50% resin for 2 hr, 75% resin for 1 hr and 100% resin overnight, followed by two changes with fresh 100% resin the next day before embedding by in Beem capsules and polymerization for 48 hr at 60°C. Ultrathin (90 nm) were obtained using a Leica UC7 ultramicrotome with a diamond knife (Diatome) and placed on 200 mesh copper grids, then post-stained with Reynold's lead citrate for 5 min, and imaged on a FEI Tecnai 12 TEM operated at 120 kV using a Gatan OneView CMOS camera.

For confocal microscopy, primary bone marrow neutrophil precursors were separated by flow cytometry as described and stained with Bodipy at 1000 ng/ml for 20 min at 37°C. Cells were cytospun on glass slides and mounted in the presence of DAPI (Vectashield). Images were acquired on a ZEISS 880 inverted confocal microscope + LSM120C with a 63x plan-apochromat objective. All images were taken with identical laser setup and voltage settings.

QUANTIFICATION AND STATISTICAL ANALYSIS

p values were determined by one-way or two-way ANOVA with Tukey's post-tests, unpaired Student's t test or nonparametric Mann–Whitney test as indicated in figure legends. Differences were considered statistically significant when $p < 0.05$ (*$p < 0.05$, **$p < 0.01$, ***$p < 0.001$, ****$p < 0.0001$). Data are shown as mean ± s.e.m. except indicated otherwise in figure legends. Statistics were calculated using GraphPad Prism 7 software. For all histological quantifications, histologists were 'blinded' to allocation of animals and experimental groups.

Immunity, Volume *47*

Supplemental Information

Autophagy-Dependent Generation of Free Fatty Acids Is Critical for Normal Neutrophil Differentiation

Thomas Riffelmacher, Alexander Clarke, Felix C. Richter, Amanda Stranks, Sumeet Pandey, Sara Danielli, Philip Hublitz, Zhanru Yu, Errin Johnson, Tobias Schwerd, James McCullagh, Holm Uhlig, Sten Eirik W. Jacobsen, and Anna Katharina Simon

Figure S1

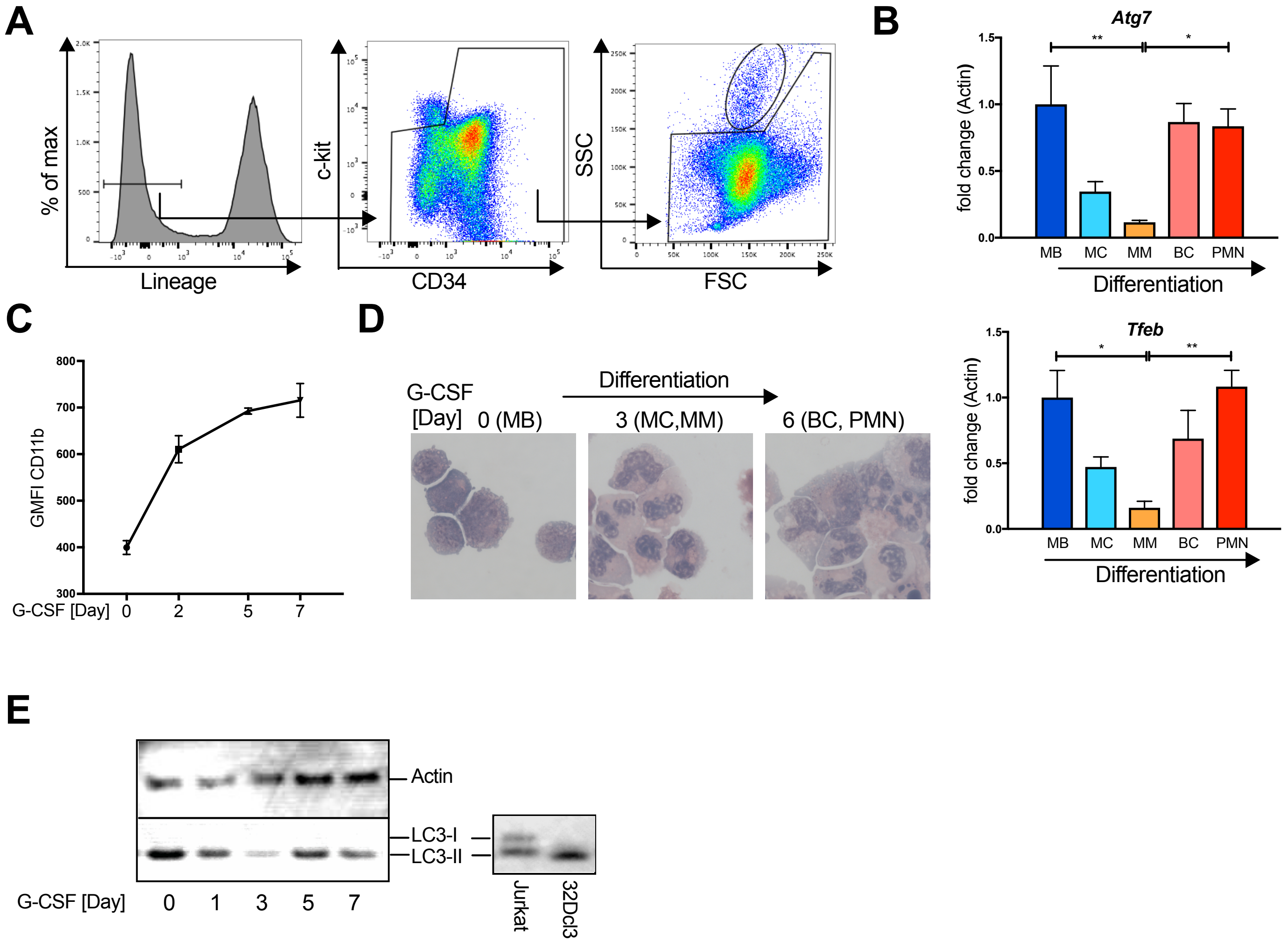
A
% of max
Lineage
c-kit
CD34
SSC
FSC
B
Atg7
fold change (Actin)
MB
MC
MM
BC
PMN
Differentiation
Tfeb
fold change (Actin)
MB
MC
MM
BC
PMN
Differentiation
C
GMFI CD11b
G-CSF [Day]
0
2
5
7
D
Differentiation
G-CSF [Day]
0 (MB)
3 (MC,MM)
6 (BC, PMN)
E
Actin
LC3-I
LC3-II
G-CSF [Day]
0
1
3
5
7
Jurkat
32Dcl3

Figure S2

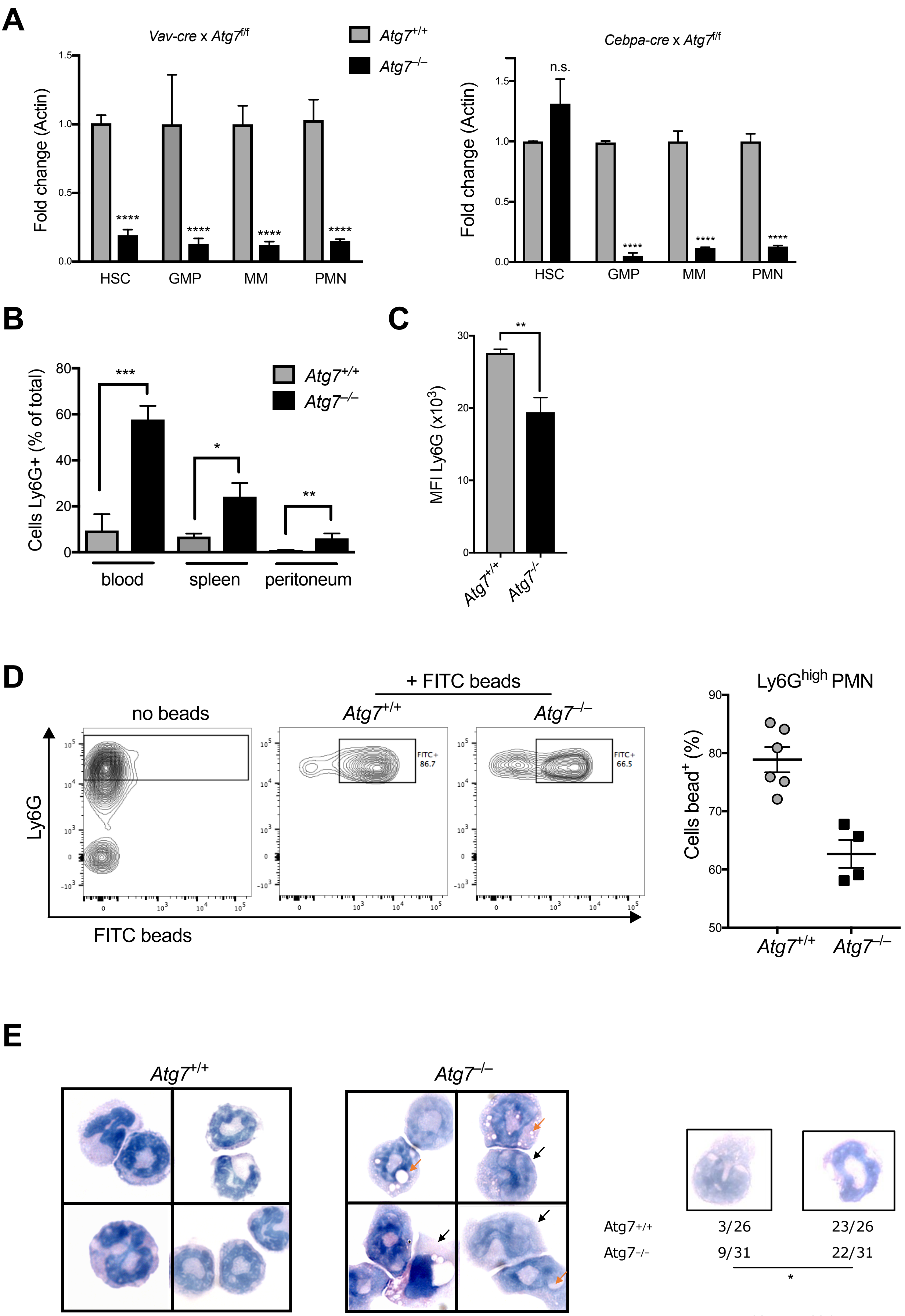

Isolated BM PMN, gated on Lin⁻ (CD4, CD5, CD8, B220, CD19, Ter119)⁻ c-kit⁻ SSC^{hi} Ly6G^{high}

Figure S3

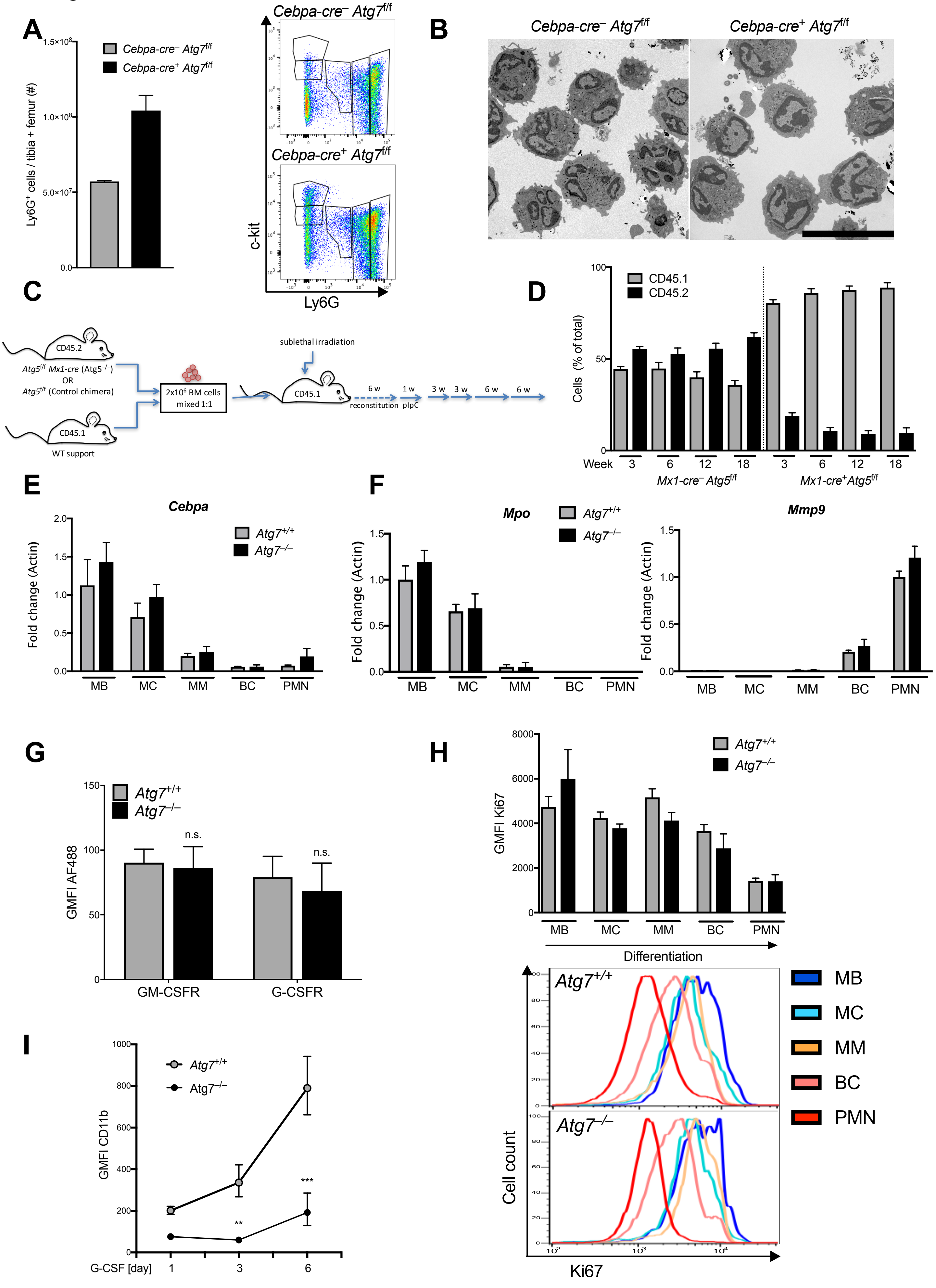
A
Cebpa-cre⁻ Atg7f/f
Cebpa-cre⁺ Atg7f/f
Ly6G⁺ cells / tibia + femur (#)
c-kit
Ly6G
B
Cebpa-cre⁻ Atg7f/f
Cebpa-cre⁺ Atg7f/f
C
CD45.2
Atg5f/f Mx1-cre (Atg5⁻/⁻)
OR
Atg5f/f (Control chimera)
CD45.1
WT support
2x10⁶ BM cells mixed 1:1
sublethal irradiation
CD45.1
6 w
reconstitution
1 w
pIpC
3 w
3 w
6 w
6 w
D
CD45.1
CD45.2
Cells (% of total)
Week
Mx1-cre⁻ Atg5f/f
Mx1-cre⁺Atg5f/f
E
Cebpa
Atg7+/+
Atg7⁻/⁻
Fold change (Actin)
MB
MC
MM
BC
PMN
F
Mpo
Mmp9
Atg7+/+
Atg7⁻/⁻
Fold change (Actin)
G
Atg7+/+
Atg7⁻/⁻
GMFI AF488
n.s.
n.s.
GM-CSFR
G-CSFR
H
Atg7+/+
Atg7⁻/⁻
GMFI Ki67
Differentiation
Atg7+/+
Atg7⁻/⁻
Cell count
Ki67
MB
MC
MM
BC
PMN
I
Atg7+/+
Atg7⁻/⁻
GMFI CD11b
G-CSF [day]
**
***

Figure S4

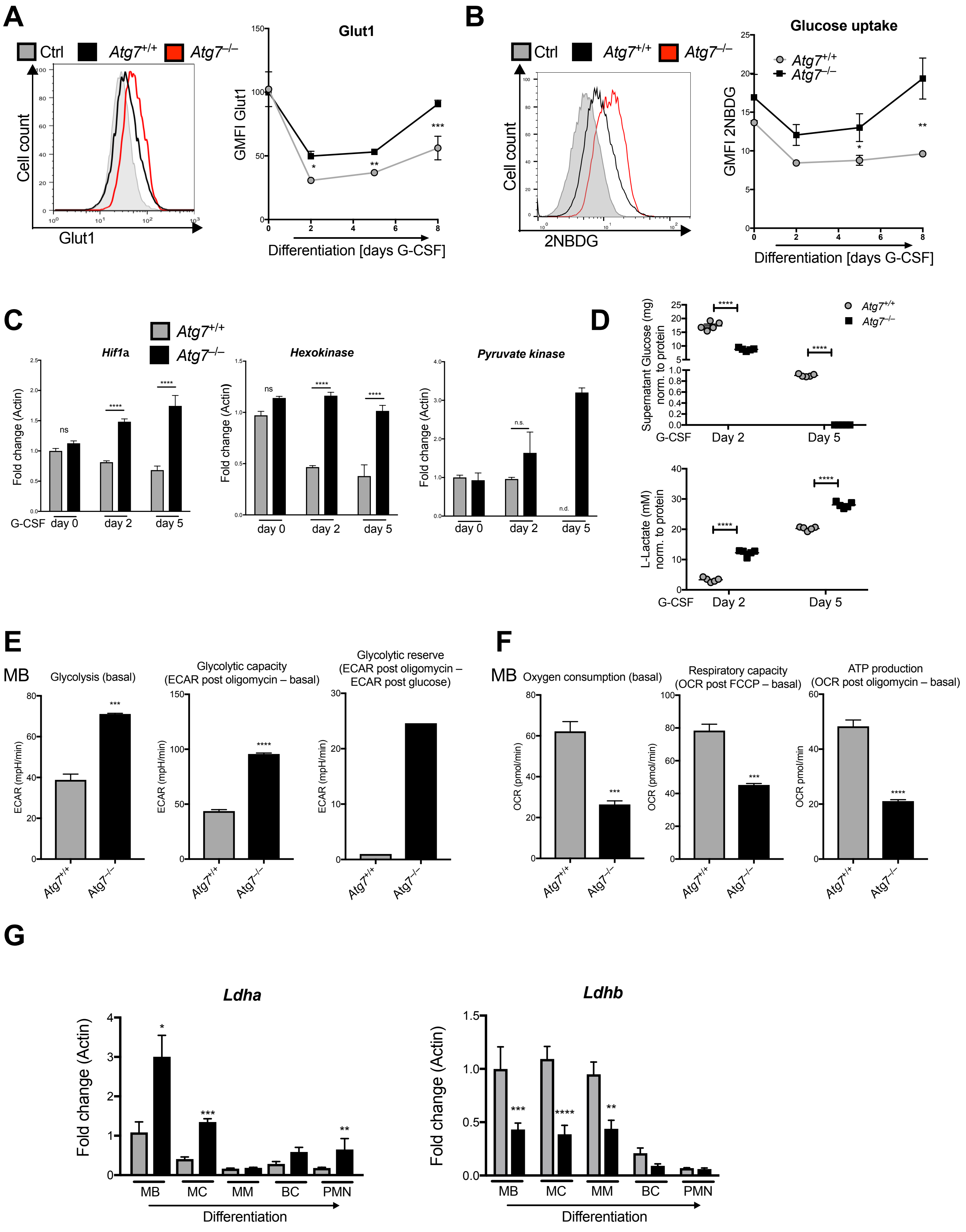

A
Ctrl
Atg7+/+
Atg7–/–
Cell count
Glut1
Glut1
GMFI Glut1
Differentiation [days G-CSF]
B
Ctrl
Atg7+/+
Atg7–/–
Cell count
2NBDG
Glucose uptake
GMFI 2NBDG
Differentiation [days G-CSF]
C
Atg7+/+
Atg7–/–
Hif1a
Hexokinase
Pyruvate kinase
Fold change (Actin)
G-CSF day 0 day 2 day 5
D
Supernatant Glucose (mg) norm. to protein
L-Lactate (mM) norm. to protein
G-CSF Day 2 Day 5
E
MB
Glycolysis (basal)
Glycolytic capacity (ECAR post oligomycin – basal)
Glycolytic reserve (ECAR post oligomycin – ECAR post glucose)
ECAR (mpH/min)
F
MB
Oxygen consumption (basal)
Respiratory capacity (OCR post FCCP – basal)
ATP production (OCR post oligomycin – basal)
OCR (pmol/min)
G
Ldha
Ldhb
Fold change (Actin)
MB MC MM BC PMN
Differentiation

Figure S5

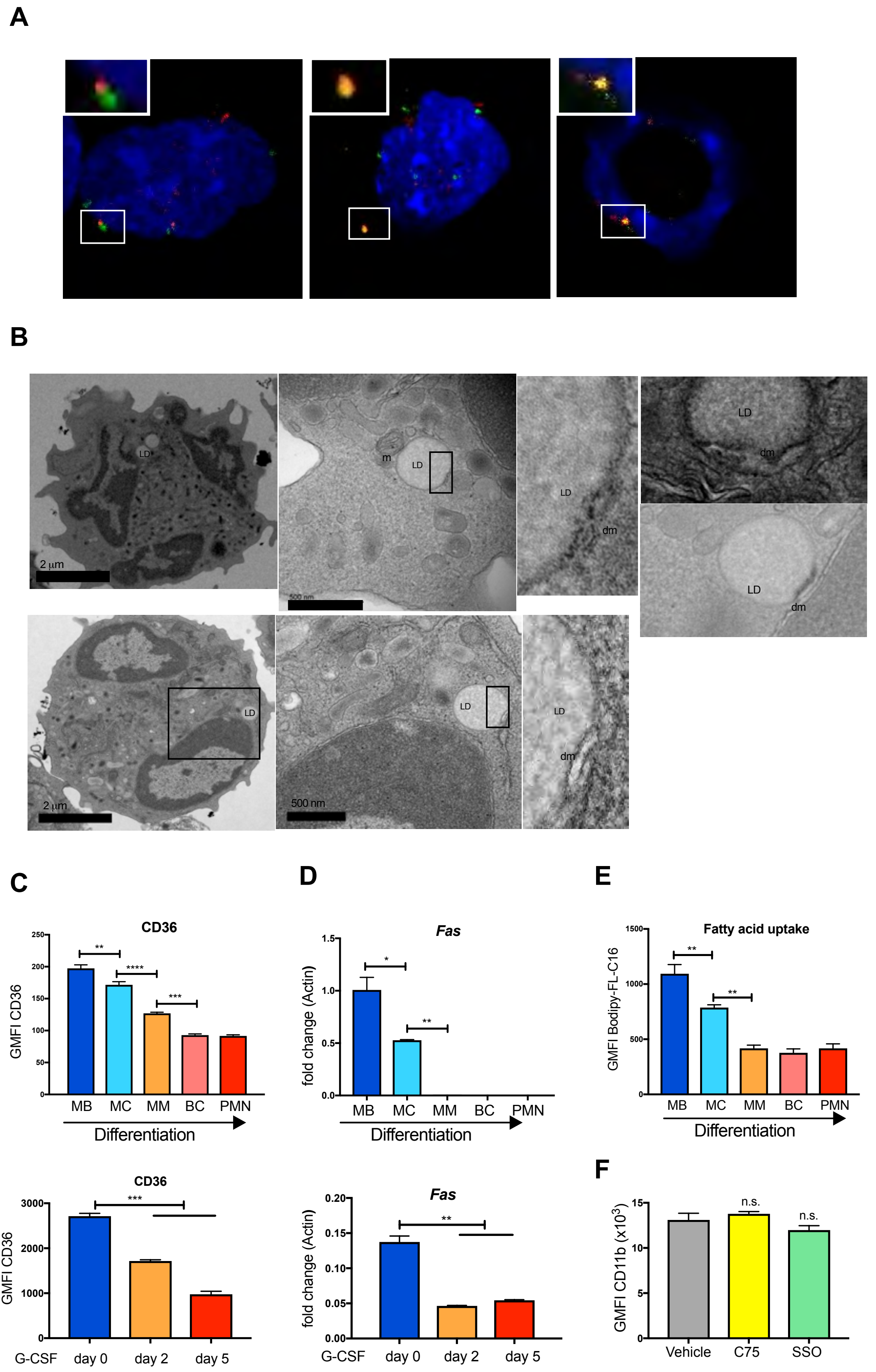
A
B
LD
2 μm
m
LD
500 nm
LD
dm
LD
dm
LD
dm
LD
2 μm
LD
500 nm
LD
dm
C
CD36
GMFI CD36
**
****
***
MB
MC
MM
BC
PMN
Differentiation
D
Fas
fold change (Actin)
*
**
MB
MC
MM
BC
PMN
Differentiation
E
Fatty acid uptake
GMFI Bodipy-FL-C16
**
**
MB
MC
MM
BC
PMN
Differentiation
CD36
GMFI CD36
***
G-CSF
day 0
day 2
day 5
Fas
fold change (Actin)
**
G-CSF
day 0
day 2
day 5
F
GMFI CD11b (x10^3)
n.s.
n.s.
Vehicle
C75
SSO

Figure S6

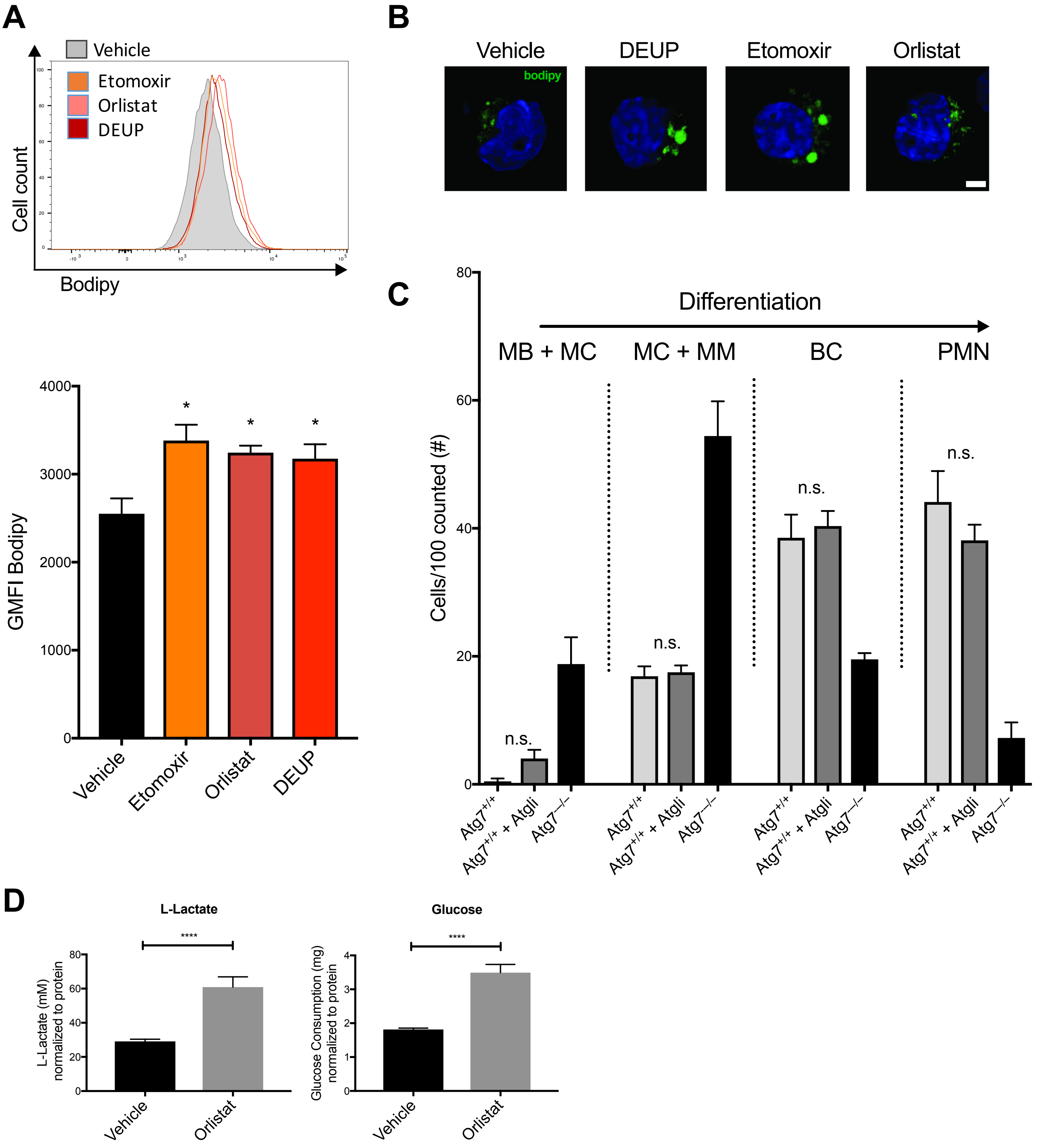
A
Vehicle
Etomoxir
Orlistat
DEUP
Cell count
Bodipy
GMFI Bodipy
B
Vehicle
DEUP
Etomoxir
Orlistat
bodipy
C
Differentiation
MB + MC
MC + MM
BC
PMN
Cells/100 counted (#)
n.s.
Atg7+/+
Atg7+/+ + Atgli
Atg7−/−
D
L-Lactate
L-Lactate (mM) normalized to protein
Glucose
Glucose Consumption (mg) normalized to protein
****
Vehicle
Orlistat

Figure S7

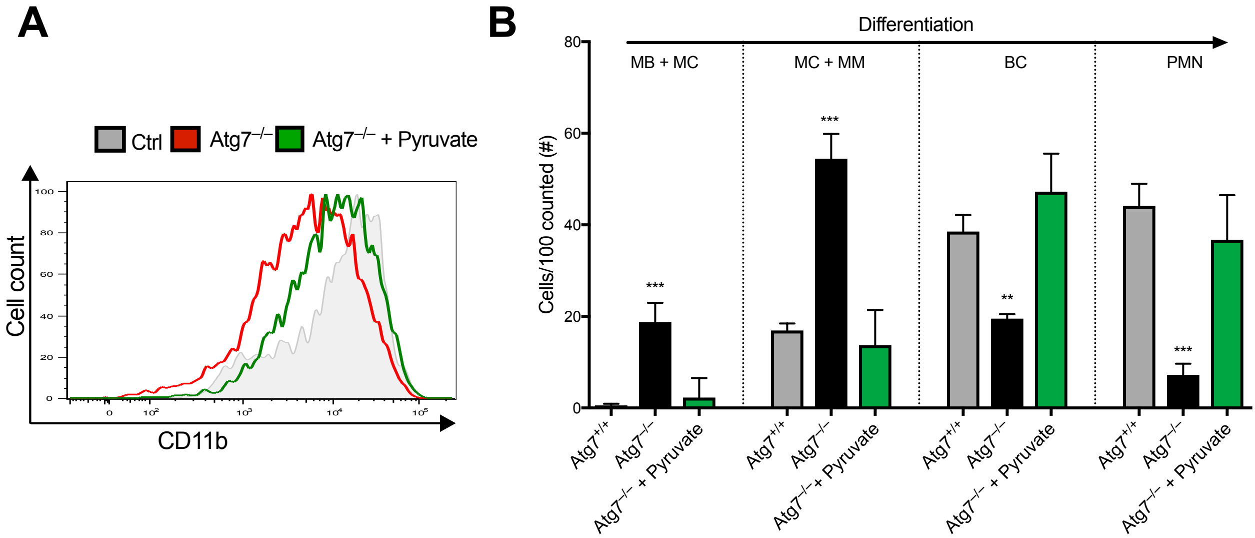
A
Ctrl
Atg7–/–
Atg7–/– + Pyruvate
Cell count
100
80
60
40
20
0
0
10²
10³
10⁴
10⁵
CD11b
B
Differentiation
MB + MC
MC + MM
BC
PMN
Cells/100 counted (#)
80
60
40
20
0
***
***
**
***
Atg7+/+
Atg7–/–
Atg7–/– + Pyruvate

	#1 MB, Myeloblast				#2 MC, Myelocyte				#3 MM, Metamyelocyte				#4 BC, Band cell				#5 PMN, Polymorphonuclear neutrophil			
**Gene name**	**Fold change (Actin) normalized to WT (ddCt)**	**SEM**	**p-value**	**Trend, significance**	**Fold change (Actin) normalized to WT (ddCt)**	**SEM**	**p-value**	**Trend, significance**	**Fold change (Actin) normalized to WT (ddCt)**	**SEM**	**p-value**	**Trend, significance**	**Fold change (Actin) normalized to WT (ddCt)**	**SEM**	**p-value**	**Trend, significance**	**Fold change (Actin) normalized to WT (ddCt)**	**SEM**	**p-value**	**Trend, significance**
Gpi1	2.073399278	0.283161894	0.0105	↑ *	1.816118651	0.375481673		↑	1.175608259	0.109539585		→	1.443392775	0.274038275		→	2.495438393	0.749486181		↑
Aldoa	3.609628316	0.111584904	0.0118	↑ *	4.411776932	1.391648427		↑	6.489416955	0.698907242		↑	2.065571554	0.622298718		↑	2.86803569	0.98502905		↑
Eno1	1.756484867	0.135989998		↑	2.082271675	0.164636881	0.0323	↑ *	3.810627023	1.575175279		↑	2.660757418	0.469373493		↑	1.228696223	0.169417275		→
Gapdh	1.399514604	0.072515204	0.0006	↑ ***	1.55370129	0.135766253	0.0024	↑ **	1.671204638	0.476902007		↑	1.384923963	0.140660851		→	1.252213077	0.03862611		→
Hif1a	1.902848332	0.242755913	0.0266	↑ *	1.542628921	0.162861848		↑	0.720169981	0.198562528		→	1.334738376	0.295744302		→	4.08330296	1.735873147		↑
Hk	2.188626205	0.098761607	0.0004	↑ ***	3.44599309	0.661468596	0.0075	↑ **	2.579311319	1.096219509		↑	1.333025928	0.069759527		→	1.369423513	0.09096196		→
Idi1	2.226968332	0.266257195		↑	1.95058024	0.596680115	0.036	↑ *	2.462855859	0.605709806	0.0298	↑ *	1.293596282	0.067110866		→	1.22281266	0.228020398		→
Ldh-a	1.867072149	0.191154741	0.0192	↑ *	2.163377481	0.232820498	<0.0001	↑ ****	1.722675878	0.435661039		↑	1.287491254	0.011897906		→	1.316770552	0.096778897		→
Pgm1	1.330845572	0.250064122		→	2.407006264	1.055989145		↑	0.974432715	0.196506941		→	1.251578728	0.239240382		→	1.214429471	0.200731166		→
Phgdh	2.523080414	0.292178362	0.0075	↑ **	1.952364371	0.136020071	0.0143	↑ *	1.033835216	0.197551989		→	1.239762606	0.156434494		→	1.700424016	0.370405524		↑
Pk	1.274671587	0.286521313	<0.0001	↑ ****	5.594022912	0.207903886	0.0011	↑ **	1.766923558	1.086311704		↑	5.531675067	0.078520555		↑	-	-		
Slc16a3	2.103559953	0.750917729		↑	3.308830242	1.405233901	0.0381	↑	1.49622697	0.15110416		→	4.134993992	1.486124866		↑	2.855671034	0.493491102	0.0301	↑ *
Slc2a1	1.228504701	0.392412635		→	2.936752528	1.65507189	0.023	↑	1.748850809	1.055313851	0.0076	↑ **	1.634300091	0.736335546	0.0048	↑ **	1.471360434	0.431790961		→
Pdk3	2.121277908	0.239228016	0.0027	↑ **	1.931352268	0.405773664		↑	1.320470108	0.242019293		→	1.537820472	0.289003191		↑	4.092738648	2.058286292		↑
Tpi1	2.213382799	0.193418355		↑	4.329457715	1.977087661	0.0452	↑	0.555276046	0.339254447		↓	1.941161689	0.495075627		↑	3.264354686	1.483787416		↑

Table S1 (related to Figure 4) Expression of glycolytic genes in BM derived neutrophil precursors from control and *Atg7*$^{-/-}$ mice.

Figure S1 Characterisation of neutrophil precursor stages (related to Figure 1)
A) Gating strategy for neutrophil precursor stages after gating on live single cells (Satake et al., 2012). Lineage cocktail includes CD4, CD5, CD8, CD19, B220, Ter119, antibodies. C-kit and CD34 are used to exclude CD34^{-} c-kit^{+} stem/progenitor cells and FSC/SSC gating excludes eosinophils.
B) Expression of *Atg7* and *Tfeb* in consecutive neutrophil differentiation stages. Data shown as fold change normalized to actin +/- SEM (ΔΔCt) (n=4 mice/group).
C) Cell surface CD11b expression as a marker to neutrophil differentiation in G-CSF stimulated 32D cells, as no monocyte/macrophage lineages develop in G-CSF condition (Valtieri et al., 1987). Surface CD11b was quantified as geometric MFI +/- SEM at the indicated days of G-CSF treatment (100 nM) from three experiments.
D) Wright-Giemsa stains showing representative morphological stages during G-CSF induced differentiation for all experiments with 32D myeloblasts. The corresponding morphological stages are indicated analogous to primary granulopoiesis.
E) Myeloblasts were cultured with 100 nM G-CSF for the indicated duration and subjected to LC3-II western blotting. The absence of LC3-I was confirmed by comparison to Jurkat lysate, representative of three experiments.

Figure S2 Mice conditionally deleted for *Atg7* accumulate abnormal neutrophils (related to Figure 2)
A) *Atg7* deletion was assessed at four distinct stages of neutrophil generation in control and *Vav-cre*$^{+}$ x *Atg7*$^{f/f}$ (left) or *Cebpa-cre*$^{+}$ x *Atg7*$^{f/f}$ (right) mouse bone marrow cells. Fold change +/- SEM was normalized to Actin and plotted relative to wildtype (ΔΔCt) (n=4 mice/group). HSC, hematopoietic stem cell; GMP, granulocyte-monocyte progenitor; MM, metamyelocyte; PMN, polymorphonuclear neutrophil.
B) Frequencies of Ly6G^{+} cells in indicated tissues as % of total live cells +/- SD (n=4 mice/group).
C) Cell surface expression of Ly6G maturation marker is plotted as MFI +/- SEM in total neutrophil lineage in control and *Atg7*$^{-/-}$ mice (*Vav-cre*$^{+}$ x *Atg7*$^{f/f}$) (n=4 mice/group)
D) Phagocytosis was measured within the mature PMN population with highest Ly6G expression, Ly6G^{high}, after 1 hour incubation with zymosan beads and 1 µg/ml LPS. Data was plotted as % of cells bead^{+} +/- SEM among the top 20% Ly6G^{high} population.
E) Wright-Giemsa stained cytospins (left) and morphological quantification of PMN and myelocytic precursor stages (right) among the mature Ly6G^{high} PMN population

(gating as indicated). Black arrows indicate myelocytes; orange arrows indicate droplets. Significance tested by Fisher´s exact test, *$p<0.05$.

Figure S3 *Cebpa-cre*$^{+}$ x *Atg7*$^{f/f}$, *Vav-cre*$^{+}$ x *Atg7*$^{f/f}$ and *Mx1-cre*$^{+}$ x *Atg5*$^{f/f}$ chimeric mice confirm a cell intrinsic neutrophil differentiation defect (related to Figure 3)

A) Absolute number of Ly6G^{+} cells in *Cebpa-cre*$^{–}$ x *Atg7*$^{f/f}$ and *Cebpa-cre*$^{+}$ x *Atg7*$^{f/f}$ mouse bone marrow (n=4 mice) and example images of precursor stage gating for all experiments involving precursor gating.

B) Representative electron micrographs of CD11b^{+} Ly6G^{+} BM cells from the indicated genotypes from two independent experiments.

C) Schematic of experimental set-up for mixed, inducible hematopoietic bone marrow chimeras. pIpC was injected i.p. (250 µg pIpC in 250 µL saline) e.o.d. for one week to induce excision.

D) Peripheral blood reconstitution with donor (CD45.2) and recipient (CD45.1) type cells shown as % of total cells +/- SD at the indicated weeks after pIpC-induced *Atg5* deletion.

E-F) Gene expression of the myeloid transcription factor *Cebpa* (E) and the early and late granule genes *Mpo* and *Mmp9* (F) in control and *Vav-cre*$^{+}$ x *Atg7*$^{f/f}$ mice (n=4 mice/group) after separation of the indicated precursor populations and normalization to *Actin* relative to control (ΔΔCt).

G) geometric MFI +/- SEM from control and *Vav-cre*$^{+}$ x *Atg7*$^{f/f}$ for the cell surface expression of G-CSF and GM-CSF receptors (n=4 mice/group).

H) Cell cycle exit was measured as geometric MFI of Ki67 expression (top) by intracellular flow cytometry in the indicated precursor populations of control (*Vav-cre*$^{–}$ x *Atg7*$^{f/f}$) and *Vav-cre*$^{+}$ x *Atg7*$^{f/f}$ mice (n=3 mice/group); Ki67 histograms are shown for each genotype (bottom).

I) CD11b maturation marker cell surface expression after culture of *Atg7*$^{+/+}$ and *Atg7*$^{–/–}$ myeloblasts for the indicated duration with G-CSF to induce differentiation (100 nM). Data are geometric MFI of CD11b +/- SEM, representative of four experiments. The same data is shown as histograms in Figure 3 H.

Figure S4 Excessive glycolysis and perturbed mitochondrial respiration without autophagy is conserved *in vitro* and *in vivo* (related to Figure 4)

A-E) CRISPR *Atg7*$^{+/+}$ and *Atg7*$^{–/–}$ myeloblasts (derived from 32D cells) were maintained in the presence of IL-3 (10 ng/µl) or cultured in G-CSF (100 nM) for the indicated duration.

A+B) Representative histogram (left) and quantification (right) of Glut-1 expression (A) and 2NBDG uptake (B) as indication of glycolytic activity. Tinted grey histograms are FMO= “fluorescence minus one” baseline FITC fluorescence control. Quantification on the right shows the geometric MFI +/- SEM in *Atg7*$^{+/+}$ and *Atg7*$^{–/–}$ cells at indicated timepoints. Data from three independent biological replicates from two experiments, *$p < 0.05$, **$p < 0.01$, ***$p < 0.001$.

C) Gene expression analysis of the indicated glycolytic genes in 200 myeloblasts during G-CSF induced differentiation. Data from four biological replicates measured in technical triplicates in one experiment, representative of two experiments. Fold change +/- SEM of biological replicates is shown normalized to actin relative to day 0 control ($\Delta\Delta$Ct), ****p < 0.0001.

D) Enzymatic quantification of glucose consumption (top) and lactate accumulation in the supernatant (bottom) after the indicated time of G-CSF induced neutrophil differentiation of 32D myeloblast precursors. Concentrations were determined based on a standard curve and normalized to protein content of each culture.

E) Quantification of glycolytic parameters from Figure 4H. Extracellular acidification rate is shown as mpH/min +/- SEM, data are from four biological replicates from two independent CRISPR $Atg7^{-/-}$ clones and $Atg7^{+/+}$ controls at the myeloblast stage cultured in IL-3. ***p < 0.001, ****p < 0.0001.

F) Quantification of mitochondrial metabolism assay from Figure 4I. Oxygen consumption rate (OCR) is shown as pmol/min +/- SEM, data are from four biological replicates from two independent CRISPR $Atg7^{-/-}$ clones and $Atg7^{+/+}$ control, ***p < 0.001, ****p < 0.0001.

G) Extracellular acidification rate is shown as mpH/min +/- SEM from 4-6 independent CRISPR $Atg7^{-/-}$ cultures and $Atg7^{+/+}$ controls after the indicated duration of G-CSF mediated differentiation. *p < 0.05, **p < 0.01.

H) Gene expression analysis of lactate dehydrogenase isoforms (*Ldha,* lactate generation*; Ldhb,* pyruvate generation). Five neutrophil precursor populations were sorted by flow-cytometry from $Atg7^{+/+}$ ($Vav\text{-}cre^{-}$ x $Atg7^{f/f}$) or $Atg7^{-/-}$ ($Vav\text{-}cre^{+}$ x $Atg7^{f/f}$) mice (100 cells/population, n=4-5 mice/group). Fold change +/- SEM of biological replicates is plotted normalized to actin relative to MB control ($\Delta\Delta$Ct). *p < 0.05, **p < 0.01, ***p < 0.001, ****p < 0.0001.

Figure S5 Lipophagy, lipid synthesis and -uptake during neutrophil differentiation (related to Figure 5)

A) Neutrophil precursors were purified from bone marrow of LC3-GFP transgenic mice by flow cytometry and stained with LD540 lipid droplet dye. Different representative stages of lipophagy were observed; left, direct contact with partial overlap of lipid droplet and $LC3^{+}$ vesicle; middle, complete colocalizaiton of lipid and LC3 signal; right, dispersed lipid signal around double positive vesicle.

B) Neutrophil precursors were purified from bone marrow of wildtype mice by flow cytometry and prepared for electron microscopy to visualize the formation of membraneous structures around lipid droplets (See also (Singh et al., 2009).

C) Cell surface expression of the lipid uptake receptor CD36 in five consecutive neutrophil differentiation stages from wildtype mouse BM (n=3 mice/group) (top) and during 5 days of G-CSF induced neutrophil differentiation of 32D myeloblast precursors (bottom). Data shown as geometric MFI +/- SEM, significance tested by one-way Anova. **p < 0.01, ***p < 0.001, ****p < 0.0001.

D) Expression of fatty acid synthase (*Fasn*) in five consecutive neutrophil differentiation stages sorted by flow-cytometry from wildtype mouse BM (n=2 mice/group) (top) and during 5 days of G-CSF induced neutrophil differentiation of 32D myeloblast precursors (bottom). Data shown as fold change +/- SEM normalized to *Actin* relative to first stage (ΔΔCt), significance tested by one-way Anova. *p < 0.05, **p < 0.01.
E) Uptake of the labelled fatty acid Bodipy-FL-C16 in neutrophil differentiation stages from wildtype mouse BM (n=2 mice/group) 2 hours after i.v. injection of Bodipy-FL-C16 (25 mg/kg). Data shown as geometric MFI +/- SEM, significance tested by one-way Anova. **p < 0.01.
F) Cell surface expression of CD11b maturation marker after 5 days of G-CSF induced neutrophil differentiation of 32D myeloblast precursors in the presence of the specific *Fas*-inhibitor C75 or the CD36 inhibitor SSO. n.s., not significant. Data shown as geometric MFI +/- SEM, significance tested by student´s T-test.

Figure S6 Lysosomal, but not cytosolic lipases are essential for granulopoiesis (related to Figure 6)
A) Bodipy histograms (top) and quantification as geometric MFI +/- SEM (bottom) after the indicated drug treatments for five days during G-CSF induced differentiation confirms the accumulation of lipid droplets (See also Figure 6C). Representative of three independent experiments.
B) Confocal micrographs of bodipy$^+$ lipid droplet accumulation in response to the indicated drug treatments (See also supplemental methods). Scale bar, 5 µm.
C) Wild type myeloblasts were differentiated in the presence or absence of the cytosolic lipase inhibitor atglistatin (Atgli) with G-CSF for 7 days. *Atg7*$^{–/–}$ for comparison. Data shown as number of cells in each morphological group per 100 cells counted +/- SEM. n.s., non-significant.
D) Enzymatic quantification of lactate accumulation in the supernatant (left) and glucose consumption (right) after 5 days of G-CSF induced neutrophil differentiation of 32D myeloblast precursors in the presence of the lipase inhibitor orlistat. Concentrations were determined based on a standard curve and normalized to protein content of each culture.

Figure S7 Pyruvate rescues differentiation in *Atg7*$^{–/–}$ granulopoiesis (related to Figure 7)
A) Representative histograms of CD11b expression for three experiments after 5 days G-CSF (100 nM) shows rescue of CD11b expression in *Atg7*$^{–/–}$ cells in the presence of pyruvate (green).
B) Histological quantification of indicated differentiation stages shows a near complete rescue of neutrophil differentiation block in *Atg7*$^{–/–}$ cells in the presence of pyruvate (2 mM, green). Data shows number of cells/100 cells counted per group, from four biological replicates representative of two independent clones from one experiment, representative of three experiments.
